# Comparison of Methods for Incorporating Related Data When Developing Clinical Prediction Models: A Simulation Study

**DOI:** 10.1002/bimj.70166

**Published:** 2026-08-27

**Authors:** Haya Elayan, Matthew Sperrin, Glen P. Martin, Frieder Braunschweig, Jonas Faxén, Joakim Alfredsson, David A. Jenkins

**Affiliations:** ^1^ Division of Informatics, Imaging and Data Science, Faculty of Biology, Medicine and Health University of Manchester Manchester UK; ^2^ Department of Cardiology Karolinska University Hospital Stockholm Sweden; ^3^ Department of Physiology and Pharmacology Karolinska Institute Stockholm Sweden; ^4^ Department of Health, Medicine and Caring Sciences Linköping University Linköping Sweden

**Keywords:** Clinical Prediction Models, data distribution shift, data heterogeneity, including related data, simulation study

## Abstract

Clinical Prediction Models (CPMs) compute an individual's risk of an outcome, given a set of predictors. Guidance states CPMs should be constructed using data sampled from the target population. However, researchers might have access to ancillary data sets from different time points, countries, or healthcare settings, which could support model development. This study explores in which situations the ancillary data affect CPM performance, given potential heterogeneity. We conducted a simulation study to assess the impact of heterogeneity between the target and the ancillary data sets, and their relative sample size, on CPM performance. Target and ancillary populations were generated with varying degrees of heterogeneity. CPMs were developed using target‐only logistic regression, logistic and intercept updating, and importance weighting using propensity scores. These models were evaluated on independent data using calibration, discrimination, and prediction stability. Also, a real‐world case study was used as an illustrative example of application using the SWEDEHEART registry. Incorporating ancillary data generally improve CPM performance. Logistic and Intercept Recalibration often outperformed the target‐only regression approach. However, Logistic Recalibration showed greater variability and instability in calibration, while Intercept Recalibration performed poorly under predictor–outcome association shift. The importance weighting method demonstrated consistent performance across a wide range of scenarios and appears to be a reliable alternative, particularly in practical settings where the presence and type of data distribution shift is often unknown.

## 1 Introduction

Clinical Prediction Models (CPMs) are mathematical algorithms that compute the risk of a diagnostic or prognostic outcome, given a set of predictors related to a patient, disease, or treatment (Steyerberg 2009). These models have become helpful tools in clinical practice, providing healthcare professionals with valuable insights that inform decision‐making. CPMs are widely used in various medical domains. For example, in cardiac surgery, EuroSCORE model estimates the risk of mortality after a cardiac surgery, (Nashef et al. 2012). In oncology, the Nottingham Prognostic Index predicts survival for breast cancer patients after surgery (Galea et al. 1992). Similarly, in critical care, APACHE III assesses the risk of hospital mortality for intensive care unit (ICU) patients (Knaus et al. 1991). The development of these models involves selecting relevant predictors, applying appropriate methods, and validating the models' performance to ensure their reliability and utility in clinical practice.

When developing CPMs, the primary objective is to create tools that improve risk assessment within a specific target population where the model is intended to be deployed (Sperrin et al. 2022; Steyerberg et al. 2004; Collins, Dhiman, et al. 2024). Guidance states models should be constructed using data directly sampled from the target population (Collins, Moons, et al. 2024; Riley et al. 2023). However, researchers might also have access to additional and potentially related data sets, which we hereto refer to as the ancillary data, originating from different time points, countries, healthcare settings, regions, or hospitals, which might help with model development, particularly if the target data set—the portion of the development data sampled from the target population—have a small sample size (Riley et al. 2020). A critical consideration in this context is the degree of heterogeneity between the target and the ancillary data sets, which reflects differences in their underlying distributions. This heterogeneity may arise for several reasons, including differences in the distribution of predictors (i.e., Case‐mix shift), event prevalence (i.e., event rate shift), or in the relationships between predictors and outcome prevalence (i.e., predictor–outcome association shift) (Debray et al. 2015; Altman and Royston 2000; Toll et al. 2008; Moons et al. 2009; Ramspek et al. 2021; Cabitza et al. 2021). These changes, commonly referred to as data distribution shift, can result from factors such as differences in population age distributions, socioeconomic and ethnic composition, and shifting risk factor profiles over time. Other contributing elements include variations in clinical practice, such as provider experience, coding standards, electronic health record (EHR) interfaces, and data definitions. Such shifts can negatively impact model performance, leading to inaccurate predictions when applied to the target population (Davis et al. 2019; Lekadir et al. 2025; Moreno‐Torres et al. 2012; Efthimiou et al. 2024; Guo et al. 2021; Feng et al. 2022). A common approach to handle these issues is to apply model updating methods to improve CPM's prediction. For instance, the APACHE III‐j model, initially developed using ICU data from the United States, overestimated mortality risk for Japanese ICU patients by more than twofold. However, once recalibrated with Japanese ICU data, the model's predictive accuracy improved (Endo et al. 2021). Additionally, the National Acute Kidney Injury Risk Prediction model, which is annually retrained by developing a new model based on data from the previous year, performed worse than the surveillance‐based approach that employs various model updating techniques and incorporates data from previous time points (Davis et al. 2022). Similarly, QRISK has been annually updated and recalibrated with the latest data from the QResearch database to ensure the model stays aligned with improvements in data quality and shifts in population characteristics such as changes in prevalence of smoking, aging of the population, or declining incidence of cardiovascular disease (Hippisley‐Cox et al. 2017).

The aim of this paper is to assess in what situations and using which methods, the ancillary data help (and harm) model performance when incorporated in the development phase. Here, development data refer to all data used for model building, including both target and ancillary data sets. Specifically, if the effectiveness of utilizing the ancillary data is influenced by the degree of heterogeneity between the target and the ancillary data sets caused by data distribution shift, and the relative sample sizes of these data sets. To examine this, we conducted a simulation study to assess the impact of these factors on CPMs performance. Target and ancillary populations were generated with varying degrees of heterogeneity, caused by data distribution shift. CPMs were developed using target‐only logistic regression (developed on data from target only), logistic and intercept regression updating methods (developed on the ancillary data or ancillary and target data, then updated to target), and importance weighting using propensity scores (developed on all available data from ancillary and target, while weighting the ancillary data samples based on their similarity to the target). These models were then validated on independent data drawn from the same data‐generating mechanism as the target population, using calibration, discrimination, and prediction stability metrics. Furthermore, a real‐world case study from the SWEDEHEART registry was used as an illustrative example of application, focusing on myocardial infarction (MI) patients with out‐of‐hospital cardiac arrest within 90 days as the outcome. In the end, we will provide a list of key take home messages regarding enhancing CPM performance when incorporating ancillary data in model development.

## 2 Methods

### 2.1 Aims

This simulation study, reported according to the ADEMP structure, proposed by Morris et al. (2019), aims to assess and compare the predictive performance of different CPM methods for incorporating the ancillary data when developing CPMs. Target and ancillary populations were generated with varying degrees of heterogeneity, caused by data distribution shift. We will take the following steps:

1. Simulate various types of data distribution shifts, including Case‐mix shift, event rate shift, predictor–outcome association shift, and combination of different types of shift.
2. Develop multiple CPMs using methods that are designed to incorporate the ancillary data while account for the data distribution shift in the development data set.
3. Compare the performance of the methods across different data distribution shift types and sample sizes using appropriate evaluation metrics.

### 2.2 Data‐Generating Mechanisms

#### 2.2.1 Data Distribution Shift

**FIGURE 1:**
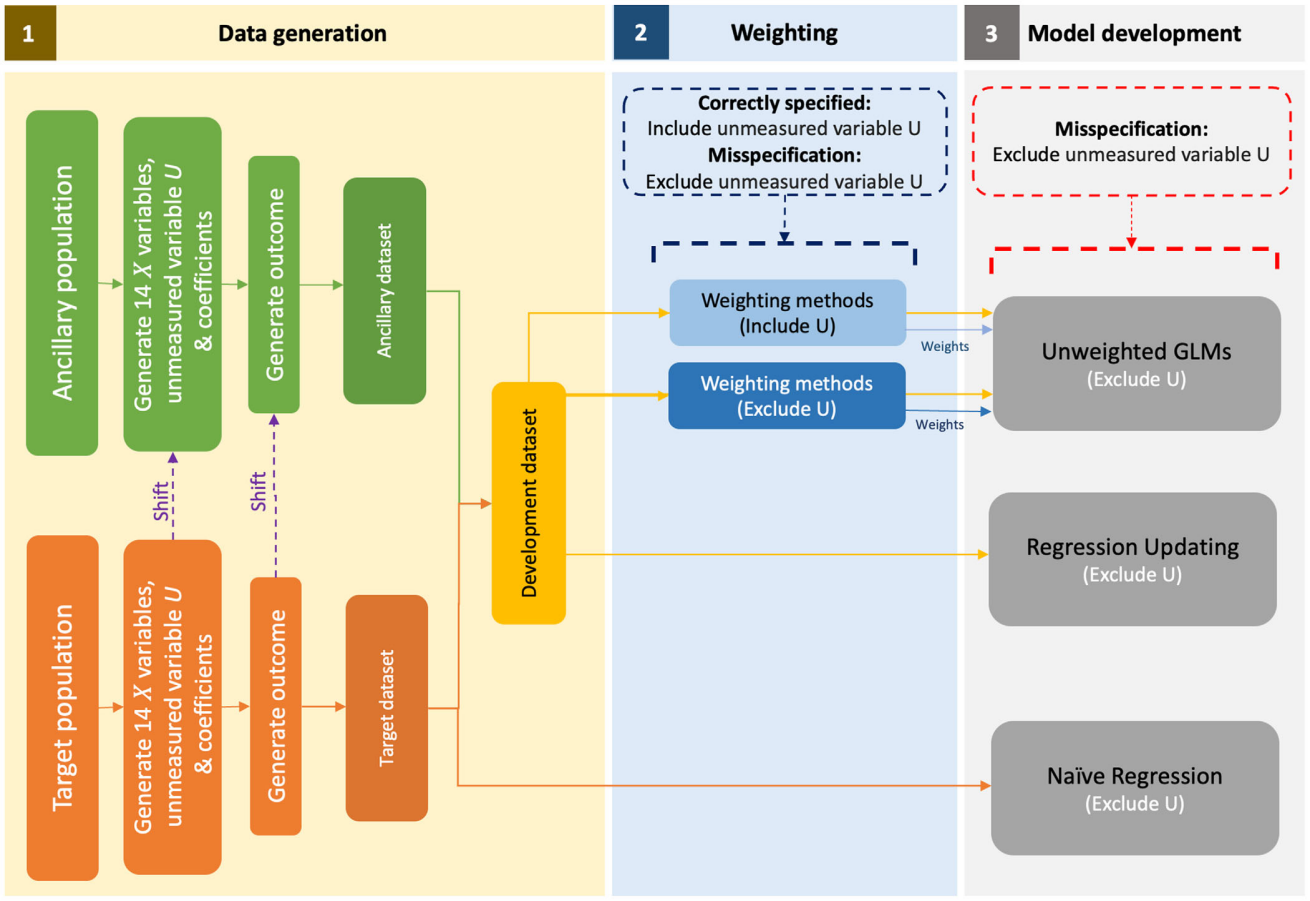
Simulation study design.

Let P(X,Y) denote the joint distribution of the development data set, where X denotes an N×K matrix of predictor variables, and Y denotes a vector of length N of outcomes. Here, N denotes the size of the development data set, including target and the ancillary data sets combined, as illustrated in Figure 1. Let R denote an indicator for the data being from a certain population, where R=0 indicates an individual from the target population where the model will be deployed, and R=1 indicates an individual from the ancillary population.

Data distribution shift occurs when differences arises between the subset sampled from the target population and the subset sampled from the ancillary population, such that:

(1) $$\begin{array}{cc} P(X,Y|R=0) & \neq P(X,Y|R=1). \end{array}$$

Accordingly, data distribution shift can occur in the components of the joint distribution of the development data set P(X,Y) as:

- A shift in the probability density of the input P(X), caused by shift in the distribution of individual predictors, but the conditional probability of the output given the input P(Y|X) remains the same, also known as Case‐mix shift, such that:
(2) $$\begin{array}{cc} P(X|R=0) & \neq P(X|R=1),\\ P(Y|X,R=0) & =P(Y|X,R=1). \end{array}$$
- A shift in the conditional probability of the output given the input, P(Y|X), while the probability density of the input, P(X), remains the same. This can occur in two different ways:
  ‐ **Event rate shift:** the shift in the conditional probability of the output given the input P(Y|X) is caused by a shift in the outcome rate.
  ‐ **Predictor–outcome association shift:** the shift in the conditional probability of the output given the input P(Y|X) is caused by a shift in the strength of associations between predictors and outcome.
(3) $$\begin{array}{cc} P(X|R=0) & =P(X|R=1),\\ P(Y|X,R=0) & \neq P(Y|X,R=1). \end{array}$$
- A shift in the conditional probability of the output given the input P(Y|X), and in the probability density of the input P(X). Whether the shift in P(Y|X) is caused by a shift in the outcome rate or a shift in the strength of associations between predictors and outcome, such that:
(4) $$\begin{array}{cc} P(X|R=0) & \neq P(X|R=1),\\ P(Y|X,R=0) & \neq P(Y|X,R=1). \end{array}$$

We simulate the shift between the ancillary and target populations as the following:

1. **No shift**: We generate the ancillary population from same distribution as the target population.
2. **Case‐mix shift**: We generate the ancillary population from a distribution that differs from the target population distribution by a shift in mean, variance, and/or event prevalence of some predictors.
3. **Predictor–outcome association shift**: We generate the ancillary population from a distribution that differs from the target population by a shift in coefficients of some predictors.
4. **Event rate shift**: We generate the ancillary population from a distribution that differs from the target population by a shift in intercept. We shift the intercept rather than shifting the outcome prevalence because shifting the intercept allows to test the “predictor–outcome association shift only” scenario.
5. **Combination of different types of shifts**: We generate the ancillary population from a distribution that differs from the target population by introducing the shift in all possible combinations of the following simulation parameters: predictor variables' means, predictor variables' variances, predictor variables' event prevalence, predictor variables' coefficients, and the intercept.

#### 2.2.2 Predictors Generation

We generate measured predictor variables (X) and an unmeasured predictor variable (U) for the target population using the predictor‐generation model in Equation (5), derived from a multivariate normal distribution:

(5) $$X_{target}={\left(X_{1},X_{2},\text{…},X_{14},U\right)}^{T}∼MVN\left(\mu_{target},\Sigma_{target}\right).$$

The target mean vector, $\mu_{target}$, is set to zero, and the target covariance matrix, $\Sigma_{target}$, is defined based on clinically plausible covariance values.^1^ Also, we dichotomize five variables ($X_{10},X_{11},X_{12},X_{13},X_{14}$), using a threshold from the variable's distribution. Values above the threshold are assigned 1, and those below are assigned 0, with an event prevalence (p) defined by clinically plausible values. Please refer to the Supporting Information for the defined covariance matrix and the binary predictors prevalence (Tables S1 and S2).

Furthermore, we generate predictor variables for the ancillary population using the predictor‐generation model in Equation (6):

(6) $$X_{ancillary}∼MVN(\left(\mu_{target}+\Delta \mu \right),\left(\Sigma_{target}+\Delta \Sigma \right)),$$

where the shift in the selected distribution parameters Δμ and ΔΣ and the event prevalence p of the dichotomized variables are listed in Table 1. The shift in means and event prevalence of certain predictor variables is based on clinically plausible shifts, reflecting a real‐world scenario,^2^ while the shift in variances is based on a simulation study conducted by Davis et al. (2019), whose work is highly relevant to our study. Here, increasing or decreasing the variability of predictor distributions induces less or more heterogeneity between the ancillary and target populations.

**TABLE 1:** Shift in simulation parameters.

Parameter	Shift in parameter
$\mu_{X_{1}}$ = 0, $\sigma_{X_{1}}^{2}$ = 1	$\Delta \mu_{X_{1}}$ = [0, 0.8, 4], $\Delta \sigma_{X_{1}}^{2}$ = [0, 0.2, 2]
$\mu_{X_{2}}$ = 0	$\Delta \mu_{X_{2}}$ = [0, 1]
$p_{X_{10}}$ = 0.3	$\Delta p_{X_{10}}$ = [0, 0.1]
$\beta_{0,target}$=‐3.4	$\Delta \beta_{0}$=[0, 0.9]
$\beta_{1,target}$ =0.5	$\Delta \beta_{1}$ =[0, 0.4]
$\beta_{U,target}$=0.2	$\Delta \beta_{U}$ =[0, 0.4]

#### 2.2.3 Outcome Generation

We use three different outcome‐generation models that vary the number and type of predictor variables for the target population to generate a binary outcome Y, then we add shift to each model to generate outcomes for the ancillary population. We utilize the resulting data sets to evaluate models as a function of the number of predictor parameters.

1. Model with K=14 included measured predictor variables X **(Mixed‐types)** and an unmeasured predictor variable U for the target and the ancillary populations defined by Equations (7) and (8), respectively.
(7) $$\begin{array}{cc} & P\left(Y=1|X,U,R=0\right)=\pi_{\text{target}}\\ & ={\left[1+\text{exp}\left(-\left(\beta_{0,target}+\sum_{k=1}^{K}\beta_{k,target}X_{k}+\beta_{U,target}U\right)\right)\right]}^{-1}, \end{array}$$
(8) $$\begin{array}{cc} & P\left(Y=1|X,U,R=1\right)=\pi_{\text{ancillary}}\\ & =[1+\text{exp}(-(\left(\beta_{0,target}+\Delta \beta_{0}\right)+\sum_{k=1}^{K}(\beta_{k,target}\\ & \;{+\Delta \beta_{k})X_{k}+\left(\beta_{U,target}+\Delta \beta_{U}\right)U))]}^{-1}. \end{array}$$
2. Model with K=4 included measured predictor variables X **(Mixed‐types)** and an unmeasured predictor variable U. The coefficients of excluded predictors from Equations (7) and (8) were set to 0 reducing the model to $P(Y=1|X_{1},X_{2},X_{10},X_{11},U,R=0)$ for the target population and $P(Y=1|X_{1},X_{2},X_{10},X_{11},U,R=1)$ for the ancillary population.
3. Model with K=3 included measured predictor variables X **(Continuous only)** and an unmeasured predictor variable U. The coefficients of excluded predictors from Equations (7) and (8) were set to 0 reducing the model to $P(Y=1|X_{1},X_{2},X_{3},U,R=0)$ for the target population and $P(Y=1|X_{1},X_{2},X_{3},U,R=1)$ for the ancillary population.

The choices for the models' coefficients (conditional log‐odds ratios) are based on clinically plausible values, reflecting what might be expected in real‐world example, subsequently adjusted to achieve a blackAUC averaging 0.75 for generating sufficient sample size. Moreover, the choice of $\beta_{0}$ values for the target population in each model generates an overall outcome prevalence of approximately 10%, and adding $\Delta \beta_{0}$ increases the prevalence to approximately 20%. Coefficient choices for target population and the shift in selected coefficient choices for the ancillary population are listed in the Supporting Information in Table 1 and Table S3.

#### 2.2.4 Development Data Set and Sample Size

Throughout all the simulation iterations across all simulation scenarios, we generate large population‐level data sets—comprising a total of 100,000 observations—based on the outcome‐generation model for both the target and ancillary populations. The goal is to simulate overarching populations from which random samples can subsequently be drawn for developing a CPM.

Each population‐level data set of size N is a combination of samples drawn from the ancillary population subset of size $N_{ancillary}$ and target population of size $N_{target}$, where $N=N_{ancillary}+N_{target}$, with varying proportions of splits: 25% ancillary: 75% target, 50% each, and 75% ancillary: 25% target. Subsequently, we calculate the minimum development sample size ($N_{min}$) using the criteria outlined by Riley et al. (2019) for model development. As the criteria requires to prespecify a sensible value for the Cox–Snell $R^{2}$, we fit a logistic regression model on each population‐level data set, and obtain the Cox–Snell $R^{2}$ for use in the sample size calculations by Riley et al. (2021). Following this, we randomly sample development data sets of size $N=N_{min}$ by combining the ancillary and target subsets on varying proportions splits, to represent data sets available to develop a CPM. Furthermore, we explore different sample sizes for each development data set, as when $N=N_{min}$ neither the target nor the ancillary data set meets the required sample size in any scenario. Therefore, we also consider larger sample sizes, including twice the minimum sample size $\left(2\times N_{min}\right)$ and four times the minimum sample size $\left(4\times N_{min}\right)$ to assess their impact on model development where at least one of the data set or both meets the required sample size. This resulted in 2592 simulation scenarios, each of which were run across 200 iterations of the above data‐generating processes.

### 2.3 CPM Development Methods

We focus on logistic regression‐based CPMs to estimate the probability of the binary outcome Y, conditional on the set of X predictors for each of the data‐generating processes. We fit the CPMs using the following development and updating methods:

#### 2.3.1 Develop CPM on Target Only

This method uses the standard unweighted logistic regression to develop a CPM on the target only. Let individual i from target, the unknown parameters of the CPM (log‐odds), $\beta_{0},\beta_{1},\text{…},\beta_{K}$, be estimated by maximizing the following log‐likelihood (LL) function:

(9) $$\text{LL}=\sum_{i=1}^{N_{target}}Y_{i}\times \log\left(P_{i}\right)+\left(1-Y_{i}\right)\times \log\left(1-P_{i}\right),$$

where P is the predicted probability for a binary outcome.

#### 2.3.2 Model Recalibration: Updating the Intercept Only

One of discrete model updating methods that uses the new available data to update the model is Recalibration by updating the intercept only, which intends to correct “calibration‐in‐the‐large,” that is, make the average predicted probability equal to the observed overall event rate (Vergouwe et al. 2017; Steyerberg 2009). We implement this method in two variations as follows:

1. **Develop CPM on all data and update on target only**: In this variation, we first develop a logistic regression model on both ancillary and target (full development data set), referred as “full model,” then we apply the model to the target data to obtain the linear predictor ($LP_{target}$) for all individuals. Next, we then update the model by fitting a logistic regression on the target data with $LP_{target}$ as an offset variable (the regression coefficient is constrained to be 1).
2. **Develop CPM on ancillary and update on target only**: As an alternative, we first develop a logistic regression model on ancillary, referred as “ancillary model,” then we apply the model to the target data to obtain the linear predictor ($LP_{target}$) for all individuals. Next, we update the model by fitting a logistic regression on the target data with $LP_{target}$ as an offset variable (the regression coefficient is constrained to be 1).

#### 2.3.3 Model Recalibration: Logistic Calibration

Another method of discrete model updating that uses the new available data to update the model is Recalibration by Logistic Calibration, which intends to correct both the intercept and the overall calibration slope. We implement this method in two variations as follows:

1. **Develop CPM on all data and update on target only**: In this variation, we first develop a logistic regression model on all the development data set (both ancillary and target), then we apply the model to the target data to obtain the linear predictor ($LP_{target}$) for all individuals. Next, we update the model by fitting a logistic regression on the target data with $LP_{target}$ as the only variable.
2. **Develop CPM on ancillary and update on target only**: As an alternative, we first develop a logistic regression model on the ancillary data only, then we apply the model to the target data to obtain the linear predictor ($LP_{target}$) for all individuals. Next, we update the model by fitting a logistic regression on the target data with $LP_{target}$ as the only variable.

#### 2.3.4 Membership‐based recalibration Method: Develop on Ancillary and Target

This method aims to develop a weighted CPM by giving individuals from the ancillary data high weights when they are relevant to target and low weights when they are less relevant to target (Elayan et al. 2025). The weights are defined based on the relatedness of the individual samples from the ancillary data compared with the target data using a probabilistic similarity metric called the “membership model” that is based on the propensity score, thus adjusting the distribution of the ancillary data to correct for distributional changes. See Supporting Information Section S2 for more details on this method. We implement this method in two variations as follows:

1. **Recalibration:** We estimate the membership model in the weighting scheme given the observed values from a set of predictor variables $X_{i}=\left(X_{i1},\text{…},X_{iK}\right)$ or $X_{i}^{+}=\left(X_{i1},\text{…},X_{iK},U_{i}\right)$ only, where K denotes the number of measured predictor variables and U is the unmeasured predictor variable (see Section 2.3.5 below for more details on weighting scheme misspecification). Then after developing the weighted model, we adjust the calibration in the large (CITL) of the weighted model through adding a dummy variable for the target membership R.
2. **Including the outcome** Y **in the weighting scheme:** As an alternative to Recalibration, we estimate the membership model in the weighting scheme given the observed values from a set of predictor variables $X_{i}$ including the outcome Y as predictor, where $X_{i}^{+}=\left(X_{i1},\text{…},X_{iK},Y_{i}\right)$ or $X_{i}^{+}=\left(X_{i1},\text{…},X_{iK},U_{i},Y_{i}\right)$, without adding a dummy variable in the developed weighted model afterwards.

#### 2.3.5 Misspecification of Weighting Schemes and CPMs

We introduce prediction model misspecification, as one would expect to see in real‐world applications, by excluding the main effect of the unmeasured predictor variable U from all models while it remains part of the data generation process. Furthermore, we explore correctly specified and misspecified weighting schemes Membership‐based recalibration method. The correctly specified weighting schemes include the unmeasured predictor variable U, while the misspecified weighting schemes exclude the unmeasured predictor variable U. Table 2 lists all the options followed for the developed models.

**TABLE 2:** List of modeling options.

Model original name	Reference name	Correct/Mis‐specified weighting scheme	Misspecified CPM	Develop on all data	Develop on target only
Develop CPM on target only	Target‐only	—	✓	✗	✓
Recalibration—Intercept only: Develop CPM on all data and update on target	Intercept Recalibration‐all data	—	✓	✓	✗
Recalibration—Intercept only: Develop CPM on ancillary and update on target	Intercept Recalibration‐ancillary only	—	✓	✓	✗
Recalibration—Logistic calibration: Develop CPM on all data and update on target	Logistic Recalibration‐all data	—	✓	✓	✗
Recalibration—Logistic calibration: Develop CPM on ancillary and update on target	Logistic Recalibration‐ancillary only	—	✓	✓	✗
Membership‐based recalibration method: Recalibration	Membership‐based recalibration	✓	✓	✓	✗
Membership‐based recalibration method: Including Y in weighting scheme	Membership‐based add‐Y	✓	✓	✓	✗

### 2.4 Validation and Performance Metrics

We compare each method's ability to estimate the predicted risk, on a very large validation datavset (100,000), drawn from the same data‐generating mechanism as the target population, using the following metrics of predictive performance, covering calibration, discrimination, and prediction stability metrics. **Calibration** was assessed using both calibration‐in‐the‐large and calibration slope slope as summaries of mean/weak calibration (Van Calster et al. 2016; Van Calster et al. 2019). **Calibration‐in‐the‐large (CITL)** was obtained by intercept estimate of each developed model fitted to the observed outcome with fixing linear predictor as an offset variable. **Calibration Slope (CSLOPE)** was obtained by regression coefficient estimate of the linear predictor from a each developed model fitted to the observed outcome with the linear predictor as the only variable. Discrimination was quantified using the area under the receiver operating characteristic curve (AUC). **Brier Score** was estimated to measure the of overall performance, equivalent to the mean squared error of predicted probabilities. Furthermore, **Calibration plot** was used to compare the performance which plots the calibration curve of the observed probabilities against predicted probabilities (moderate calibration). When predicted probabilities fall below the curve, it indicates overestimation, whereas probabilities above the curve indicate underestimation. **Instability plots** by Riley and Collins (2023) were also used to compare the performance as follows:

1. We examine the instability (variability) in the calibration curves of each developed model when assessed on the validation data set. Each scenario was repeated 200 times, with new data generation and model development in each iteration. The calibration curves, representing estimated versus observed risks from these 200 iterations, are overlaid together on the calibration instability plot. The median calibration curve across iterations and its 95% empirical percentile bands (2.5th–97.5th percentiles) are also displayed. A wider spread among the calibration curves indicates a higher level of instability.
2. We examine the variability in the model's mean estimated risk (predicted probability) by plotting the distribution of the mean estimated risk over 200 iterations within each scenario. The mean estimated risk refers to the average predicted probability across all individuals in the validation data set for each iteration. The broader the 95% empirical range of a model's mean estimated risks, the greater the instability in the model's mean risk estimates, which is likely to result in miscalibration between the mean estimated and mean observed risk
3. We examine the variability and range of individual‐level predictions from 200 iterations for each model. The individual stability scatter plots show the predicted values (*y*‐axis) for the same nine randomly selected individuals over 200 iterations within each scenario. The more dissimilar the predicted probabilities, the more unreliable the model's predictions. (Riley and Collins 2023) define “reliable” as different example models (i.e., models from different iterations) producing similar estimated risks for an individual.

### 2.5 Software

All analyses were performed using R version 4.5.0. Riley et al.'s (2019) minimum sample size criteria was calculated using “pmsampsize” package and “pROC” package was used to calculate AUC, developed by Robin et al. (2011). Code to replicate the simulation can be found in the following Zenodo repository: (https://zenodo.org/records/21454019)

## 3 Simulation Results

In this section, we focus on a subset of the simulated scenarios for discussion. All the reported results are for the outcome‐generation model with 15 predictors, proportion split between the ancillary and target sets is “**75% ancillary**: **25% target,**” and the total sample size is four times the minimum sample size ($4\times N_{min}$), which is sufficient overall with the target data set at the minimum required sample size. Moreover, the reported Membership‐based recalibration model results are for a misspecified weighting scheme. In Section 3.6, we report the results of the other factors such as outcome‐generation models with 5 and 4 predictors, different proportion split between the ancillary and target sets, different development sample sizes, and the Membership‐based recalibration method for correctly specified weighting scheme.

All figures in this section display the median performance metrics (AUC, CITL, calibration slope, and Brier Score) across 200 iterations. Each model is displayed on a separate horizontal line on the *y*‐axis. Symbols (e.g., circles, squares, triangles) and error bars represent the median and 95% confidence interval (CI) across 200 iterations for the corresponding model on that line with the following color scheme: Target‐only (pink), Logistic Recalibration – all data (blue), Logistic Recalibration – ancillary only (cyan), Intercept Recalibration – all data (green), Intercept Recalibration – ancillary only (brown), and Membership‐based recalibration (red).

### 3.1 No Shift

**FIGURE 2:**
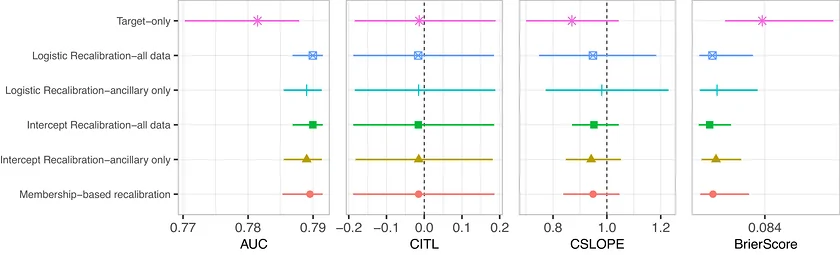
Performance metrics for No Shift. Symbols and error bars correspond to the median and 95% confidence interval for each model on the *y*‐axis across 200 iterations.

Figure 2 shows the median performance metrics (AUC, CITL, calibration slope, and Brier score) across 200 iterations, with 95% CIs indicated as error bars, for the No Shift scenario. The Target‐only model showed the lowest median AUC with 95% CI of 0.781 (0.770,0.788) and lowest median calibration slope with 95% CI of 0.870 (0.700,1.044) across iterations, compared to all other methods. In terms of calibration slope performance, Logistic Recalibration‐all data and Logistic Recalibration‐ancillary only models exhibited greater variability with a wider CI of 0.948 (0.748,1.184) and 0.981(0.773,1.229), respectively. In contrast, the Membership‐based recalibration model, the Intercept Recalibration‐all data, and the Intercept Recalibration‐ancillary only models had almost similar calibration slope results. All models demonstrated good calibration‐in‐the‐large, with a median CITL of 0.0 (−0.2, 0.2) across iterations. Regarding Brier Score, all models achieved similar results, except the Target‐only model which showed wider median CI and a slightly higher median Brier Score of 0.084 (0.083,0.086).

**FIGURE 3:**
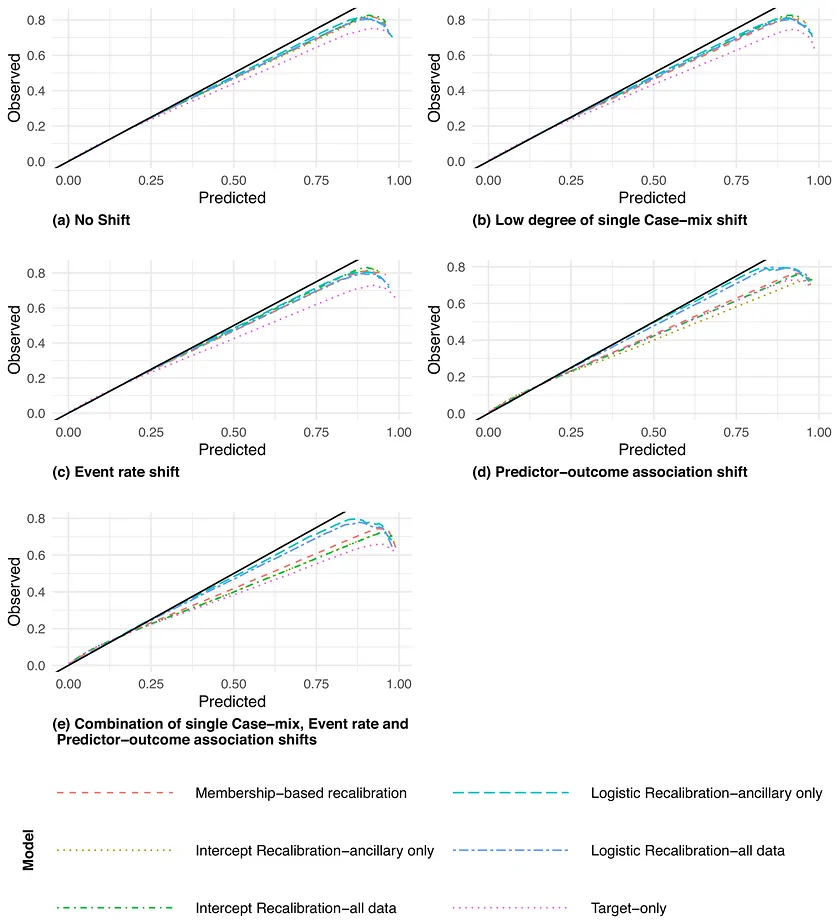
Mean calibration instability curves across 200 iterations. *Note:* The ideal line is shown in solid black.

For calibration plots, as shown in Figure 3a, almost all models were generally well calibrated on average, except for the Target‐only model. However, all models experienced overestimation at higher predicted risks. Also, both Logistic Recalibration models and Target‐only model had wider spread in calibration curves at higher predicted risk compared to other models, as shown in Figure S8 in Supporting Information, indicating greater instability concern and thus potential for miscalibration in new data. Moreover, the Target‐only model showed greater variability in estimated risk across 200 iterations for all individuals compared to other models, as illustrated in Figure S9 in Supporting Information. This higher variability indicates that the predicted probabilities from this model are less stable. Also as shown in Figure S7a in Supporting Information, all models had 95% of mean estimated risks between 0.09 and 0.12 across 200 iterations.

### 3.2 Case‐Mix Shift

**FIGURE 4:**
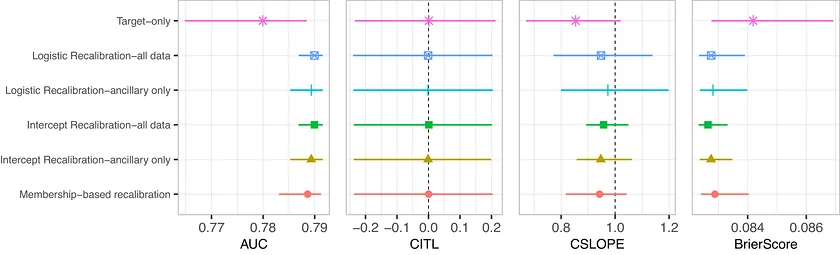
Performance metrics for low degree of single Case‐mix shift. Symbols and error bars correspond to the median and 95% confidence interval for each model on the *y*‐axis across 200 iterations.

Under the low degree of Case‐mix shift in mean, ($\Delta \mu_{X_{1}}=0.8$), as illustrated in Figure 4, model performances remained largely consistent with the No Shift scenario, with some exceptions. Calibration slope median across iterations showed slight decrease median value, particularly for Logistic Recalibration‐ancillary only model had and Target‐only model compared to the No Shift case. Also, CITL medians across iterations remained stable across all models, maintaining a median of 0.0 (−0.2, 0.2). The Brier Scores were nearly identical to the No Shift scenario, though the Target‐only model retained the highest median across iterations and the widest CI of 0.084 (0.083,0.087). Regarding calibration instability, trends from the No Shift case persisted under Case‐mix shift, as shown in Figure 3b.

This trend remains consistent even when the degree of Case‐mix shift is increased. As the deviation from the ideal calibration increases across all models, or as AUC medians decrease, the relative ranking of the models stays the same. On the other hand, the high degree of Case‐mix shift in mean ($\Delta \mu_{X_{1}}=4$) was a unique scenario compared to other combinations and degrees of Case‐mix shifts. The Membership‐based recalibration model down‐weighted all ancillary dataset samples to 0, as propensity score strictly did not allow for extrapolation, resulting in a performance similar to the Target‐only model. In terms of AUC and CSLOPE, these two models underperformed, with lower medians and wider CIs compared to the other models. See Supporting Information Figure S1 for the results of this scenario.

### 3.3 Event Rate Shift

**FIGURE 5:**
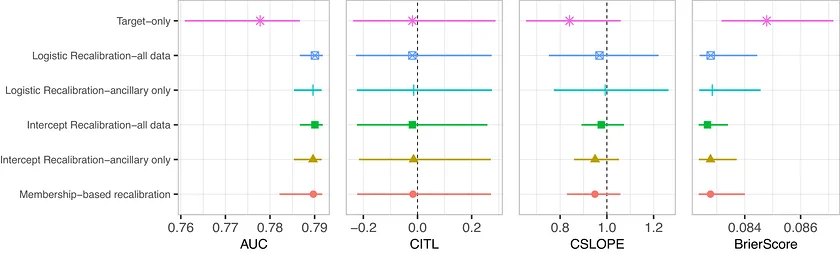
Performance metrics for Event rate shift. Symbols and error bars correspond to the median and 95% confidence interval for each model on the *y*‐axis across 200 iterations.

For the Event rate shift, $\Delta \beta_{0}=0.9$, as shown in Figure 5, models' performance remained similar to the No Shift case, with slight variations. The Target‐only model showed lower median AUC across 200 iterations of 0.778 (0.761,0.787), indicating reduced discrimination and lower calibration slope median of 0.840 (0.654,1.060), compared to the No Shift case and other models. While CITL values remained almost stable, the Target‐only model had a slightly higher median Brier Score across iterations 0.085 (0.083,0.087). For calibration instability, as shown in Figure 3c, trends were consistent with the No Shift case. Most models were well calibrated, but the Target‐only model showed poorer calibration, with its median calibration line farther from the ideal at higher predicted risks.

Furthermore, across the 200 simulation iterations, paired Wilcoxon signed‐rank tests were conducted to compare the AUC of each CPM method against the Membership‐based recalibration (Barbini et al. 2007). As shown in Table S12 (Supporting Information), the Target‐only approach had significantly lower discrimination performance than the Membership‐based recalibration (ΔAUC = −0.012, *p*‐value < 0.001). For the recalibration methods, differences in AUC were negligible (|ΔAUC|≤0.0004). Although the intercept and logistic recalibration approaches using all available data showed statistically significant differences (both ΔAUC = 0.0004, *p*‐value < 0.001), the magnitude of these differences was extremely small. In contrast, the corresponding ancillary‐only recalibration approaches showed no evidence of a difference in AUC (both ΔAUC = −0.0001, *p*‐value = 0.291). Overall, these findings suggest that the Membership‐based recalibration achieved discrimination performance that was practically comparable to the alternative recalibration methods.

### 3.4 Predictor–Outcome Association Shift

**FIGURE 6:**
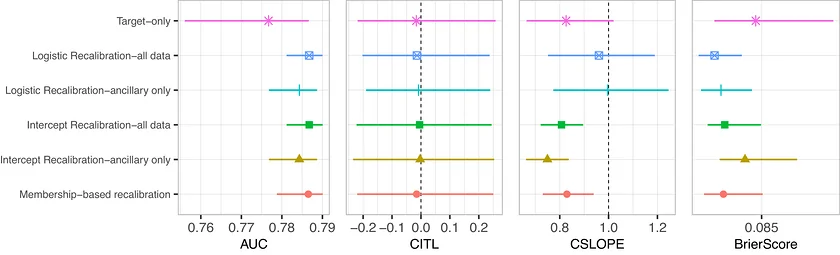
Performance metrics for Predictor–Outcome association shift ($\Delta \beta_{1}=0.4$). Symbols and error bars correspond to the median and 95% confidence interval for each model on the *y*‐axis across 200 iterations.

In the Predictor–Outcome association shift scenario, $\Delta \beta_{1}=0.4$, as illustrated in Figure 6, a notable difference is observed in the AUC values, which generally decrease when compared to the No Shift models. The Target‐only model experienced the greatest reduction in median AUC, with a value of 0.777 (0.756,0.787). The calibration slope median across iterations also showed a notable change, specifically the Intercept Recalibration‐all data and Intercept Recalibration‐ancillary only models showed a more substantial decrease in median calibration slope of 0.807 (0.722,0.896) and 0.749 (0.662,0.837), respectively, with relatively narrow CIs compared to all models. The CITL median across iterations remained close to zero for all models, indicating minimal changes in calibration. Additionally, Brier Scores increased across all models, reflecting the decline in all models' performance. Notably, the Target‐only model showed a higher median of 0.085 (0.083,0.088).

For calibration instability plots, as shown in Figure 3d, both Logistic Recalibration models maintained good overall calibration across the predicted risk range, though their calibration curves had a wider spread at higher predicted risks, indicating greater instability and a higher potential for miscalibration in new data. In contrast, the other models had poorer calibration on average. Additionally, while the Intercept Recalibration‐ancillary only model had the narrowest spread in its calibration curves, it was the most miscalibrated model on average.

### 3.5 Combination of Single Case‐Mix, Event Rate, and Predictor–Outcome Association Shifts

**FIGURE 7:**
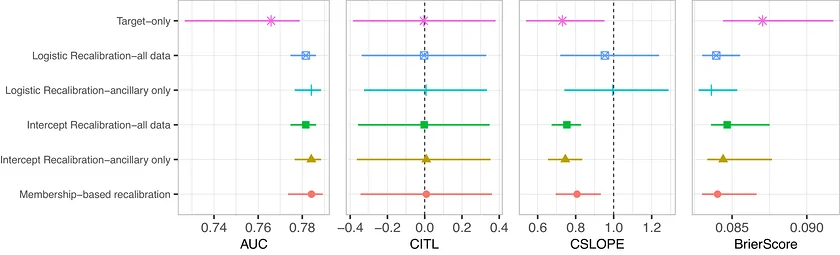
Performance metrics for combination of single Case‐mix, Event rate and Predictor–Outcome association shifts. Symbols and error bars correspond to the median and 95% confidence interval for each model on the *y*‐axis across 200 iterations.

For the combination of single Case‐mix, Event rate, and Predictor–Outcome association shifts ($\Delta \mu_{X_{1}}=0.8$, $\Delta \beta_{1}=0.4$, and $\Delta \beta_{0}=0.9$), as shown in Figure 7, the Target‐only model had the worst performance across AUC, calibration slope, and Brier Score median over 200 iterations compared to No Shift, with the lowest AUC median at 0.766 (0.727,0.779), the calibration slope median dropped to 0.730 (0.539,0.952), and Brier Score median increased to 0.087 (0.084,0.092). Also, both Logistic Recalibration models showed greater variability, with the Logistic Recalibration‐all data model calibration slope median widening to 0.954 (0.719,1.239), while the the calibration slope CI for the ancillary‐only model expanded to 0.995 (0.740,1.289). In contrast, both Intercept Recalibration models experienced a decline in calibration slope, with the all data model dropping to 0.754 (0.673,0.829) and the ancillary‐only model to 0.745 (0.655,0.834), indicating weaker calibration compared to No Shift. In addition, the Membership‐based recalibration model showed a decline in calibration slope median, falling to 0.807 (0.694,0.932), though it remained higher than both Intercept Recalibration models. The CITL median remained near zero, but the CIs expand substantially, particularly for the Intercept Recalibration‐ancillary only model at 0.0 (−0.4, 0.4), reflecting increased uncertainty in calibration.

This scenario had almost similar performance for all models compared to predictor–outcome association shift scenario in terms of calibration instability plot, as shown in Figure 3e. Both Logistic Recalibration models maintained good overall calibration across the predicted risk range, though their calibration curves had a wider spread at higher predicted risks. In contrast, all other models had poorer calibration on average, particularly the Target‐only model. Additionally, while the Intercept Recalibration‐ancillary only model had the narrowest spread in its calibration curves, it was the most miscalibrated model on average.

This trend remains consistent even when combining multiple Case‐mix shifts with Event rate shift and Predictor–Outcome association shift. The overall performance of all models decreases, with more deviation from the ideal calibration, lower AUC medians or larger CIs, the relative ranking of the models stay the same.

### 3.6 Impact of Other Factors

#### 3.6.1 Weights Misspecification and Adding Outcome to the Weighting Scheme

Across all the simulated scenarios, and as shown in Figure S2 in Supporting Information, the Membership‐based recalibration models with correctly specified and misspecified weighting schemes perform similarly. This suggest that a correct specification of the weighting scheme does not significantly influence the model's performance, as it can perform identically or almost equally well even with a misspecified weighting scheme. Moreover in the cases of Event rate shift or Predictor–Outcome association shift, the Membership‐based add‐Y model performed worse than Membership‐based recalibration model, showing lower median over 200 iterations or wider CI across all performance metrics. Specifically, adding the outcome in the weighting scheme instead of recalibration resulted in miscalibration‐in‐the‐large with a value below 0.

#### 3.6.2 Proportion Split of Ancillary and Target Sets

Across all the simulated scenarios, and as shown in Figure S3 in Supporting Information, the models showed a slight increase in terms of AUC and calibration slope medians across iterations as the proportion of the target data increased. Notably, the Target‐only model showed the greatest improvement as more data from the target data set were included, reflected in a higher AUC median with narrower CIs, and a calibration slope median closer to 1, also with reduced variability. However, an exception was observed in both the Logistic and Intercept Recalibration–ancillary only models. Increasing the target data while reducing the ancillary data led to a lower AUC median with wider CIs for both models, while the Intercept Recalibration–ancillary only model also exhibited a lower calibration slope median with wider CI.

#### 3.6.3 Development Sample Size

Across all scenarios, and as illustrated in Figure S4 in Supporting Information, all models showed a decrease in performance as the total development sample size decreased. Particularly, the Target‐only model showed the worst impact on its performance with a lower AUC and calibration slope medians compared to other models.

To further assess how ancillary data contributed to model performance across different sample sizes, we computed per‐metric differences (Δ) between each CPM method and the Target‐only model under the No Shift scenario (Figure S19 in the Supporting Information). For each simulation iteration and metric (AUC, Brier Score, calibration slope, and calibration intercept), Δ was defined as the difference in performance relative to the Target‐only model within the same iteration and sample size. The mean Δ across 200 simulation iterations was then calculated to summarize the average relative performance of each method. Positive Δ values indicate improvement over the Target‐only model, while negative values reflect degradation. These Δ values revealed a consistent diminishing‐returns pattern: The benefit of incorporating ancillary data was greatest at the smallest development sample size ($N_{min}$) and progressively diminished as the total development sample size increased ($2\times N_{min}$ and $4\times N_{min}$). For example, at $N_{min}$, all CPM methods improved discrimination by approximately ΔAUC = 0.03–0.04 and calibration slope by 0.24–0.40, while reducing the Brier score by around 0.006–0.007. At $4\times N_{min}$, these gains decreased to ΔAUC = 0.008–0.009 and ΔCSLOPE = 0.07–0.12. This trend indicates that ancillary data provide the greatest benefit when target data are limited, with diminishing gains as the total development sample size increases.

#### 3.6.4 Number of Predictor Variables in Outcome‐Generation Model

Across all simulated scenarios, as illustrated in Figure S5 in Supporting Information for example, prediction models with 4 and 5 predictors demonstrated lower AUC medians and wider CIs across 200 iterations, as well as calibration slope medians that were closer to 1 with wider CIs, compared to the model with 15 predictors. Furthermore, for combined shifts scenario as illustrated in Figure S6 in Supporting Information, the proposed Membership‐based recalibration demonstrated better calibration slope median across 200 iterations as the number of predictors decreased, compared to the Intercept Recalibration models. Additionally, it exhibited narrower confidence intervals compared to both Logistic Recalibration and Target‐only models.

## 4 Application to SWEDEHEART Registry

To illustrate the studied CPM development and updating methods throughout the simulation, we applied the methods to the “SWEDEHEART” registry, a real‐world data set. The Swedish Web‐System for Enhancement and Development of Evidence‐Based Care in Heart Disease Evaluated According to Recommended Therapies (SWEDEHEART) is a national quality registry including patients admitted to a coronary care unit or other specialized facility with a suspected or definite acute coronary syndrome (ACS) (Jernberg et al. 2010). The cross‐linked data from SWEDEHEART, the Swedish Cardiopulmonary Resuscitation Registry, and the Swedish Pacemaker and Implantable Cardioverter‐Defibrillator (ICD) Registry were used in this study. Patients, registered in SWEDEHEART between 2009 and 2017, with MI, who had undergone coronary angiography and were discharged alive without a prior ICD were included (Faxén et al. 2020). Seven variables including; Age, Sex, Diabetes, Atrial fibrillation/flutter (AF), Body mass index (BMI), Left ventricular ejection fraction (LVEF), and Estimated glomerular filtration rate (eGFR) were selected for inclusion as predictor variables to predict the out‐of‐hospital cardiac arrest (OHCA) within 90 days as an outcome (Faxén et al. 2020).

The registry contains 90 distinct healthcare centers. Our goal is to develop a prediction model to be used in Lund healthcare center that is part of the Skåne University Hospital in Lund, Sweden. This example aims to enhance the utility of the local or regional data (target data) by integrating ancillary (national) data from the remaining centers when developing a CPM. Therefore, we compared the predictive performance of CPMs developed using all the discussed methods using data from Lund as the target data set (6718 patients) and the data from the rest of centers—except Lund—as the ancillary data set (159,903 patients). While this example focuses on data across geographical locations, the underlying principles are equally applicable to data over time. For the updating methods, the models were either developed on all data including Lund or on the ancillary data only without data from Lund, then the models were updated to Lund data. For the Membership‐based recalibration method, the model was developed using all the available data with the same set of predictor variables used to calculate the weights. While for the target‐only method, the model was developed on data from Lund only.

Both apparent and bootstrap internal validation results of predictive performance (AUC, calibration slope and calibration‐in‐the‐large, and Brier Score) were reported from the target set. The adjusted optimism for each performance metric was estimated to provide an estimate of predictive performance after removing in‐sample optimism as developing a model in a data set and then testing the model in that same data (without adjustment) will lead to overly optimistic performance estimates. To estimate optimism, we used 200 bootstrap resamples of the development data. For each bootstrap iteration, a model was fitted to the resampled (bootstrap) data set, and its performance was evaluated both within the target data bootstrap sample only (the bootstrap apparent performance) and in the original target sample (the bootstrap test performance). The difference between these two values represents the optimism for that bootstrap iteration. The mean optimism across all bootstrap iterations were then subtracted from the development apparent performance to obtain the optimism‐corrected estimate (Steyerberg et al. 2001; Harrell 2016). Please refer to the Supporting Information for illustration of estimating the optimism‐adjusted performance metric Figure S18.

### 4.1 SWEDEHEART Example Results

**FIGURE 8:**
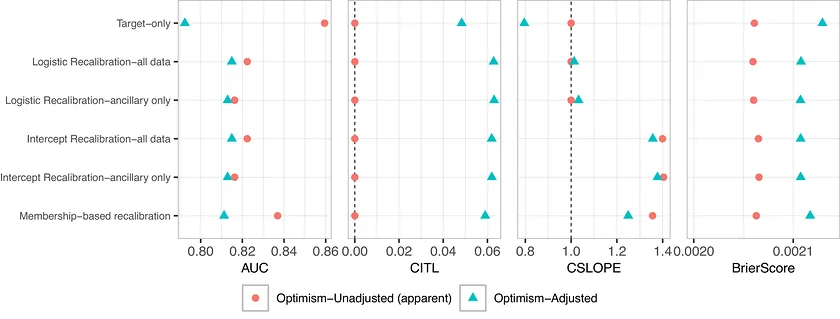
Performance metrics of SWEDEHEART case study.

**FIGURE 9:**
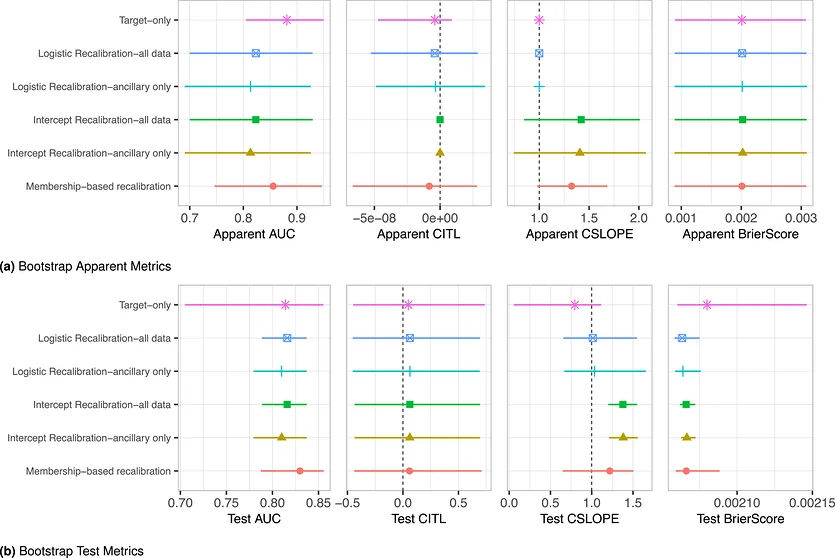
Bootstrap performance metrics' confidence intervals of SWEDEHEART case study. Symbols and error bars correspond to the median and 95% confidence interval for each model on the *y*‐axis across 200 iterations.

For the empirical data, the Target‐only model had the lowest optimism‐adjusted AUC, calibration slope, and Brier Score compared to all other methods (Figure 8). Additionally, it had the widest CIs across 200 iterations in all bootstrap test performance metrics, as shown in Figure 9. Both Logistic Recalibration models had an optimism‐adjusted calibration slope almost equal to 1, though they displayed wide CIs for the bootstrap calibration slope over 200 iterations. Also, the Membership‐based recalibration model had a slightly closer optimism‐adjusted calibration slope to 1 compared to both Intercept Recalibration models. However, the Membership‐based recalibration model had wider CIs for the bootstrap test calibration slope across 200 iterations, whereas the Intercept Recalibration models showed wider CIs for the bootstrap apparent calibration slope.

## 5 Discussion

In this study, we compared the predictive performance of different CPM methods for incorporating the ancillary data when developing CPMs under a variety of simulated scenarios and in a real‐world example using SWEDEHEART registry data set. We found that in all scenarios, the Target‐only model underperformed in terms of AUC, calibration slope, and Brier Score, except in Predictor–Outcome association shift only scenario, where it had almost similar calibration slope median across iterations compared to the Membership‐based recalibration model, but with slightly wider CI. Moreover, across all scenarios, both Logistic Recalibration models showed high variability in calibration slope with wider CIs, compared to all other models. In addition, in presence of the Predictor–Outcome association shift, both Intercept Recalibration models underperformed other models in calibration slope median across 200 iterations, though they showed a very narrow CI. The Membership‐based recalibration method showed comparable performance to Intercept recalibration methods under Case‐mix, except in scenarios with a high degree of shift or event rate shift. However, it had higher calibration slope median in the presence of predictor–outcome association shift, and in the presence of different types of shifts simultaneously compared to Intercept Recalibration methods, offering a more stable calibration. We have provided a concise list of key take home messages in Table 3. Moreover, the real‐world example results were consistent with the simulation findings: the Target‐only model consistently underperformed compared to all other methods. The Logistic Recalibration models achieved CSLOPE medians close to 1, but with high variability. Both the Membership‐based recalibration and Intercept Recalibration models demonstrated comparable calibration and discrimination metrics, generally outperforming the Target‐only model and showing slightly narrower confidence intervals than the Logistic Recalibration models in test CSLOPE.

**TABLE 3:** Table of key take home messages for enhancing CPM performance when incorporating ancillary data.

Key take home message
1. Including the ancillary data improves CPM performance, particularly with small target sample sizes.
2. Intercept Recalibration methods generally improve performance, but they perform worse in calibration slope under predictor–outcome association shift.
3. Be cautious with Logistic Recalibration due to its greater variability in calibration slopes and more instability in calibration curves.
5. The Membership‐based recalibration method showed consistent performance across varied scenarios. This method could be a reliable alternative, particularly in practical settings where the presence and type of data distribution shift is often unknown.

Our study also illustrates a tipping‐point behaviour in the benefit of ancillary data. By calculating per metric differences between each CPM method and the Target‐only model across 200 simulation iterations, we observed that the largest gains in discrimination and calibration occurred at the smallest development sample size ($N_{min}$) and progressively decreased with larger total sample sizes ($2\times N_{min}$ and $4\times N_{min}$). Notably, with the 25%–75% split between target and ancillary data, the target subset at $4\times N_{min}$ corresponds to the minimum required sample size for independent model development based on Riley et al. (2019) criterion. This indicates that the benefit of incorporating ancillary data is most pronounced when the target data alone are limited, and diminishes once the target data reach the minimum threshold required for reliable model estimation. This aligns with findings by Rajput et al. (2023), who reported that the benefit of adding additional data diminishes as the sample size grows, with model performance and effect size stabilizing once a critical sample size is reached. Similarly, Pate et al. (2020) demonstrated that while increasing sample size generally improves model calibration, discrimination, and coefficient stability—particularly at smaller sample sizes—they reported that further increases in sample size yield negligible improvements in model performance once the model coefficients have stabilized.

While our simulation study compares CPM methods to enhance predictive performance for the target population by integrating the ancillary data into a broader development data set while accounting for heterogeneity in it, other studies have focused on comparing updating methods to integrate new data from the deployment phase of a CPM. Davis et al. (2019) developed a testing procedure that recommend regression updating methods to maintain performance of CPM over time by incorporating new data without losing relevant information from the original model (incorporate new data from deployment phase for existing model). More recently, Davis et al. (2022) used the same testing procedure in a surveillance‐based approach that monitors the performance of the model for postcardiac catheterization acute kidney injury. This method was used to guide the timing and method of updating the model in comparison with the originally developed model, and the annually retrained model, using more recent routinely collected data over the 6 years after model development. The surveillance‐based approach provided superior calibration through retraining the model once and recalibrating it twice while the original model overpredicted risk of acute kidney injury. Similarly, Janssen et al. (2008) compared different updating methods to incorporate new patients data for acute severe postoperative pain into an existing model. The results showed that updating methods can be applied to adjust the poor performance of the model on new patients, rather than to develop a new model. Also, simple recalibration methods improved the calibration to a similar extent as revision methods. Ivanov et al. (1999) also compared recalibration and developing new model under temporal shifts in case mix in new data of isolated CABG series to using existing model developed on a data set that combined ischemic and valvular heart disease. The authors found that both the recalibrated model and the newly developed model performed slightly better than using the original model. Moreover, Siregar et al. (2019) recommended using Updating methods as an efficient alternative to using an existing model. Their applied example on assessing the predictive performance of the EuroSCORE II in the Netherlands Heart Registry data set showed an overestimation of the risk of mortality. However, Logistic Recalibration significantly improved model performance without the risk of overfitting, outperforming all other updating methods. Strobl et al. (2015) also proposed similar work, focusing on updating an existing clinical risk calculator for prostate cancer. Their findings showed that recalibration significantly improved calibration over using the existing model in most validation cohorts.

A special case of the problem was discussed by Booth et al. (2023). The authors introduced temporal recalibration to improve prognostic model calibration by adjusting for survival trends over time. Similar to the Intercept Recalibration using all data method for logistic regression, this approach estimates predictor effects from the full data set while re‐estimating the baseline with recent data and keeping the predictor effects fixed. The approach was applied to competing risk settings by recalibrating cause‐specific hazard models for colon cancer and other causes. Comparisons were made with the standard approach, which does not account for improvements over time, and period analysis. The results showed that the standard approach overestimated mortality risk, while temporal recalibration improved calibration, particularly in 10‐year predictions. Debray et al. (2012) explored a similar problem, focusing on how previously published prediction models can be used in developing new prediction models. Unlike our study, which investigates incorporating the ancillary data sets into the development of a CPM, Debray et al. examined the integration of published models and individual patient data (IPD) that share similar predictors, assuming identical model formulations for the published models. All their assessed aggregation approaches involved recalibrating the model with new data to maintain calibration. Their findings indicate that incorporating previously published models with new data from target population can enhance the development of prediction models, especially when limited participant data are available. Our work is also related to the emerging literature on prediction under interventions (van Geloven et al. 2025). Authors discussed the challenges that arise when developing prediction models using data that include patients who have already received the treatment or intervention that the model is intended to inform. In such situations, the estimated risks may reflect a mixture of treated and untreated outcomes, which can lead to biased estimates of the underlying prognosis and compromise the model's use for future decision‐making. Although our study did not explicitly account for treatment effects in the simulated data, similar challenges may occur when ancillary data sets differ from the target population in terms of treatment prevalence or clinical practices. This issue is particularly relevant when integrating ancillary data, as differences in treatment exposure across data sets can amplify heterogeneity and introduce additional bias in model estimation. In this context, our findings complement those of van Geloven et al. (2025) by focusing on the impact of heterogeneity in data distributions—such as Case‐mix, Event rate, and Predictor–Outcome associations—on model development and performance, rather than on causal bias introduced by prior interventions. In addition, our study is related to the transfer learning literature, although the objectives differ. Among the methods considered, importance weighting using propensity scores is commonly used in transfer learning to address case‐mix or covariate shift (Pan and Yang 2010). However, transfer learning typically focuses on adapting or transferring a model trained in a source domain for application in a target domain, whereas our focus is on incorporating ancillary data during model development to improve prediction for the target population.

The main strength of this work is that we performed a simulation study under a range of scenarios and consider multiple performance measures, thereby allowing a comprehensive and systematic examination of modeling approaches. To our knowledge, we are the first to compare the performance of regression updating and weighting methods for incorporating the ancillary data when developing CPMs while correcting for heterogeneity between target and the ancillary data sets caused by data distribution shift. A limitation of this study is the use of a single‐source ancillary data set in the simulation study to illustrate the proposed methods, together with an emphasis on sudden shifts between the ancillary and target data sets. We partially address the single‐source limitation by including a real‐world application based on a multi‐source ancillary data set drawn from different regions. However, the generalizability of the results to settings with greater heterogeneity remains uncertain. In Sweden both patient populations and post‐MI care pathways are relatively homogeneous across centers, partly due to national guidelines and coordinated clinical practice within the SWEDEHEART Registry network. Future research should further investigate the impact of multiple ancillary data sets under controlled simulation settings, explore scenarios involving gradual shifts between ancillary and target data, and apply the proposed methods to more heterogeneous real‐world data sets, in line with phase‐three methodological research development (Heinze et al. 2022).

## 6 Conclusion

We conducted an extensive simulation study, complemented by a real‐world data illustrative example, and found that incorporating ancillary data generally improved model performance compared with using target data alone, particularly when the target sample size was small. However, it is crucial to account for heterogeneity between ancillary and target data sets by selecting appropriate methods. Both Logistic and Intercept Recalibration generally improved performance over Target‐only model. Logistic Recalibration showed greater variability in calibration slopes and more instability in calibration curves. Intercept Recalibration performed well in many situations but may be less robust under predictor–outcome association shifts. The Membership‐based recalibration method showed consistent results with improved performance compared to other methods in many scenarios, particularly under predictor–outcome association shift. Overall, Membership‐based Recalibration can be recommended. The choice of method should be guided by the expected type of shift, target sample size, and the relative importance of stability versus point estimate performance, especially given that in practical settings the presence and type of distributional shifts are often unknown.

## Author Contributions

We confirm that the manuscript has been read and approved by all named authors. H.E, D.A.J, G.P.M, and M.S conceived and designed the study. H.E performed the analysis and wrote the main manuscript text. D.A.J, G.P.M, M.S, F.B, J.F, and J.A reviewed/edited the manuscript.

## Funding

The authors have nothing to report.

## Ethics Statement

Ethical approval for the SWEDEHEART data analysis is managed through the registry's data provision process, with all data anonymized prior to use.

## Conflicts of Interest

The authors declare no conflicts of interest.

## Open Research Badges

This article has earned an Open Data badge for making publicly available the digitally‐shareable data necessary to reproduce the reported results. The data is available in the Supporting Information section.

This article has earned an open data badge “**Reproducible Research**” for making publicly available the code necessary to reproduce the reported results. The results reported in this article were reproduced partially due confidentiality issues.

## Supporting information


**Supporting File 1:** bimj70166‐sup‐0001‐SuppMat.pdf.

**Supporting File 2:** bimj70166‐sup‐0002‐Data.zip.

## Data Availability

The data sets analyzed during the current study are not publicly available due to the required data sharing agreements and considerations. Access to data available for research by application to the SWEDEHEART steering group. All statistical code for the analysis is available through open‐source repositories (Zenodo repository) for full transparency and reproducibility.

## Endnotes

1 These values were selected based on prior studies of cardiovascular data set (Jernberg et al. 2010; Faxén et al. 2020)

2 These values were selected based on prior studies of EuroSCORE model (Hickey et al. 2013).
