# Supplementary material for: Comparison of Methods for Incorporating Related Data When Developing Clinical Prediction Models: A Simulation Study
Source: Biom J. 2026 Aug 27;68(5):e70166. doi: 10.1002/bimj.70166 (PMC13520826; doi:10.1002/bimj.70166)
Supplement: Supplementary file 1 [file BIMJ-68-e70166-s002.pdf]

S1 Data-generating Mechanism

Table S1: Target population covariance matrix

	X_1	X_2	X_3	X_4	X_5	X_6	X_7	X_8	X_9	X_{10}	X_{11}	X_{12}	X_{13}	X_{14}	U
X_1	1	0.4	0.3	0	0	-0.4	-0.2	0	0	0.4	0	0	0	0	0.4
X_2		1	0.6	-0.3	0	0	0	0	0	0.5	0	0	0	0	0.3
X_3			1	0	0.2	0	0	0	0	0	0	0	0	0	0
X_4				1	0	0	0	0	0	0	0	0	0	0	0
X_5					1	0	0	0.1	0	0	0	0	0	0	0
X_6						1	0	0	-0.4	0	0	0.3	0	0	0
X_7							1	0.3	0	0	0.3	0	0	0.45	0
X_8								1	0.5	0	0	0	0	0	-0.3
X_9									1	0	0	0	0.3	0	0
X_{10}										1	0	0	0	0	0.6
X_{11}											1	0.5	0.4	0	0.4
X_{12}												1	0.45	0	0
X_{13}													1	0	0
X_{14}														1	0
U															1

Table S2: Binary predictors prevalence

Predictor	p
X_{10}	0.3
X_{11}	0.4
X_{12}	0.1
X_{13}	0.2
X_{14}	0.4

Table S3: Target population coefficient and shift in coefficient choices for outcome-generation models

Model with 15 predictors	Model with 5 included predictors	Model with 4 included predictors	Shift in coefficient
$\beta_{0,target}$=-3.4	$\beta_{0,target}$=-3.1	$\beta_{0,target}$=-2.5	$\Delta\beta_0$=[0, 0.9]
$\beta_{1,target}$ =0.5	$\beta_{1,target}$ =0.5	$\beta_{1,target}$ =0.5	$\Delta\beta_1$ =[0, 0.4]
$\beta_{2,target}$ =0.3	$\beta_{2,target}$ =0.3	$\beta_{2,target}$ =0.3	$\Delta\beta_2$ =0
$\beta_{3,target}$ = 0.2	$\beta_{3,target}$ =0	$\beta_{3,target}$ = 0.2	$\Delta\beta_3$ =0
$\beta_{4,target}$ =0.1	$\beta_{4,target}$ =0	$\beta_{4,target}$ =0	$\Delta\beta_4$ =0
$\beta_{5,target}$ =-0.02	$\beta_{5,target}$ =0	$\beta_{5,target}$ =0	$\Delta\beta_5$ =0
$\beta_{6,target}$ =0.1	$\beta_{6,target}$ =0	$\beta_{6,target}$ =0	$\Delta\beta_6$ =0
$\beta_{7,target}$ =-0.1	$\beta_{7,target}$ =0	$\beta_{7,target}$ =0	$\Delta\beta_7$ =0
$\beta_{8,target}$ =0.15	$\beta_{8,target}$ =0	$\beta_{8,target}$ =0	$\Delta\beta_8$ =0
$\beta_{9,target}$ =-0.1	$\beta_{9,target}$ =0	$\beta_{9,target}$ =0	$\Delta\beta_9$ =0
$\beta_{10,target}$ =0.7	$\beta_{10,target}$ =0.7	$\beta_{10,target}$ =0	$\Delta\beta_{10}$ =0
$\beta_{11,target}$ =0.8	$\beta_{11,target}$ =0.8	$\beta_{11,target}$ =0	$\Delta\beta_{11}$ =0
$\beta_{12,target}$ =0.5	$\beta_{12,target}$ =0	$\beta_{12,target}$ =0	$\Delta\beta_{12}$ =0
$\beta_{13,target}$ =0.6	$\beta_{13,target}$ =0	$\beta_{13,target}$ =0	$\Delta\beta_{13}$ =0
$\beta_{14,target}$ =0.2	$\beta_{14,target}$ =0	$\beta_{14,target}$ =0	$\Delta\beta_{14}$ =0
$\beta_{U,target}$=0.2	$\beta_{U,target}$=0.2	$\beta_{U,target}$=0.2	$\Delta\beta_U$ =[0, 0.4]

S2 Membership-based recalibration method: Develop on ancillary and target

This method aims to develop a weighted CPM by giving individuals from the ancillary data high weights when they are relevant to the target data and low weights when they are less relevant to the target data [1]. The weights are defined based on the relatedness of the individual samples from the ancillary compared to the target using a probabilistic similarity metric called "membership model" that is based on the propensity score methodology, thus adjusting the distribution of the ancillary to correct for distributional changes.

First, we utilize the membership propensity score (PS), which is defined as the conditional probability of a randomly chosen individual in the study population belonging to the ancillary, given their observed values from a set of predictor variables $\boldsymbol{X}_i = (X_{i1}, ..., X_{iK})$ or $\boldsymbol{X}_i^+ = (X_{i1}, ..., X_{iK}, U_i)$, where K denotes the number of measured predictor variables and U is the unmeasured predictor variable, such that: $PS_i = P(R = 1|\mathbf{X}_i)\, for\, i \in \{1, ..., N_{ancillary}\}$.

We use a single binary logistic regression model (membership model) to estimate PS, where the outcome of the model is 0 for individuals from the target and 1 for individuals from the ancillary [2]. This model therefore provides, for each individual, both $P(R = 1 \mid \mathbf{X}_i)$ (probability of belonging to the ancillary) and $P(R = 0 \mid \mathbf{X}_i) = 1 - PS_i$ (probability of belonging to the target). Then, we produce a weight w_i for an individual i from the ancillary dataset by dividing the probability of belonging to the target by the probability of belonging to the ancillary, both obtained from the same membership model.

To account for differences in sample sizes between the target and ancillary datasets, we scale the weight by the ratio of their sample sizes (i.e., the ancillary sample size divided by the target sample size). This adjustment ensures that each dataset contributes proportionally to the final weights, reflecting the relative sizes of the datasets. The adjusted weight is defined as follows:

$$w_i = min\left(\left(\frac{P(R=0|\mathbf{X}_i)}{P(R=1|\mathbf{X}_i)} \times \frac{N_{ancillary}}{N_{target}}\right),\ \ 1\right) \times \lambda \tag{1}$$

where λ is an overall 'forgetting factor' used to further down-weight the ancillary data, estimated as a hyperparameter using 4-fold cross-validation. Specifically, λ is tuned to minimize the mean squared error (MSE) of model predictions on the held-out folds. Introducing λ is crucial to correct for potential predictor–outcome association shift, as the membership propensity score only adjusts for case-mix shift.

After selecting the optimal λ, we proceed to the next step, fitting a weighted regression model with the K set of predictors. In this model, each individual from the ancillary dataset is weighted in the log-likelihood according to their relatedness to the target, while individuals from the target dataset are assigned a weight of 1. Let individual i have a corresponding weight w_i from weights set that was calculated based on the importance of each individual. These weights are then included in the log-likelihood (LL) of the model as follows:

$$\text{LL} = \sum_{i=1}^{N} \big(Y_i \times \log(P_i) + (1 - Y_i) \times \log(1 - P_i)\big) \times w_i \tag{2}$$

where P is the predicted probability for a binary outcome. We implement this method in two variations as follows:

1. **Recalibration:** We estimate PS in the weighting scheme given the observed values from a set of predictor variables $\boldsymbol{X}_i = (X_{i1}, ..., X_{iK})$ or $\boldsymbol{X}_i^+ = (X_{i1}, ..., X_{iK}, U_i)$ only. Then after developing the weighted model, we adjust the calibration in the large (CITL) of the weighted model through adding a dummy variable for the target membership R. The dummy variable R indicates the absence or presence of the effect of target membership that causes a shift for the outcome, where it takes a value of 0 if the samples belong to target, and a value of 1 if the samples belong to ancillary. We add the dummy variable as an additional predictor in the developed model.

2. **Including the outcome Y in the weighting scheme:** As an alternative to Recalibration, we estimate PS in the weighting scheme given the observed values from a set of predictor variables $\boldsymbol{X}_i$ including the outcome Y as predictor, where $\boldsymbol{X}_i^+ = (X_{i1}, ..., X_{iK}, Y_i)$ or $\boldsymbol{X}_i^+ = (X_{i1}, ..., X_{iK}, U_i, Y_i)$, without adding a dummy variable in the developed weighted model afterwards.

S3 Results

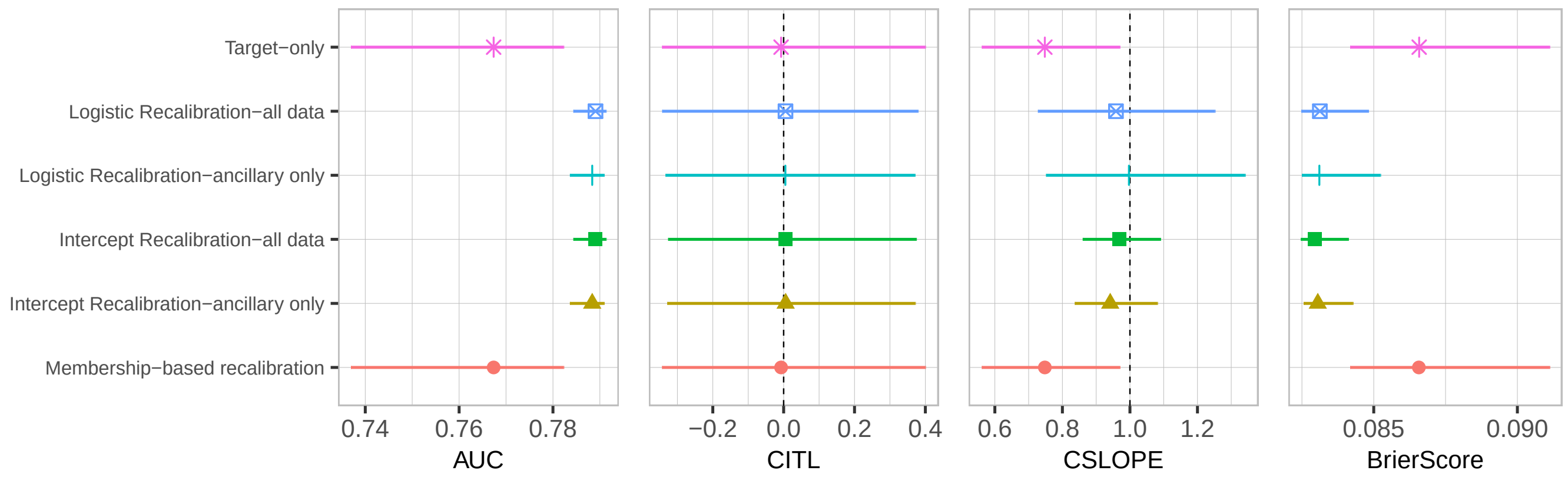

Figure S1: Performance metrics for high degree of single Case-mix shift
Symbols and error bars correspond to the median and 95% confidence interval for each model on the y-axis across 200 iterations.

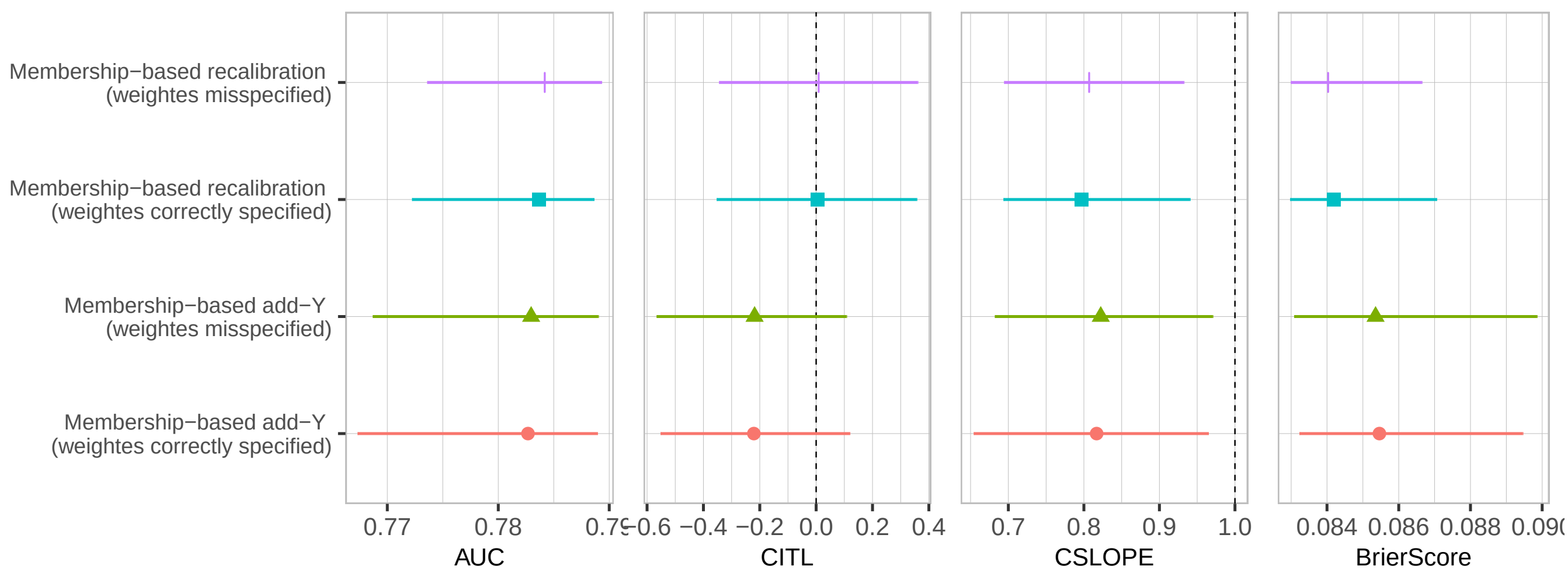

Figure S2: Correctly Specified vs. Misspecified Weighting Schemes results for Combination of single Case-mix, Event rate and Predictor-outcome association shifts
Symbols and error bars correspond to the median and 95% confidence interval for each model on the y-axis across 200 iterations.

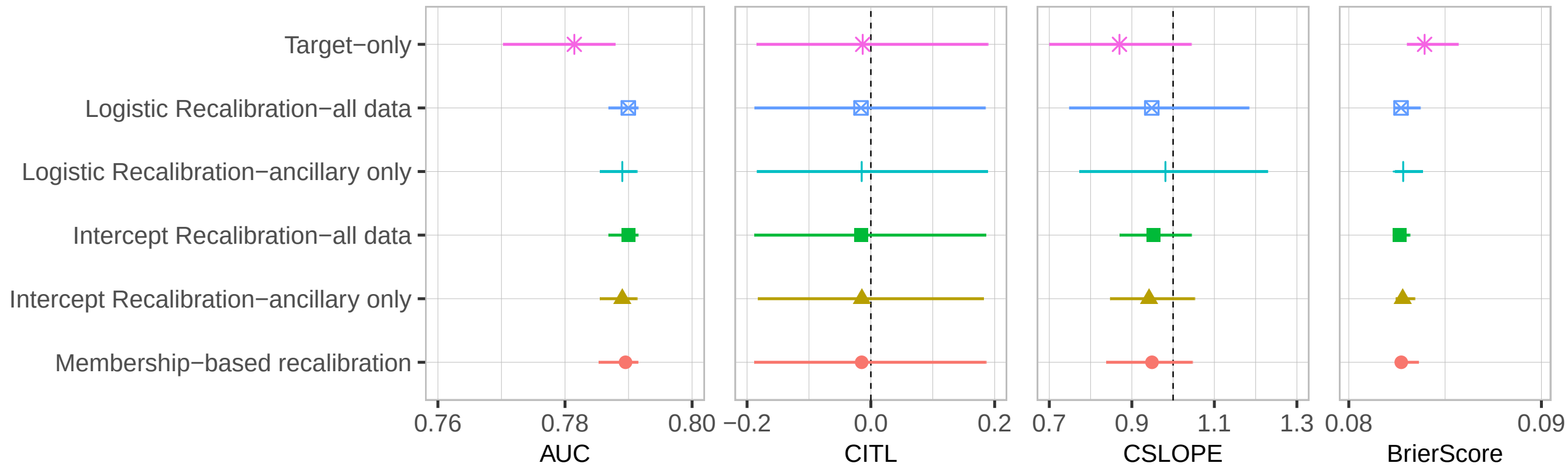

(a) 75% ***source*** : 25% ***target***

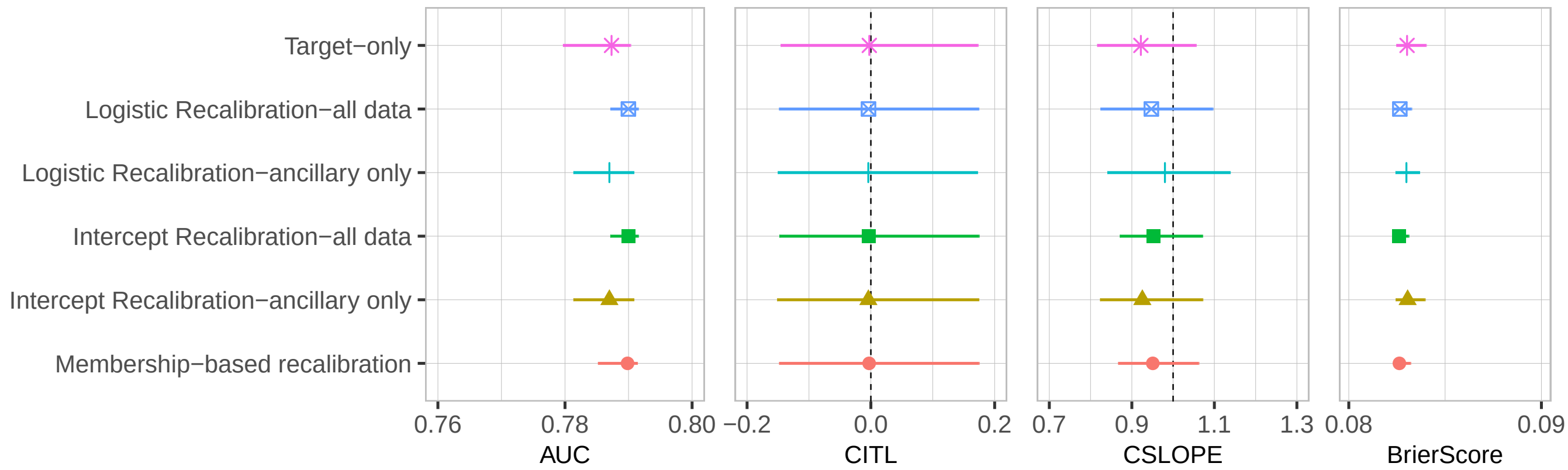

(b) 50% ***source*** : 50% ***target***

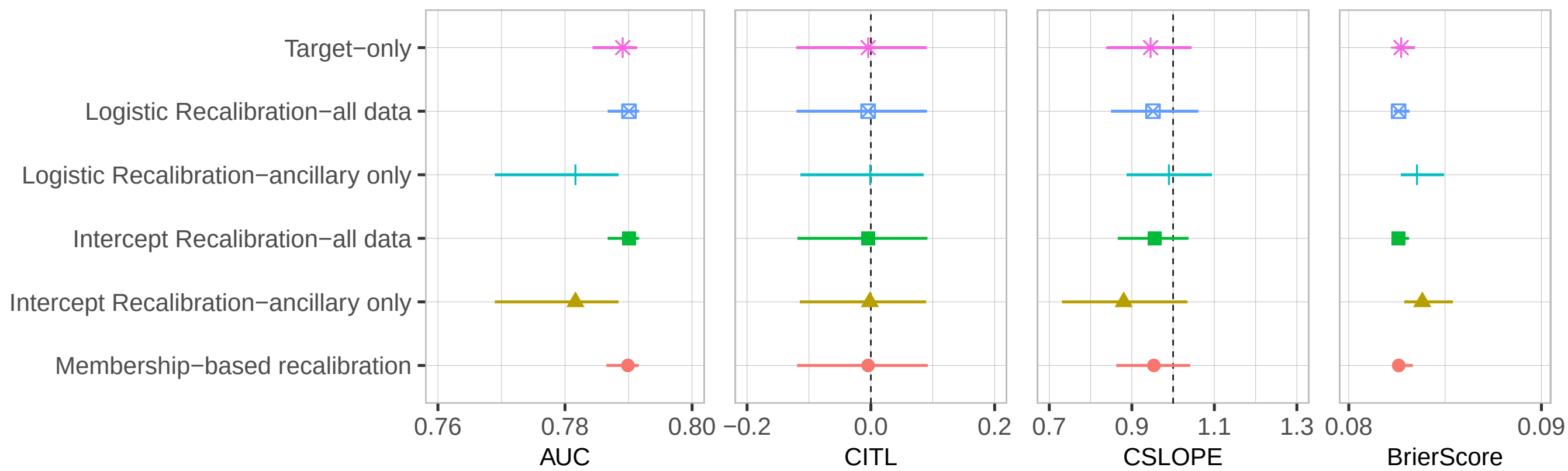

(c) 25% ***source*** : 75% ***target***

Figure S3: Proportion split of ***source*** and ***target*** for No Shift
Symbols and error bars correspond to the median and 95% confidence interval for each model on the y-axis across 200 iterations. Outcome-generation model with 15 predictors and total sample size is $4 \times N_{min}$

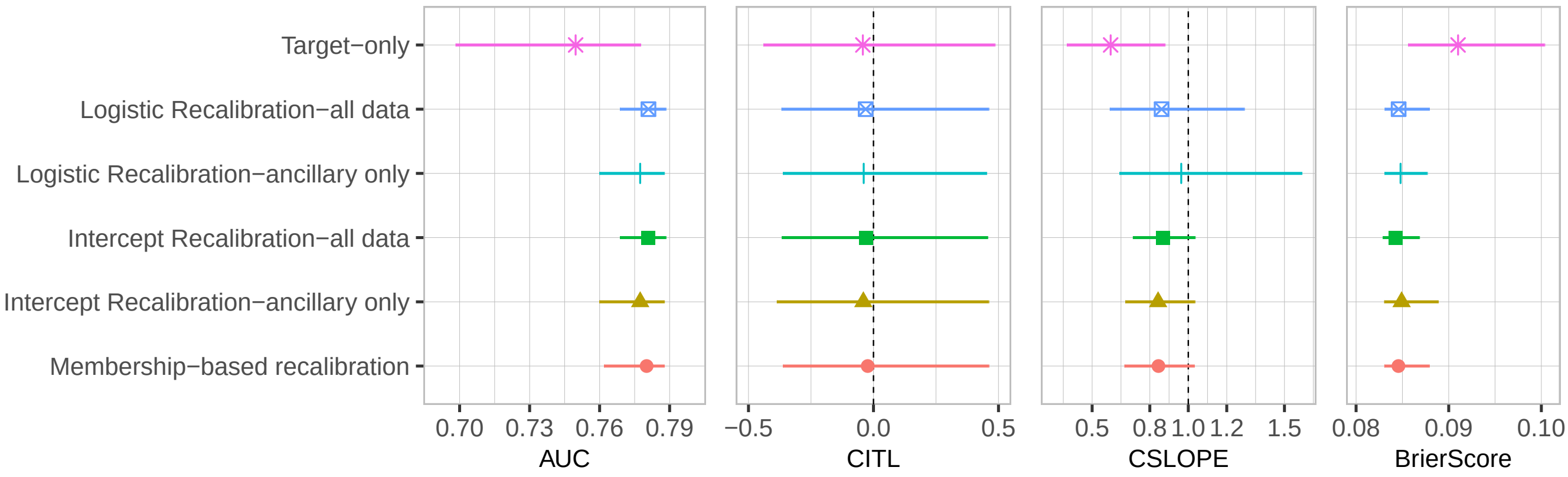

(a) (N_{min})
source and ***target*** insufficient

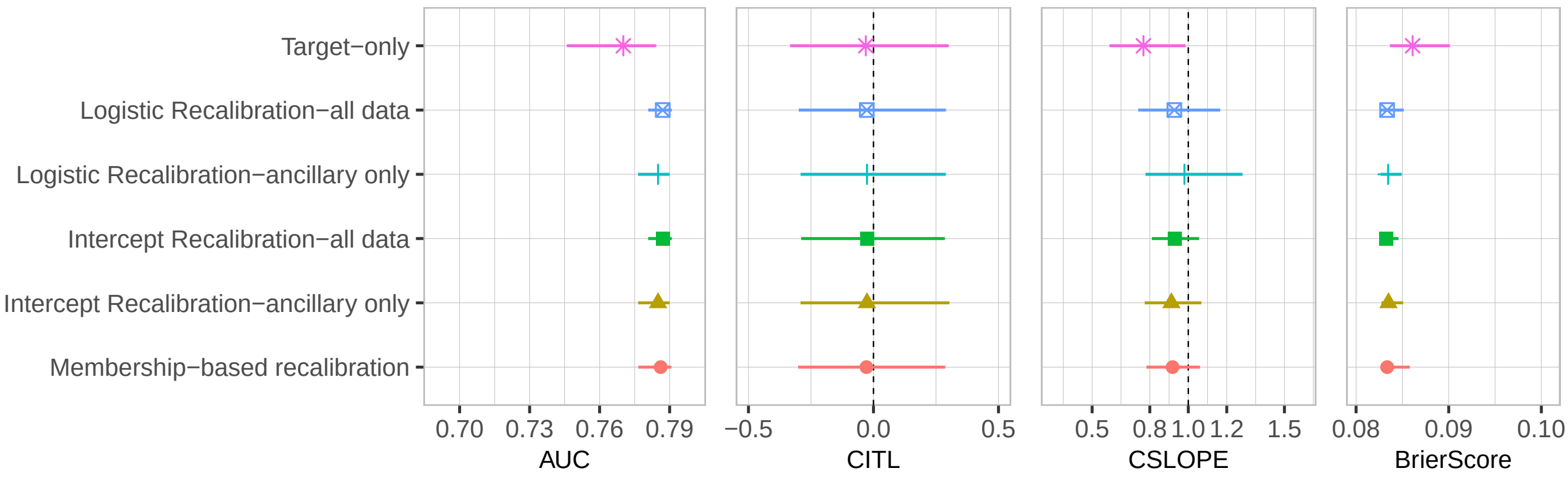

(b) ($2 \times N_{min}$)
source sufficient/***target*** insufficient

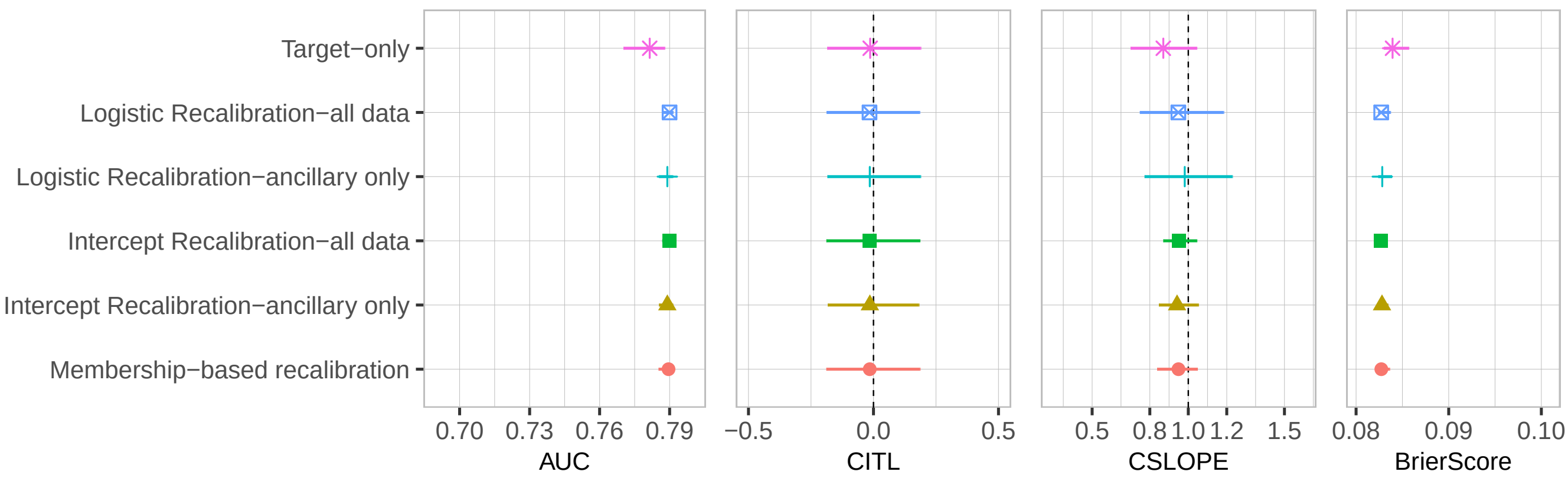

(c) ($4 \times N_{min}$)
source and ***target*** sufficient

Figure S4: Performance metrics with different sample size for No Shift
Symbols and error bars correspond to the median and 95% confidence interval for each model on the y-axis across 200 iterations. Outcome-generation model with 15 predictors and proportion split is "**75% ancillary : 25% target**"

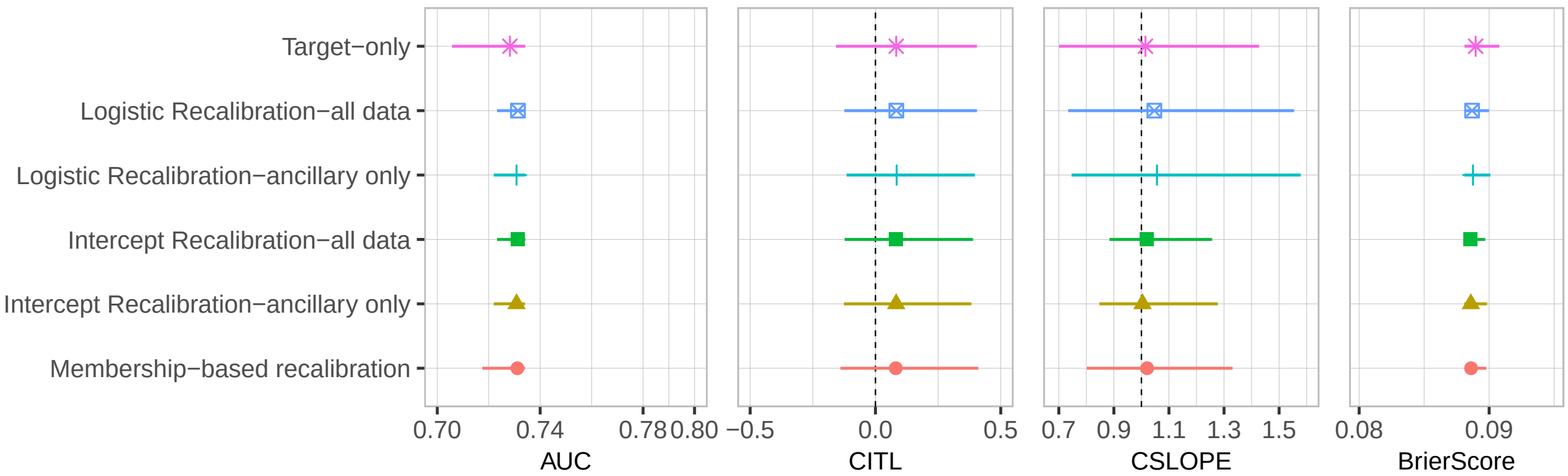

(a) Model with 4 predictor variables

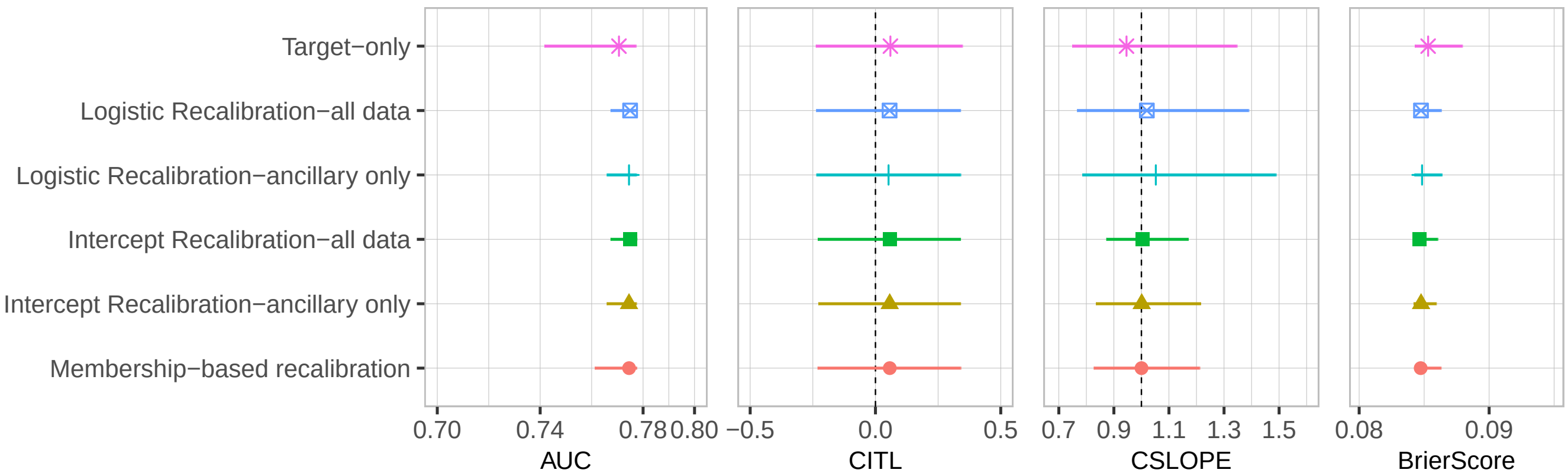

(b) Model with 5 predictor variables

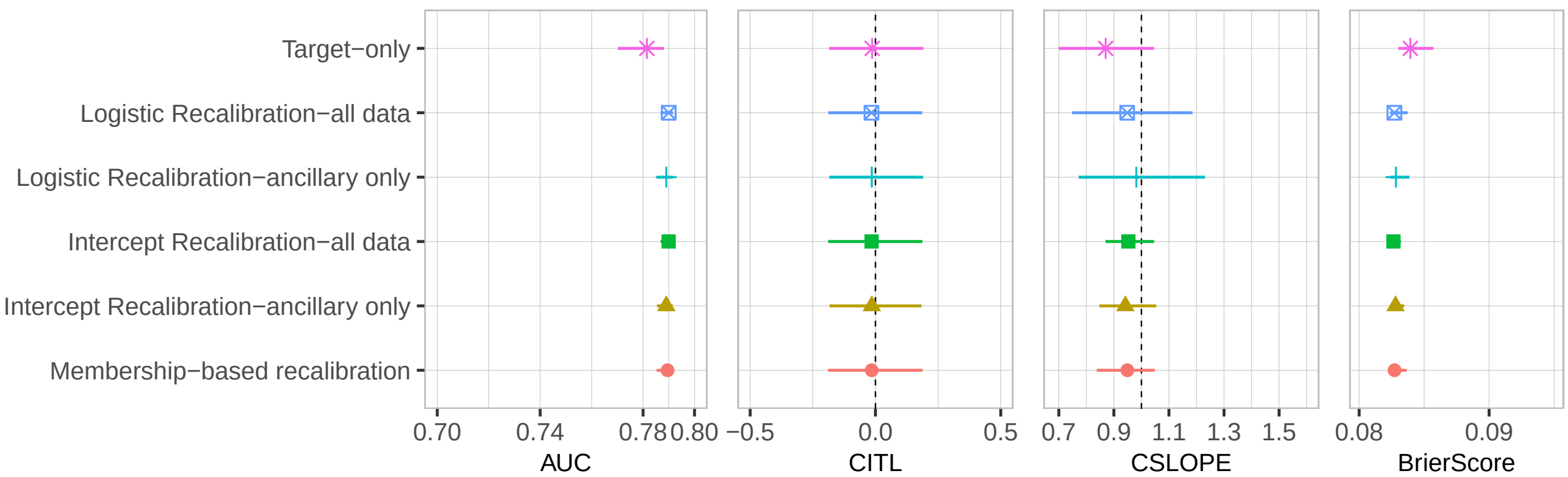

(c) Model with 15 predictor variables

Figure S5: Performance metrics with different number of predictor variables for No Shift
Symbols and error bars correspond to the median and 95% confidence interval for each model on the y-axis across 200 iterations.

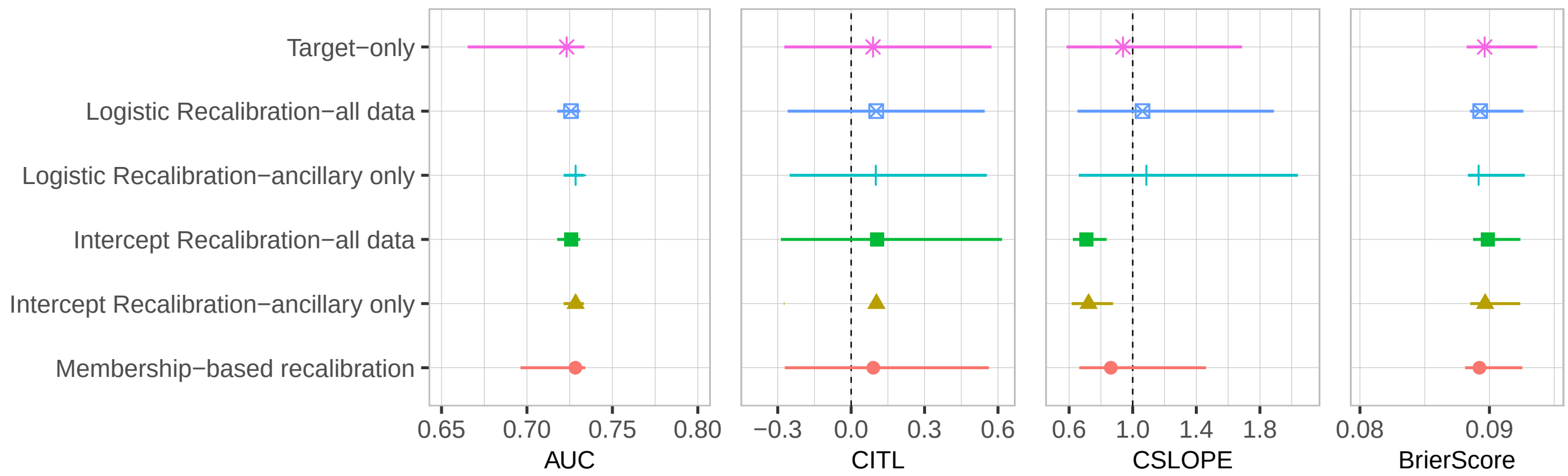

(a) Model with 4 predictor variables

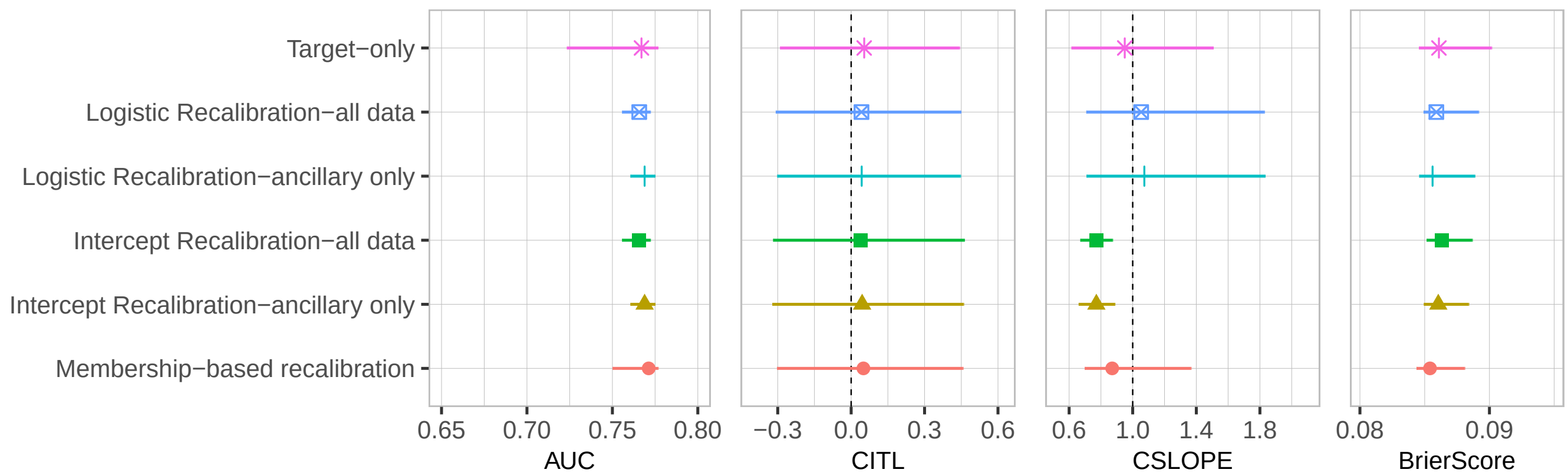

(b) Model with 5 predictor variables

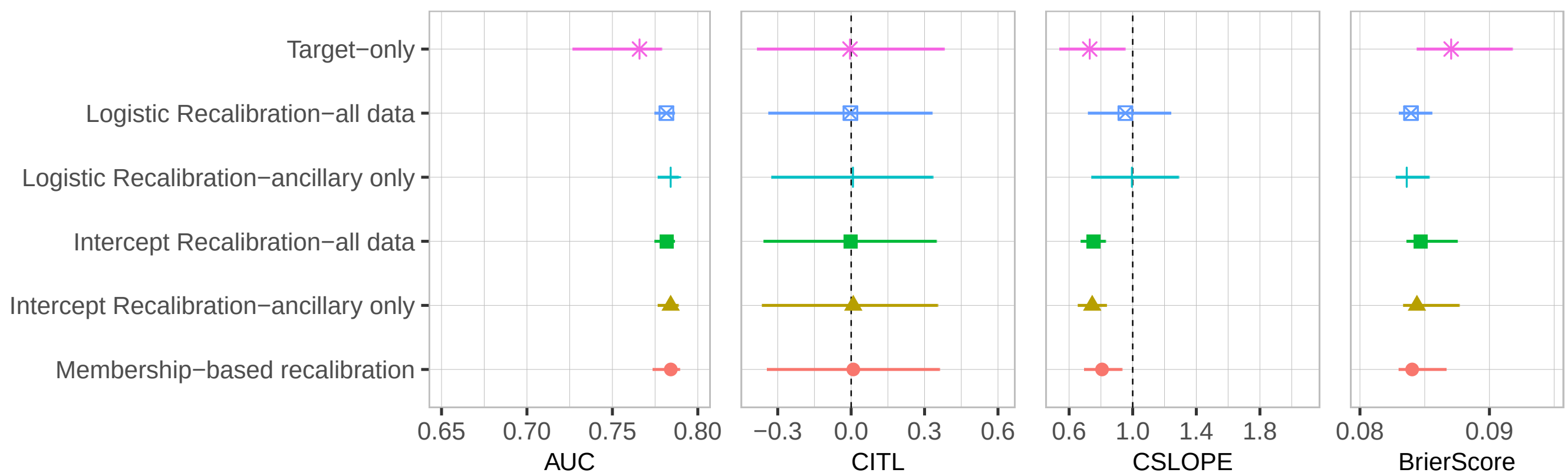

(c) Model with 15 predictor variables

Figure S6: Performance metrics with different number of predictor variables for Combination of single Case-mix, Event rate and Predictor-outcome association shifts

Symbols and error bars correspond to the median and 95% confidence interval for each model on the y-axis across 200 iterations.
Total sample size is $4 \times N_{min}$ and proportion split is "**75% ancillary : 25% target**"

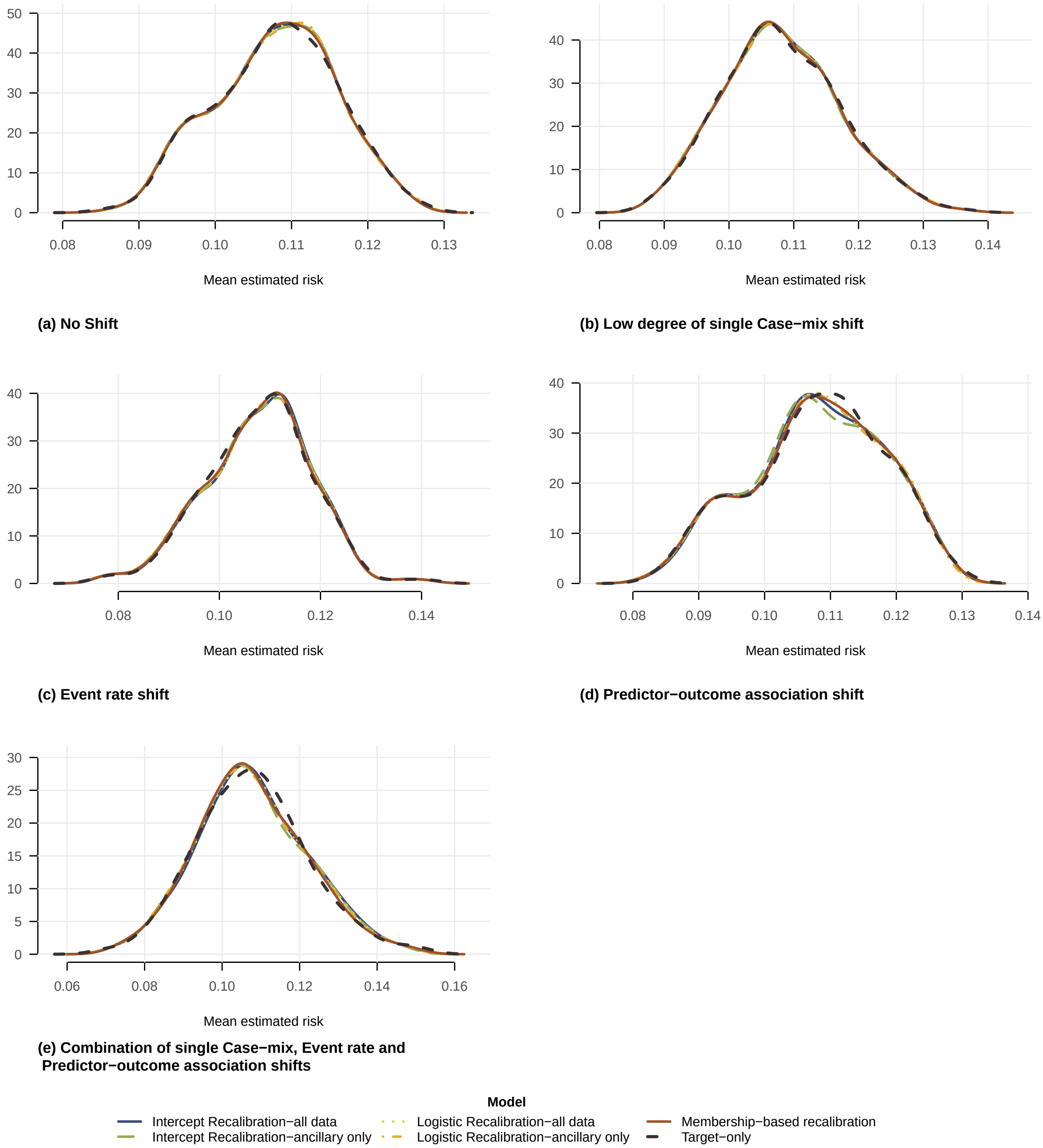

Figure S7: Instability in Mean Estimated Risk

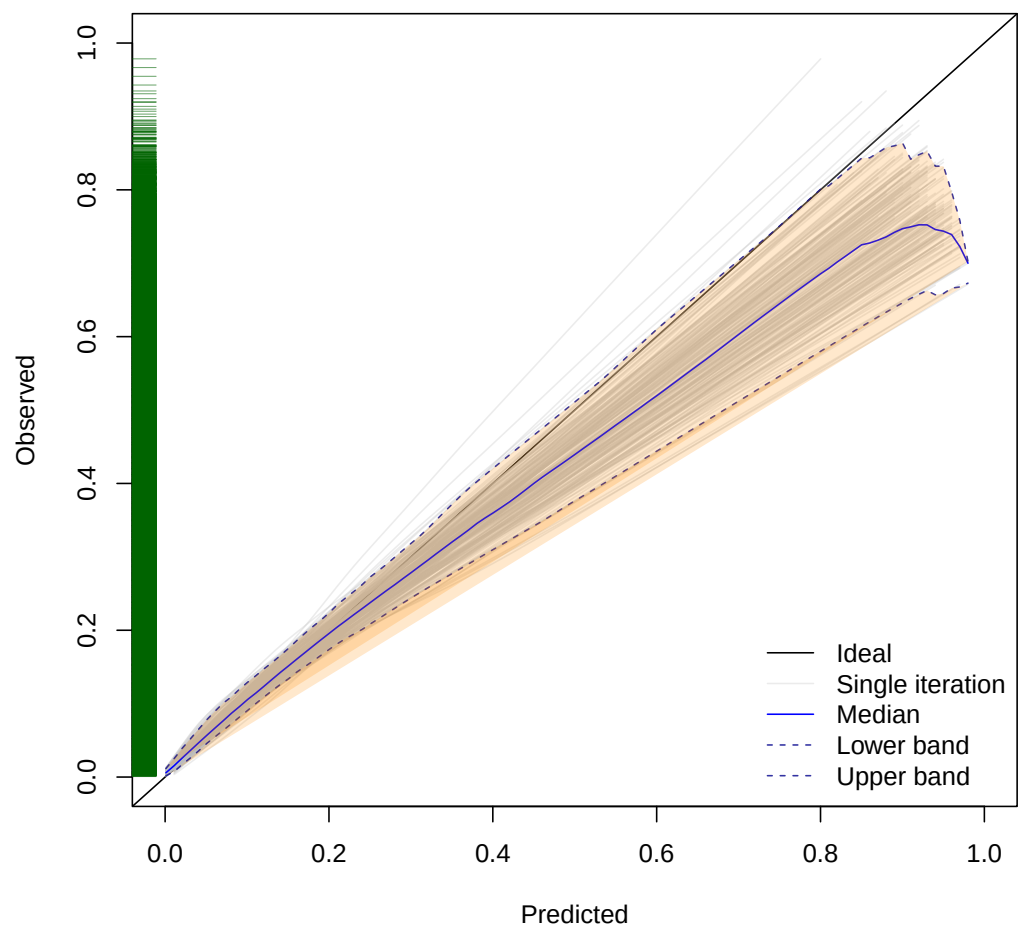

(a) Target-only

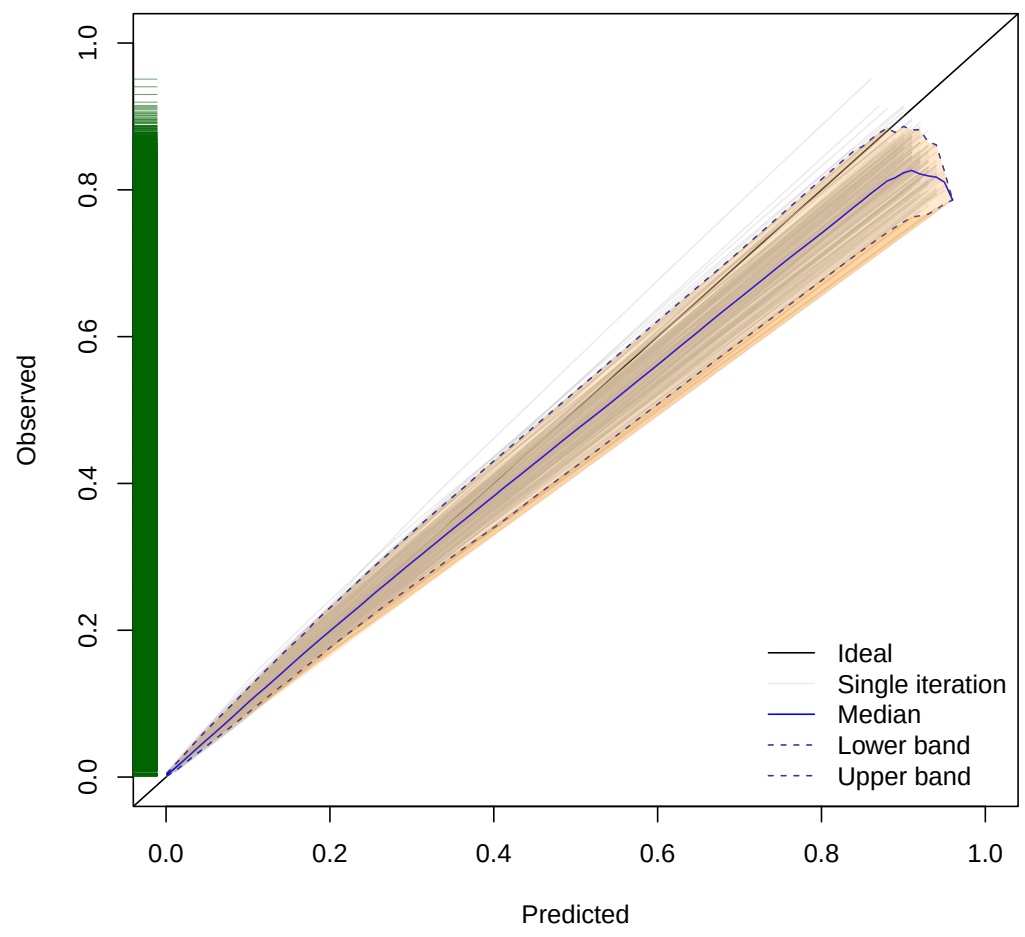

(b) Intercept Recalibration-all data

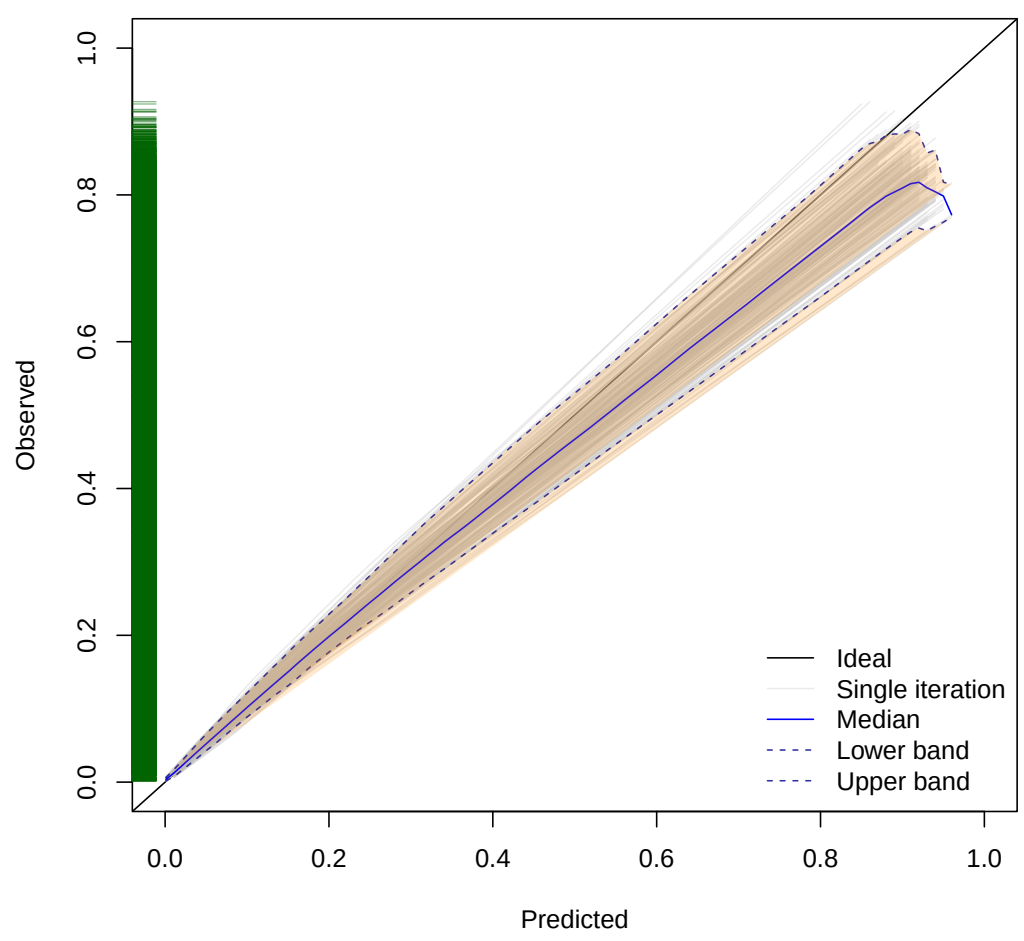

(c) Intercept Recalibration-ancillary only

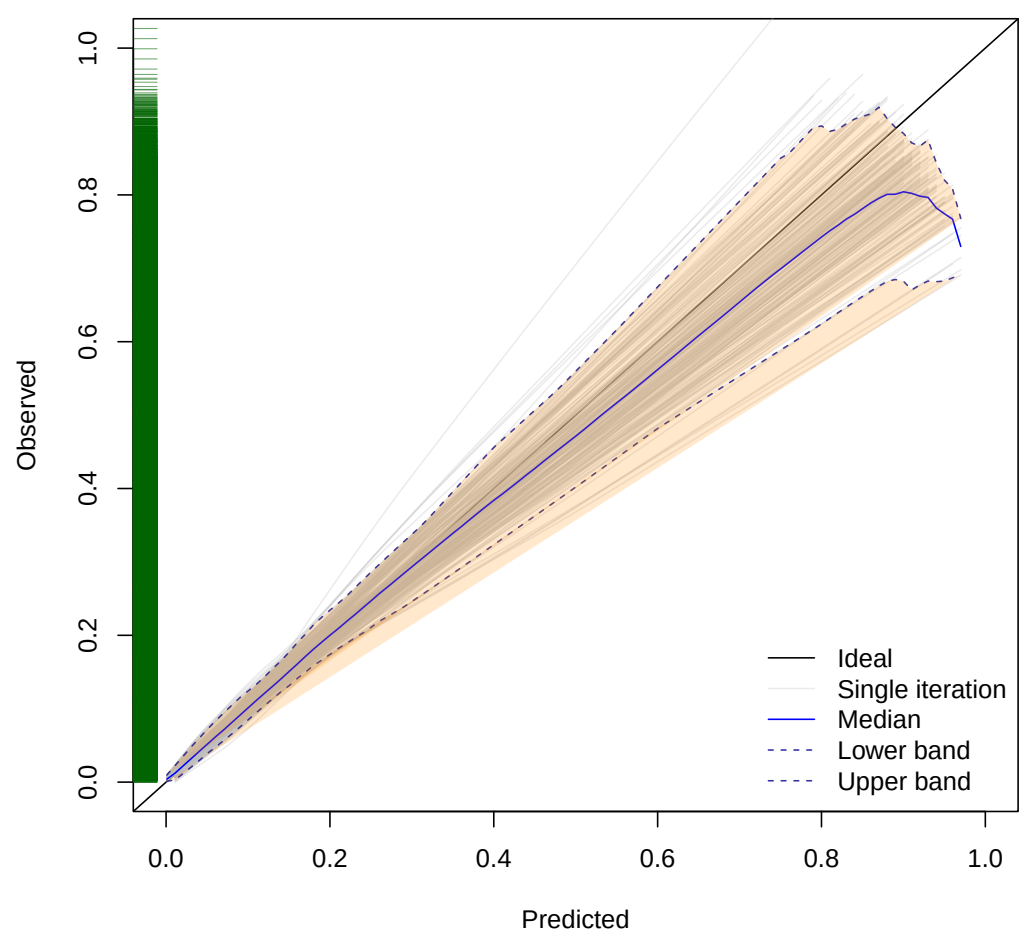

(d) Logistic Recalibration-all data

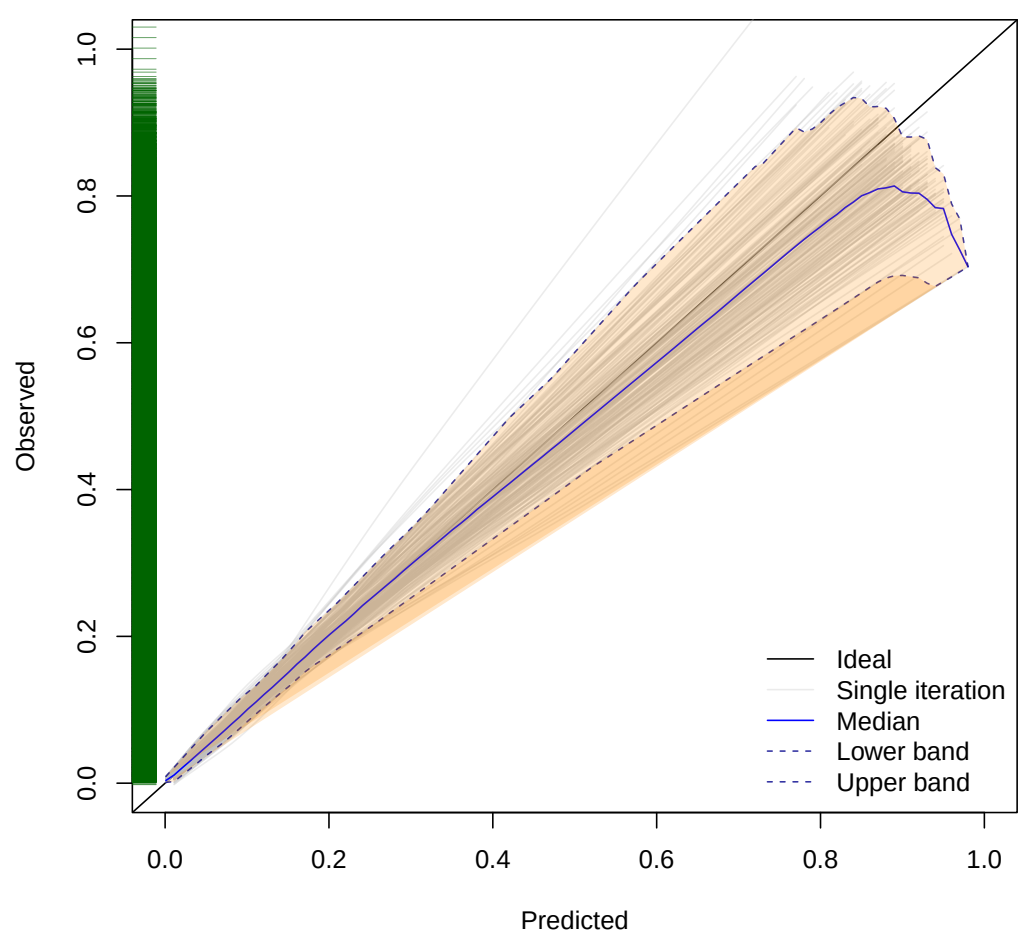

(e) Logistic Recalibration-ancillary only

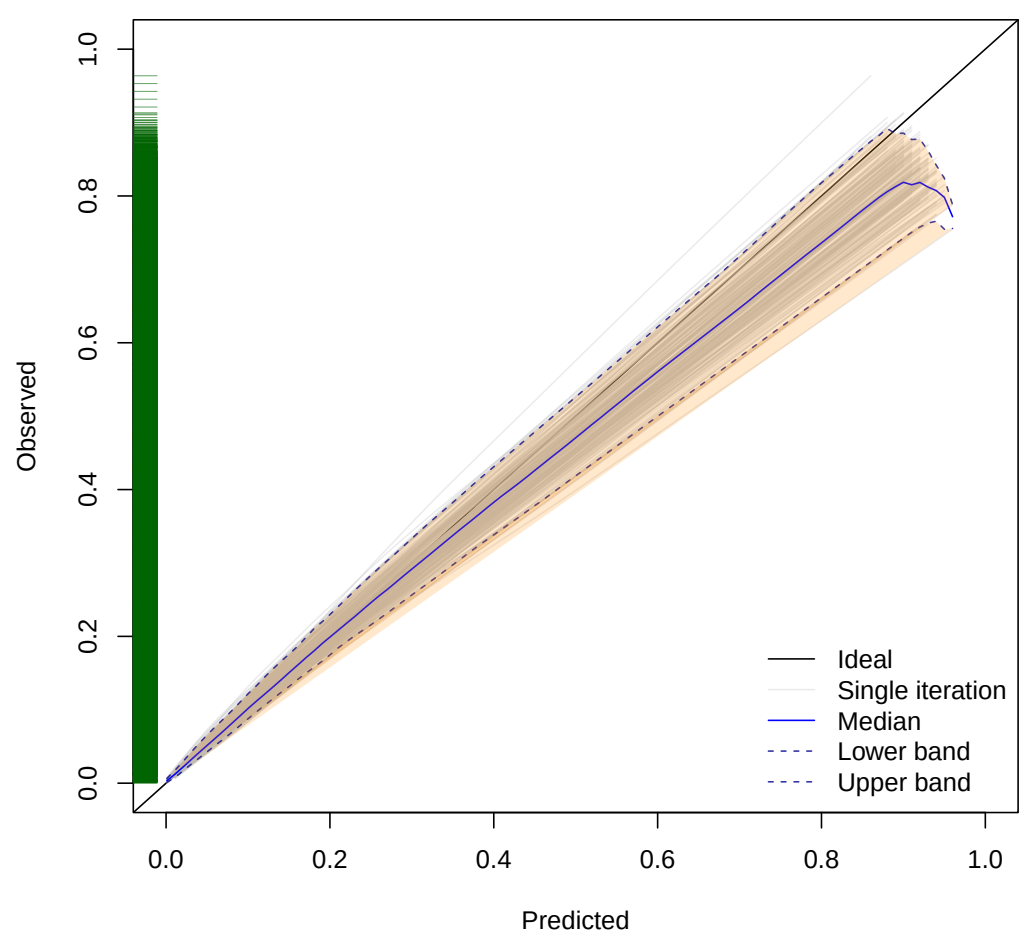

(f) Membership-based recalibration

Figure S8: Individual Calibration Plots for No Shift

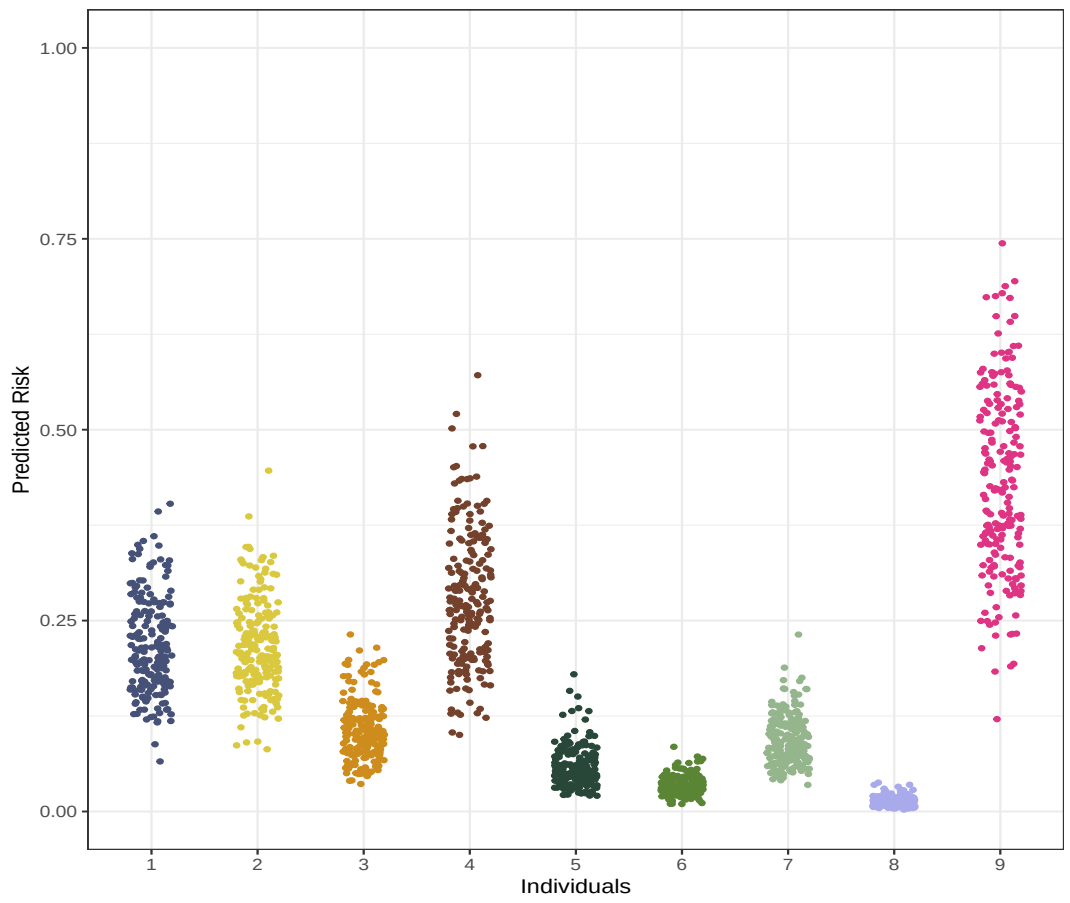

(a) Target-only

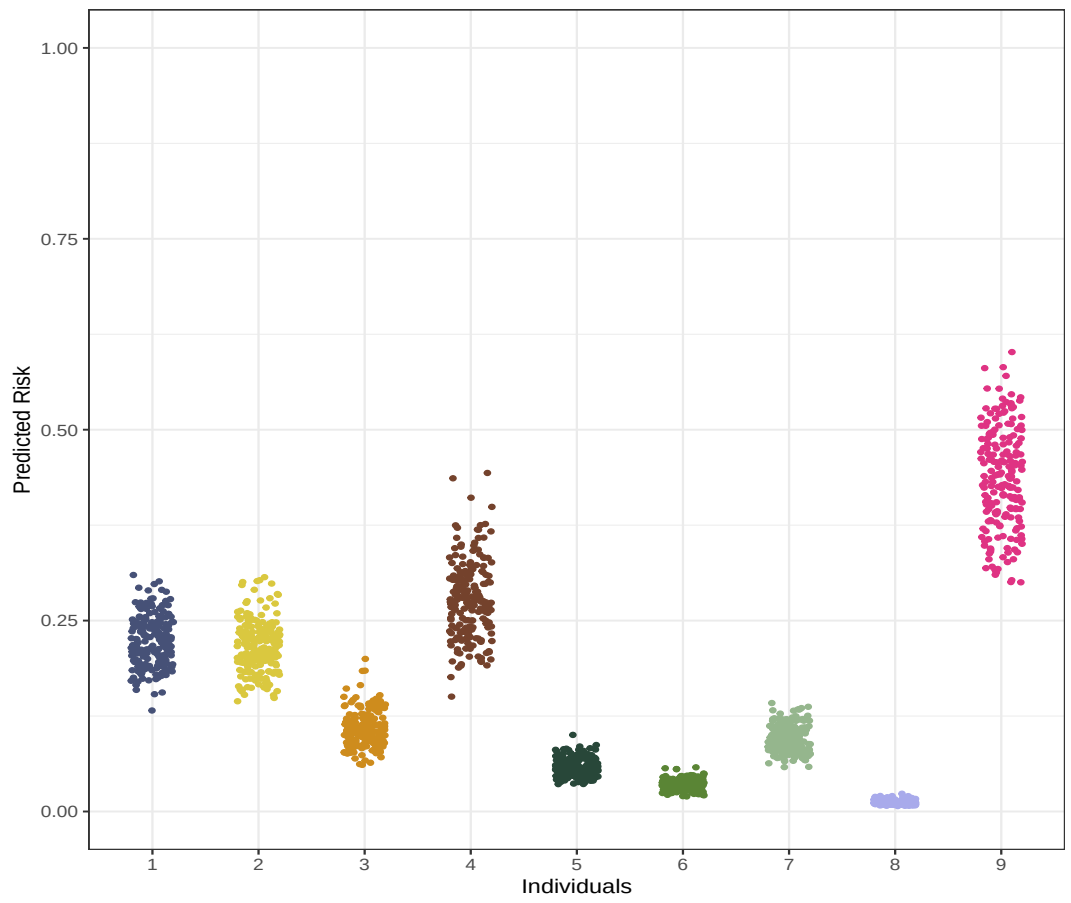

(b) Intercept Recalibration-all data

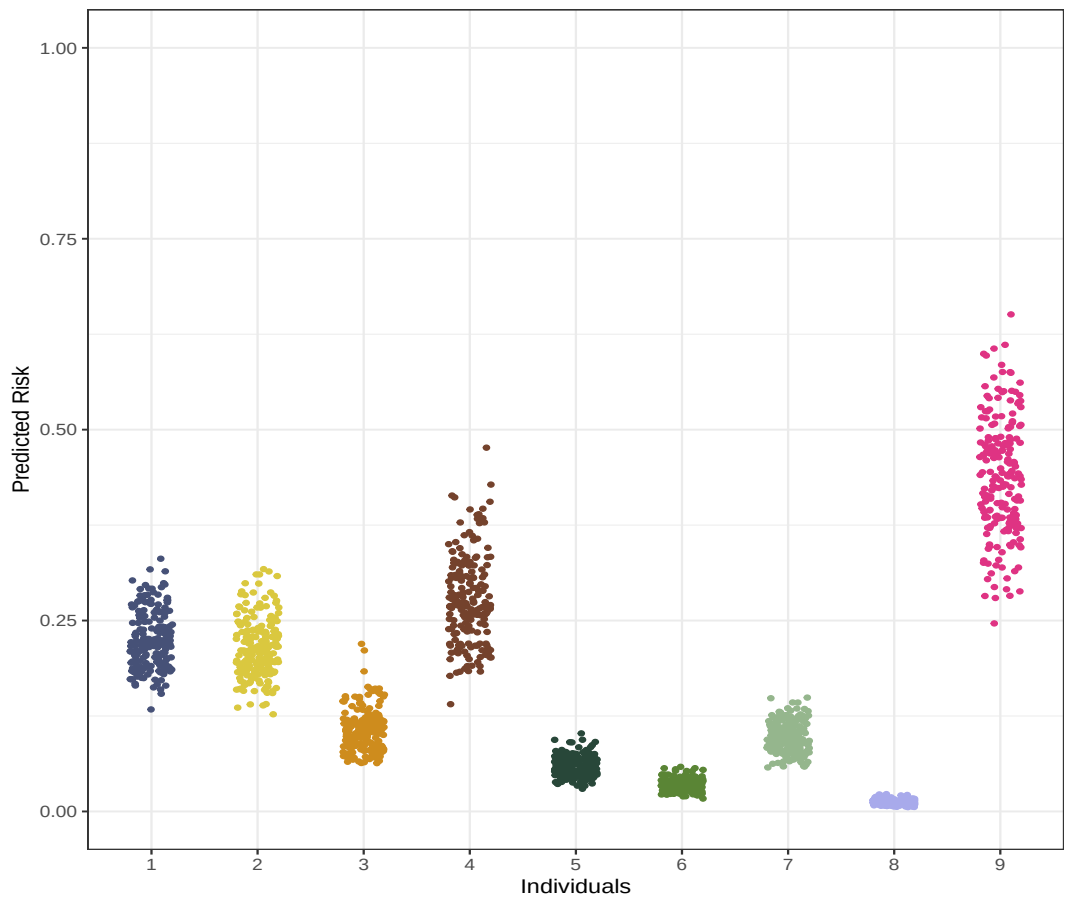

(c) Intercept Recalibration-ancillary only

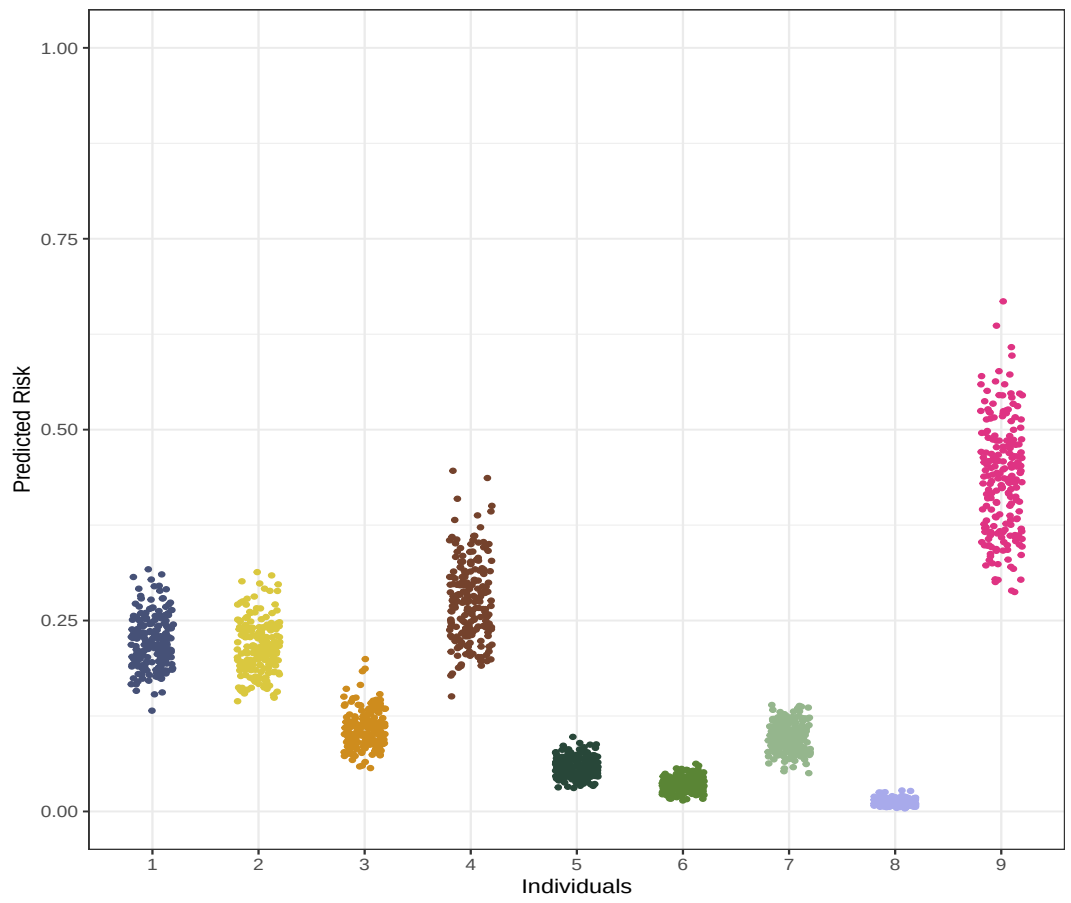

(d) Logistic Recalibration-all data

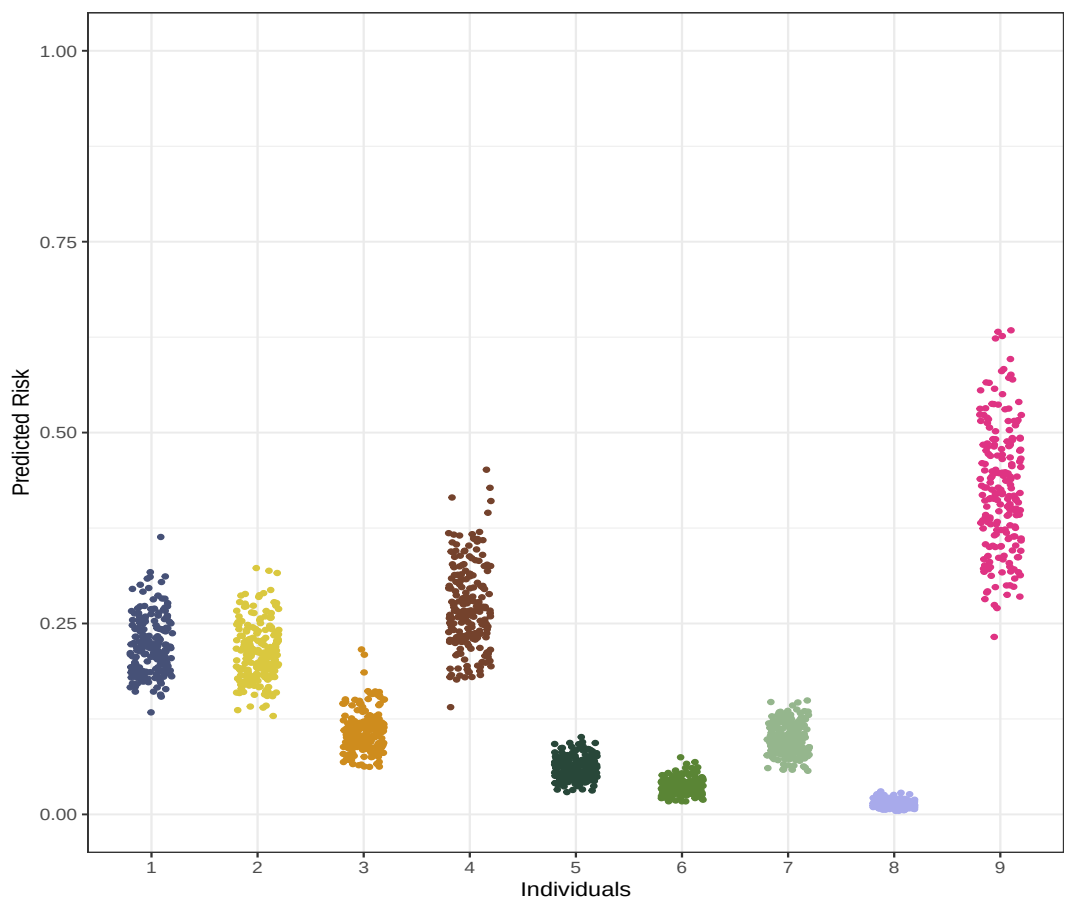

(e) Logistic Recalibration-ancillary only

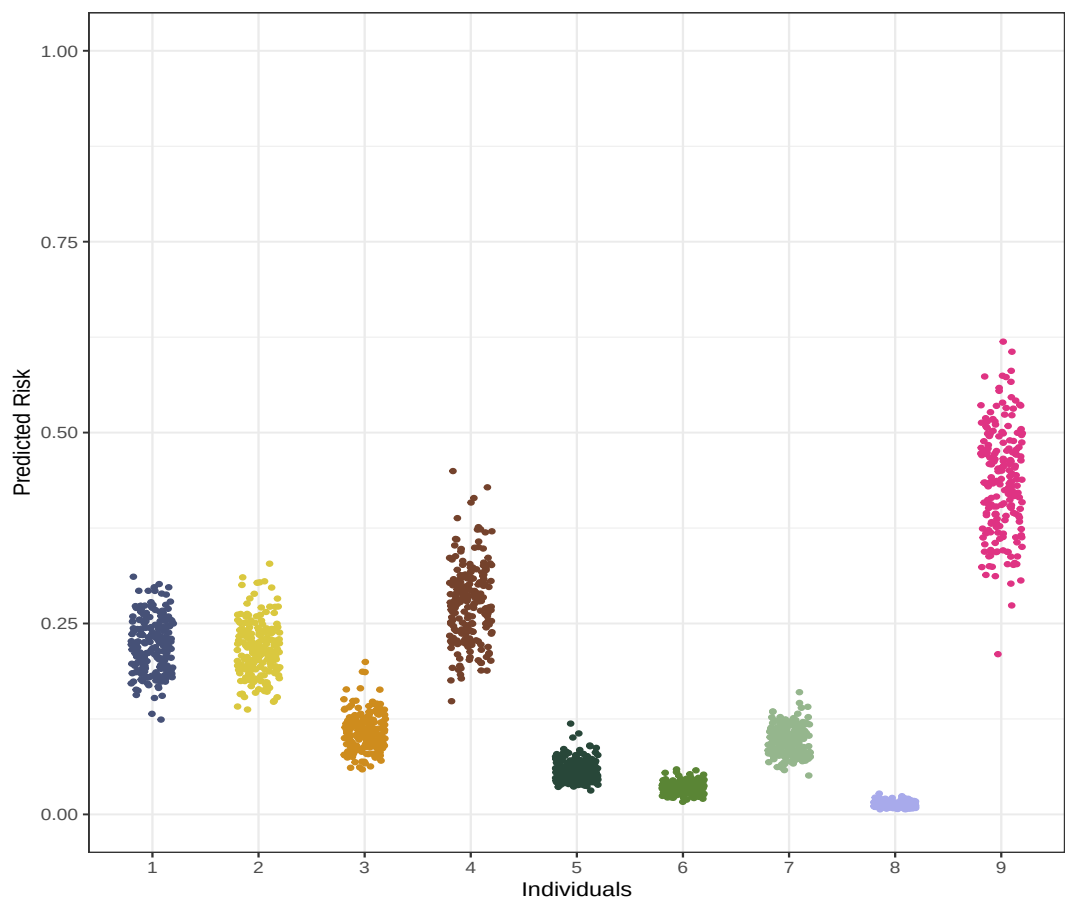

(f) Membership-based recalibration

Figure S9: Instability of estimated risks for nine individuals for No Shift

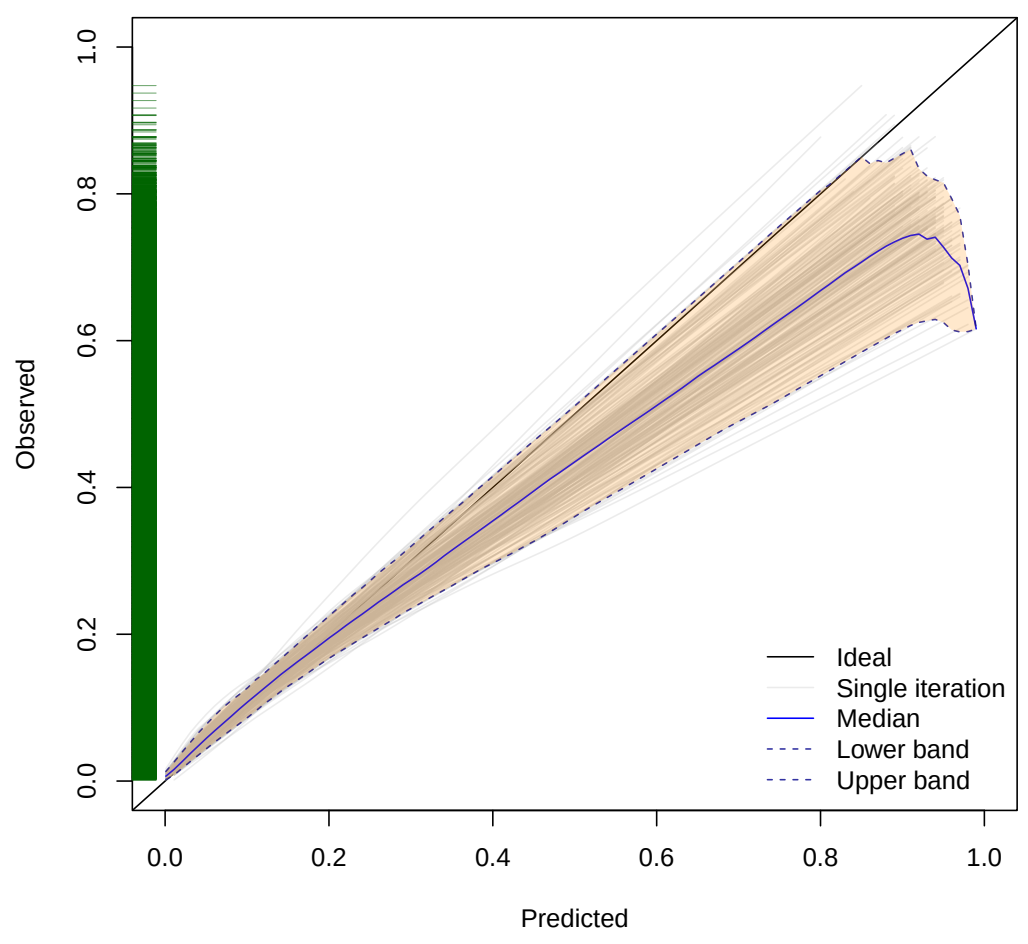

(a) Target-only

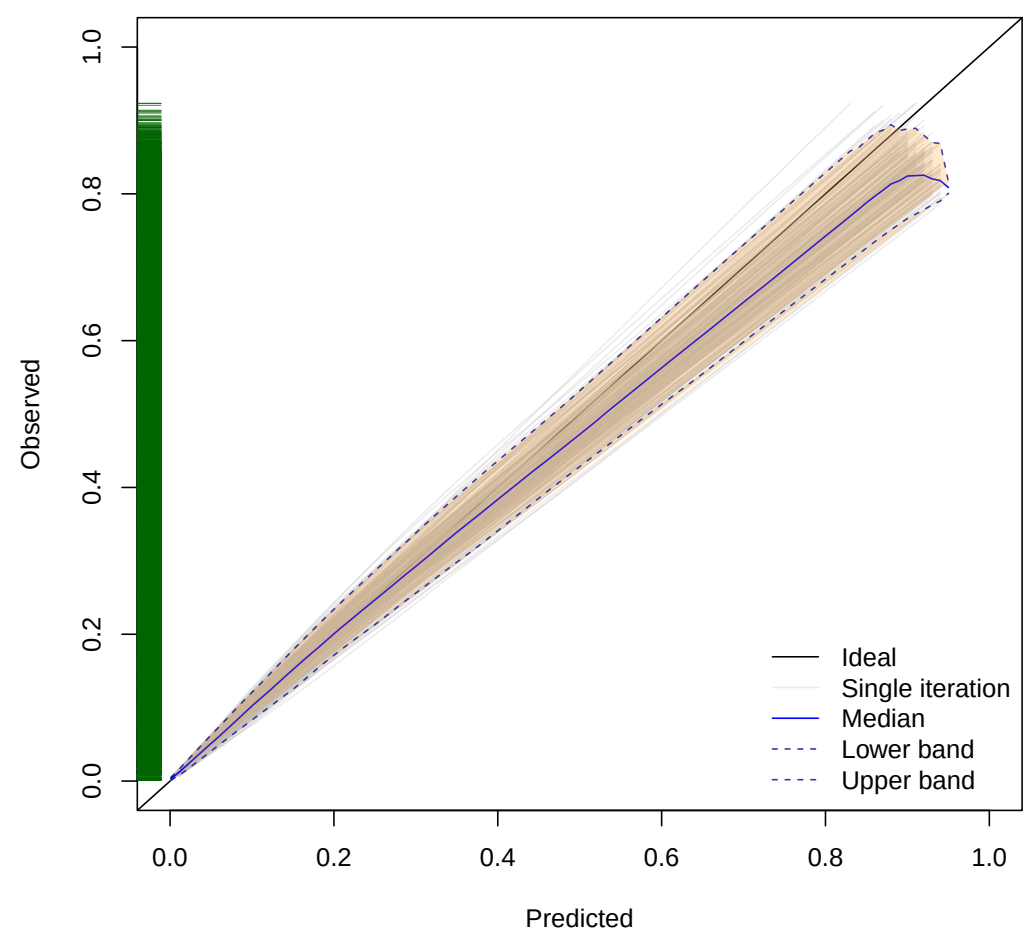

(b) Intercept Recalibration-all data

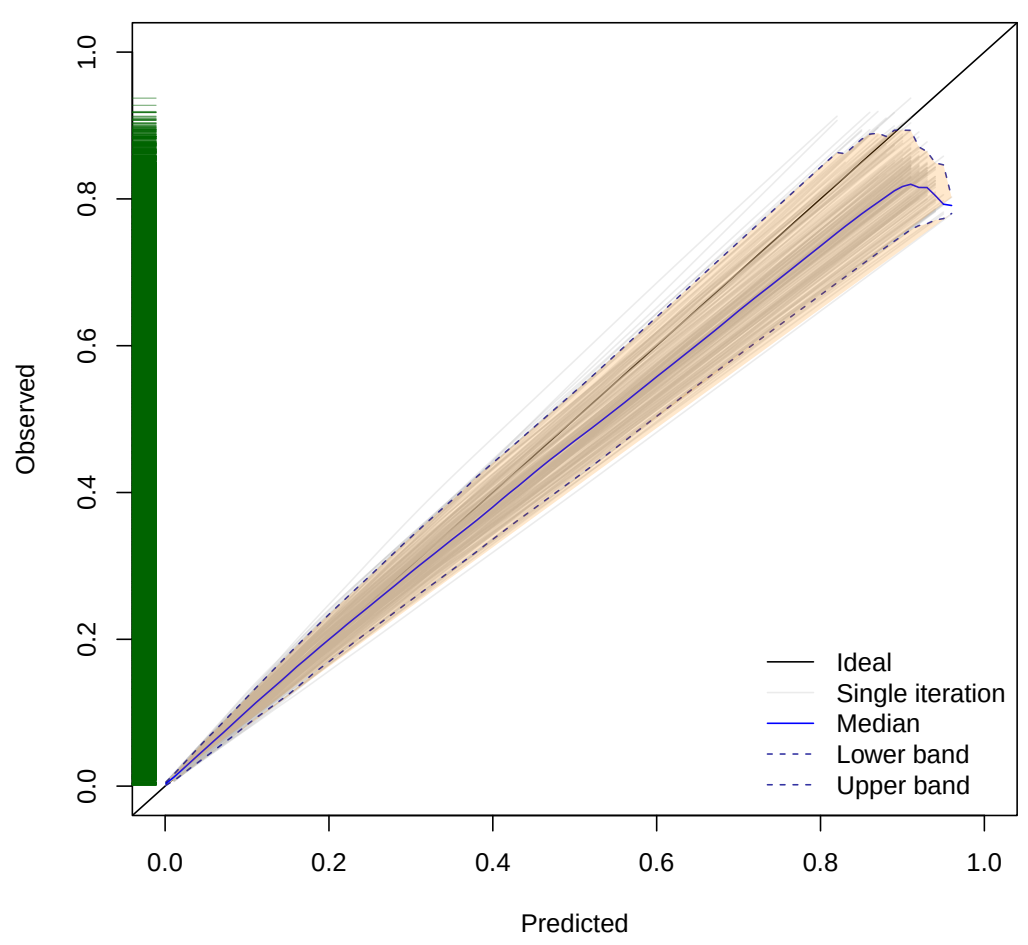

(c) Intercept Recalibration-ancillary only

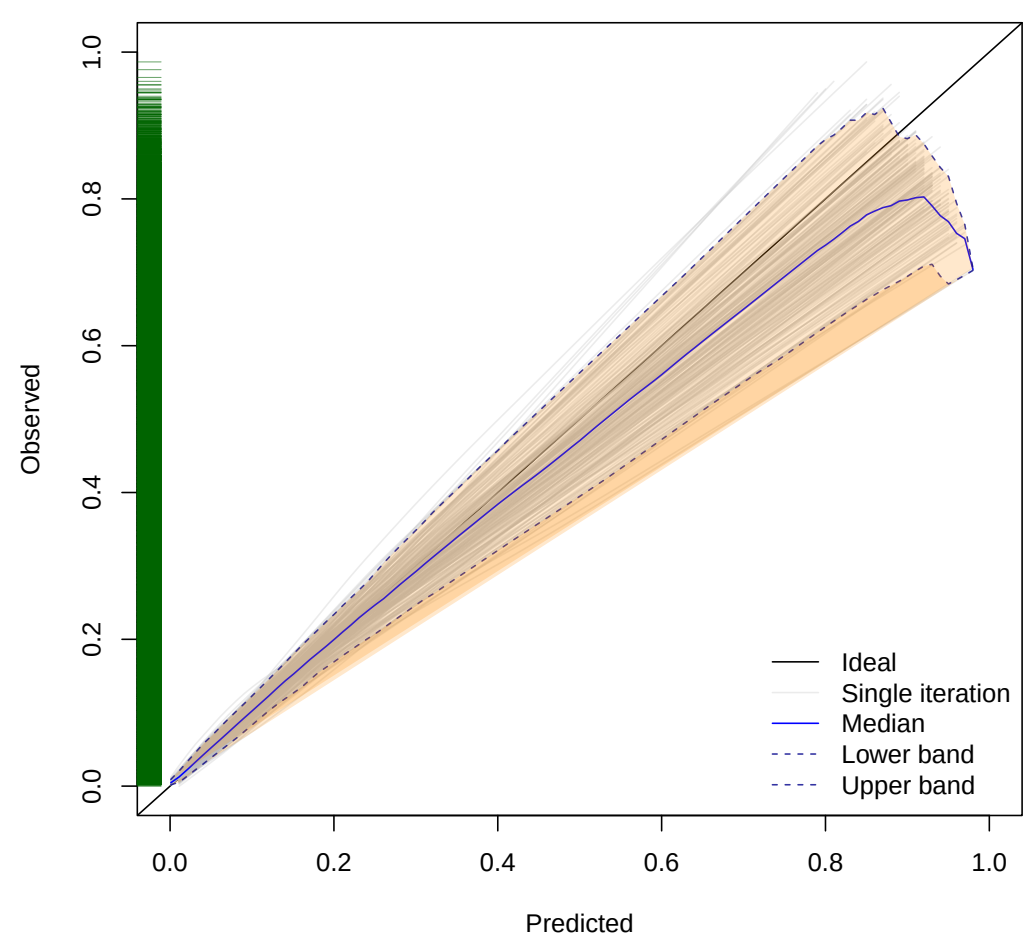

(d) Logistic Recalibration-all data

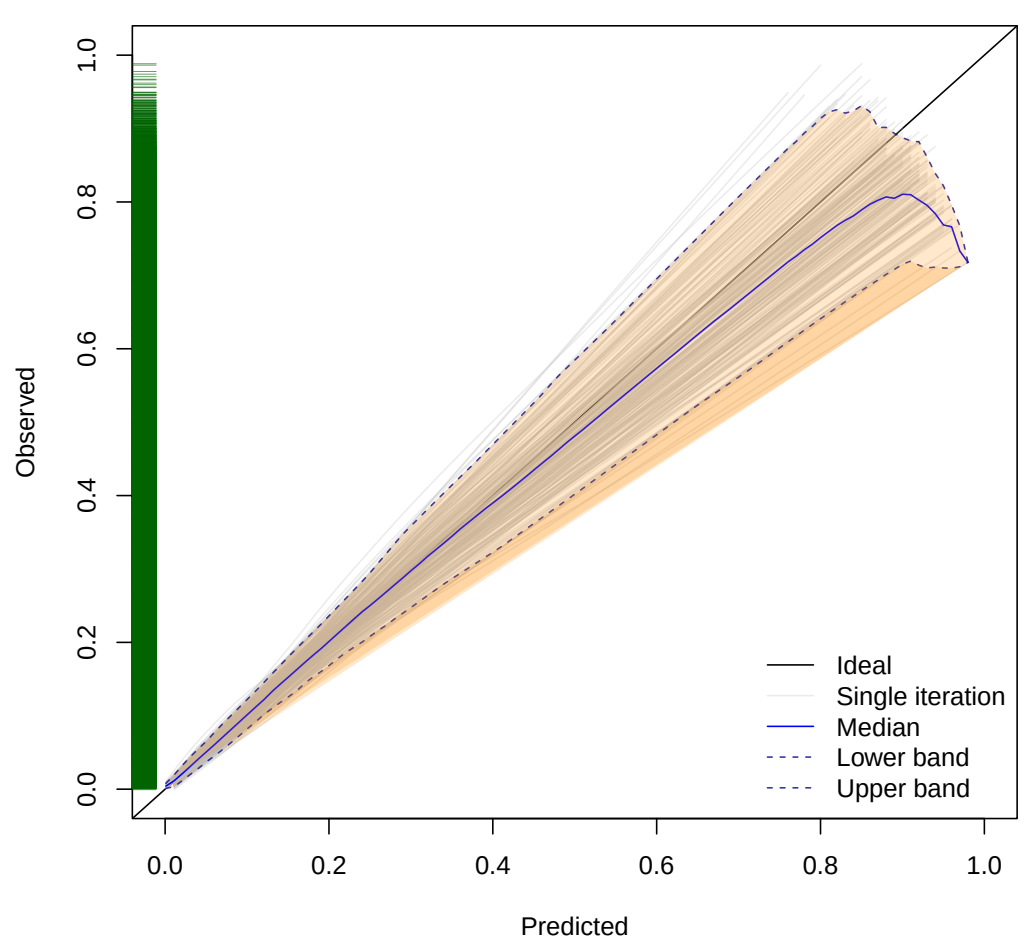

(e) Logistic Recalibration-ancillary only

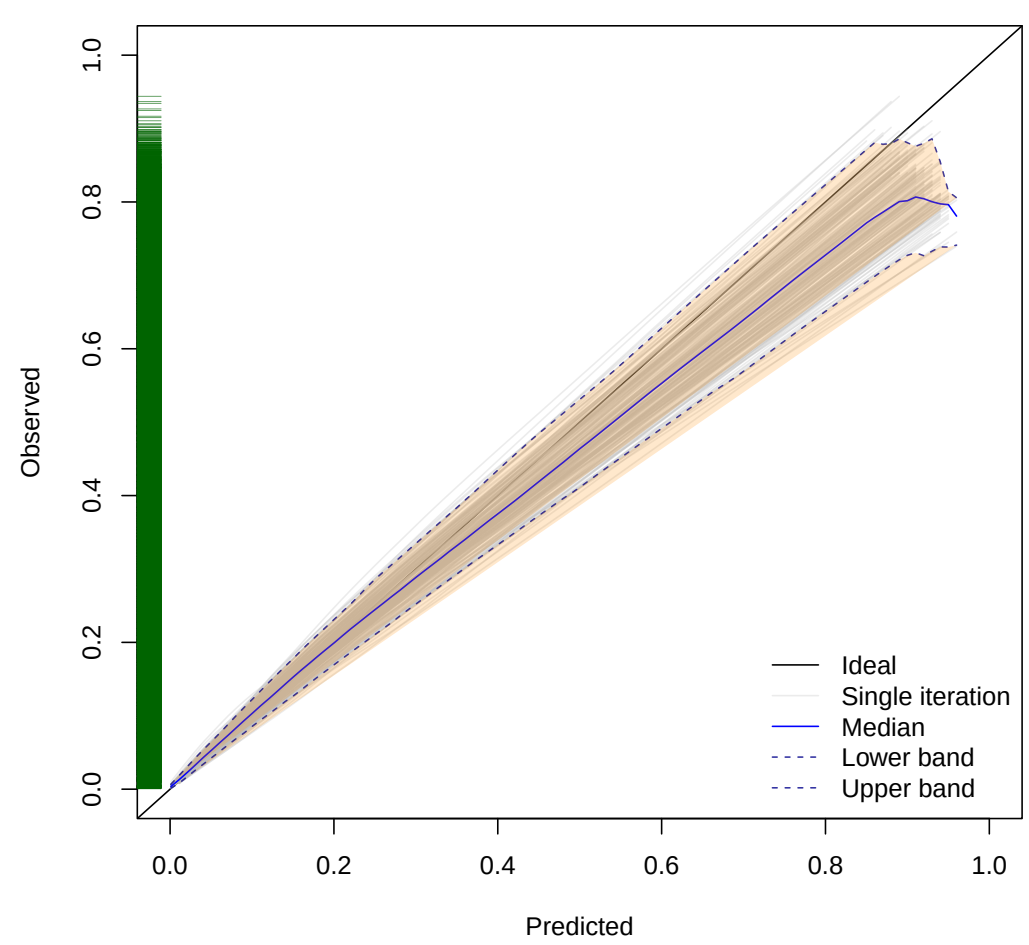

(f) Membership-based recalibration

Figure S10: Individual Calibration Plots for low degree of single Case-mix Shift

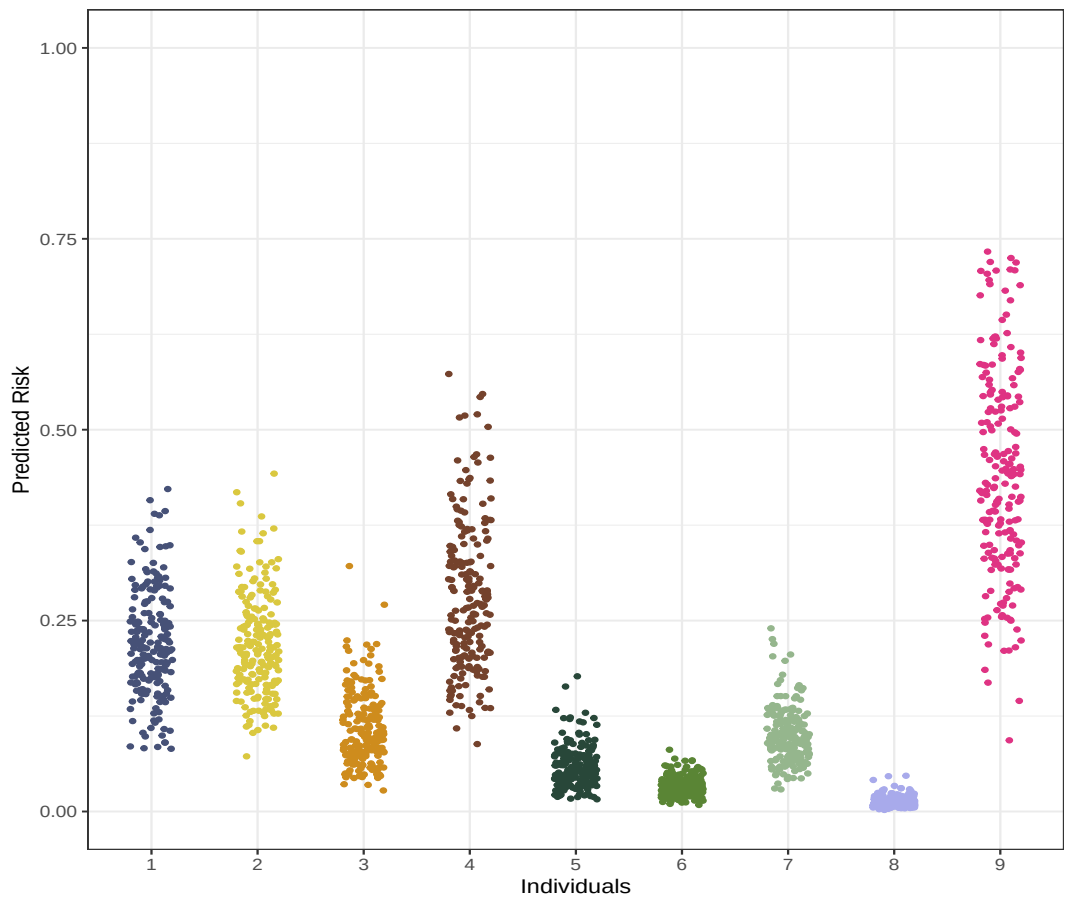

(a) Target-only

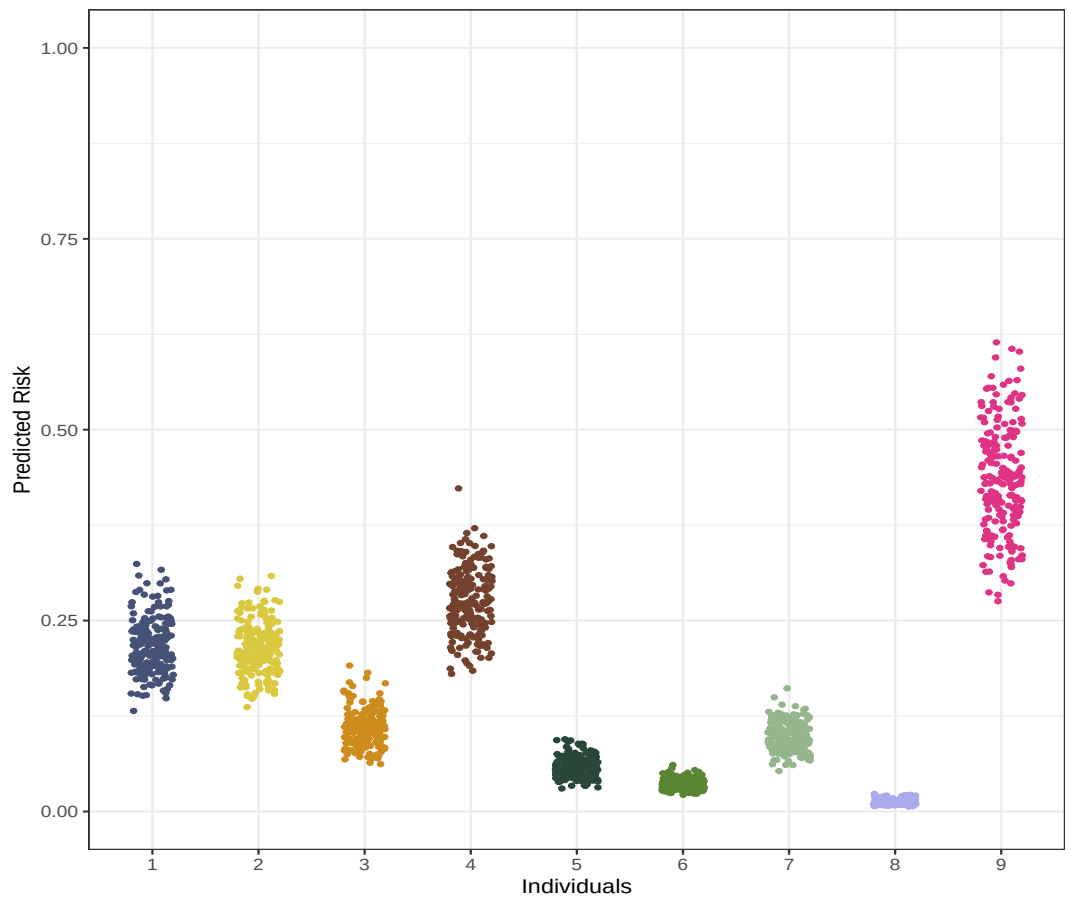

(b) Intercept Recalibration-all data

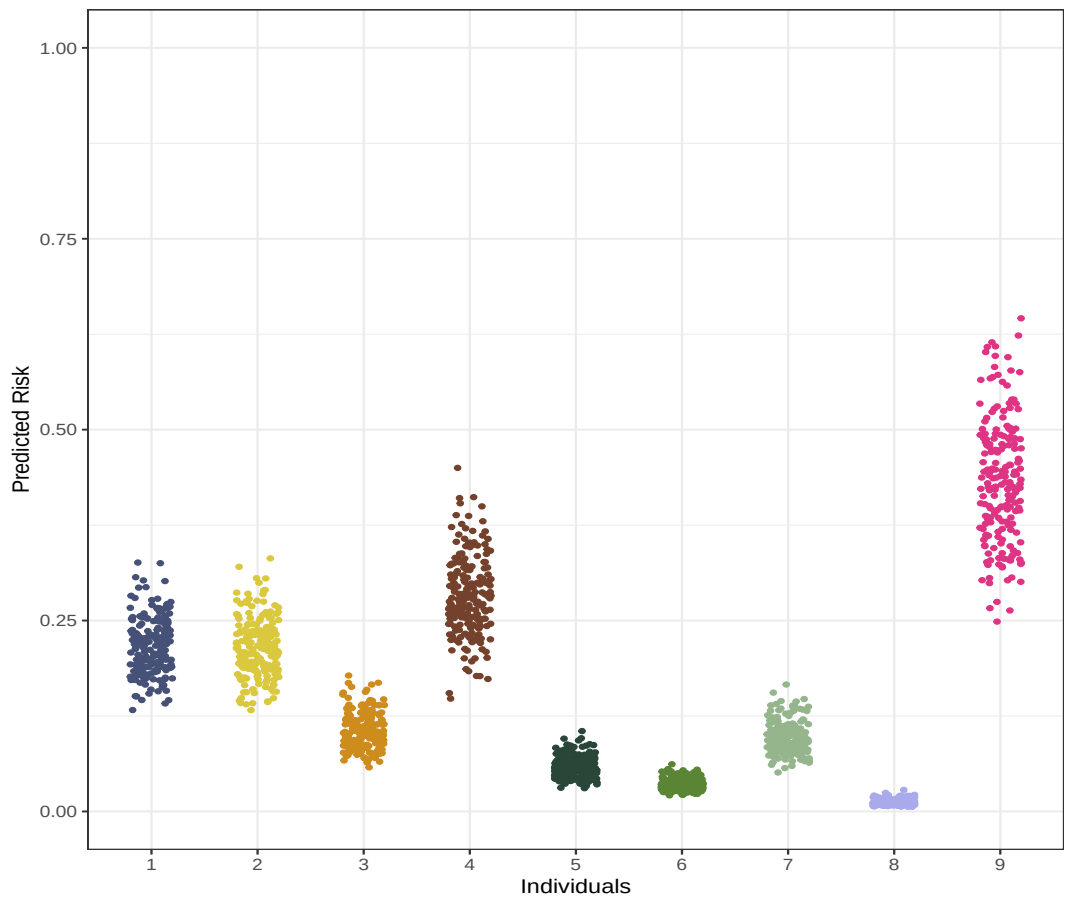

(c) Intercept Recalibration-ancillary only

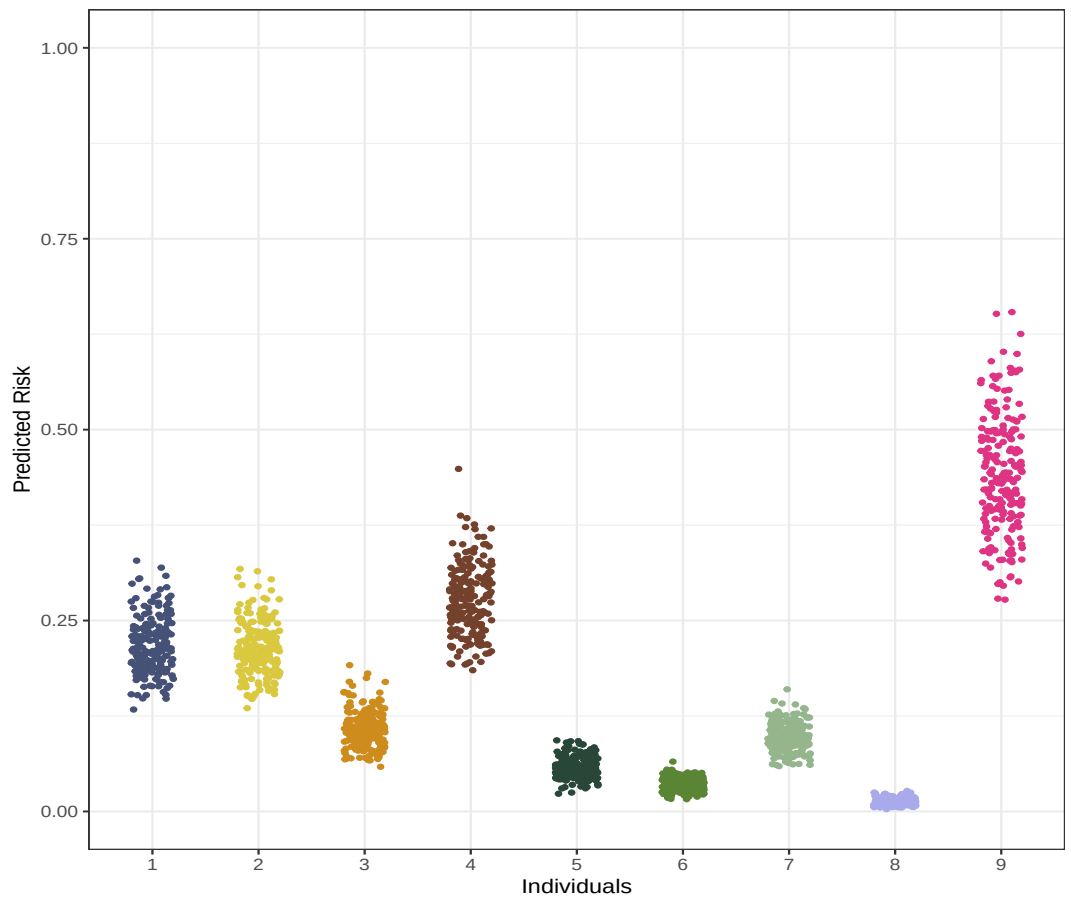

(d) Logistic Recalibration-all data

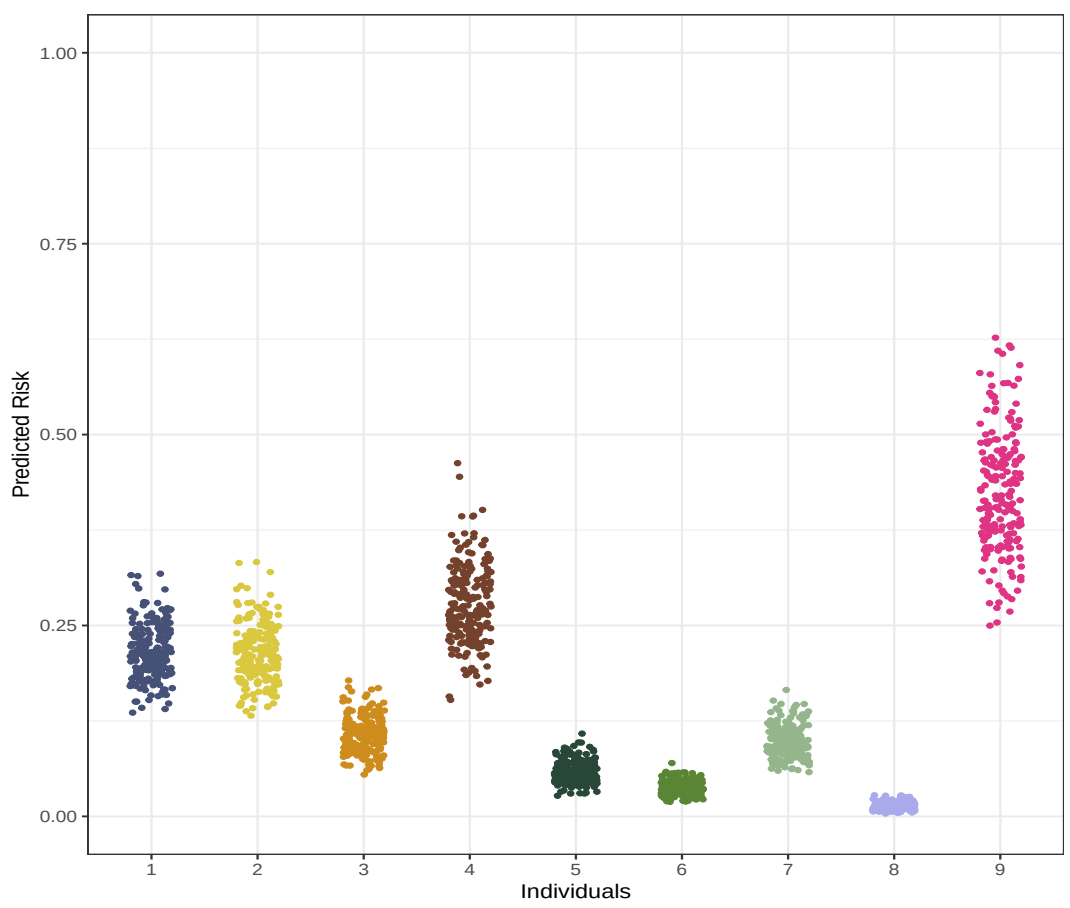

(e) Logistic Recalibration-ancillary only

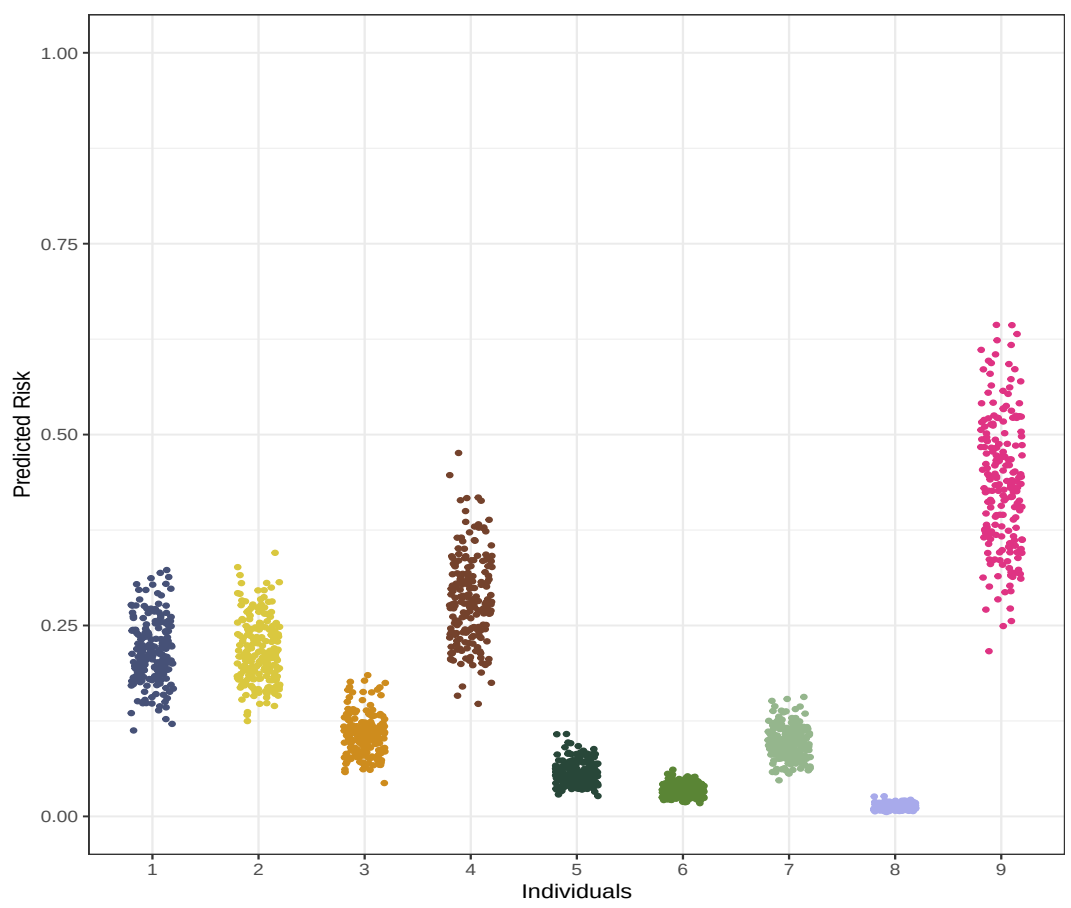

(f) Membership-based recalibration

Figure S11: Instability of estimated risks for nine individuals for low degree of single Case-mix Shift

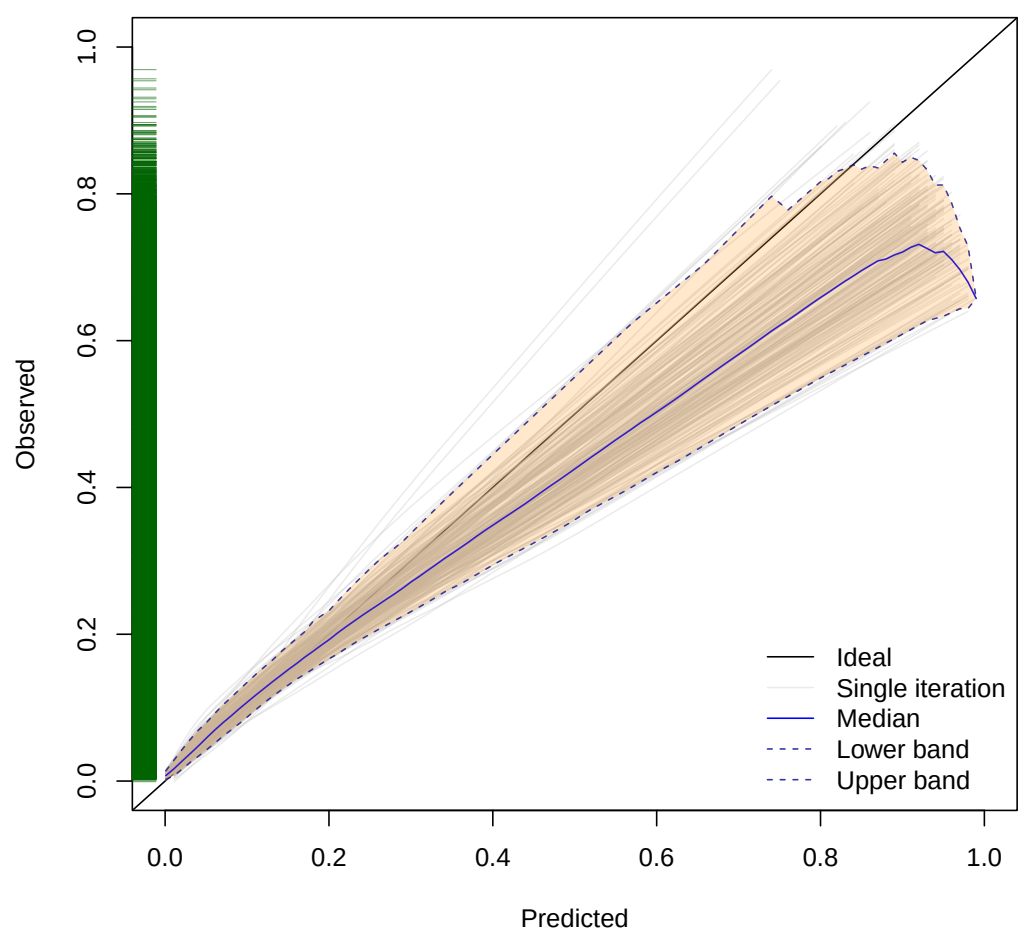

(a) Target-only model

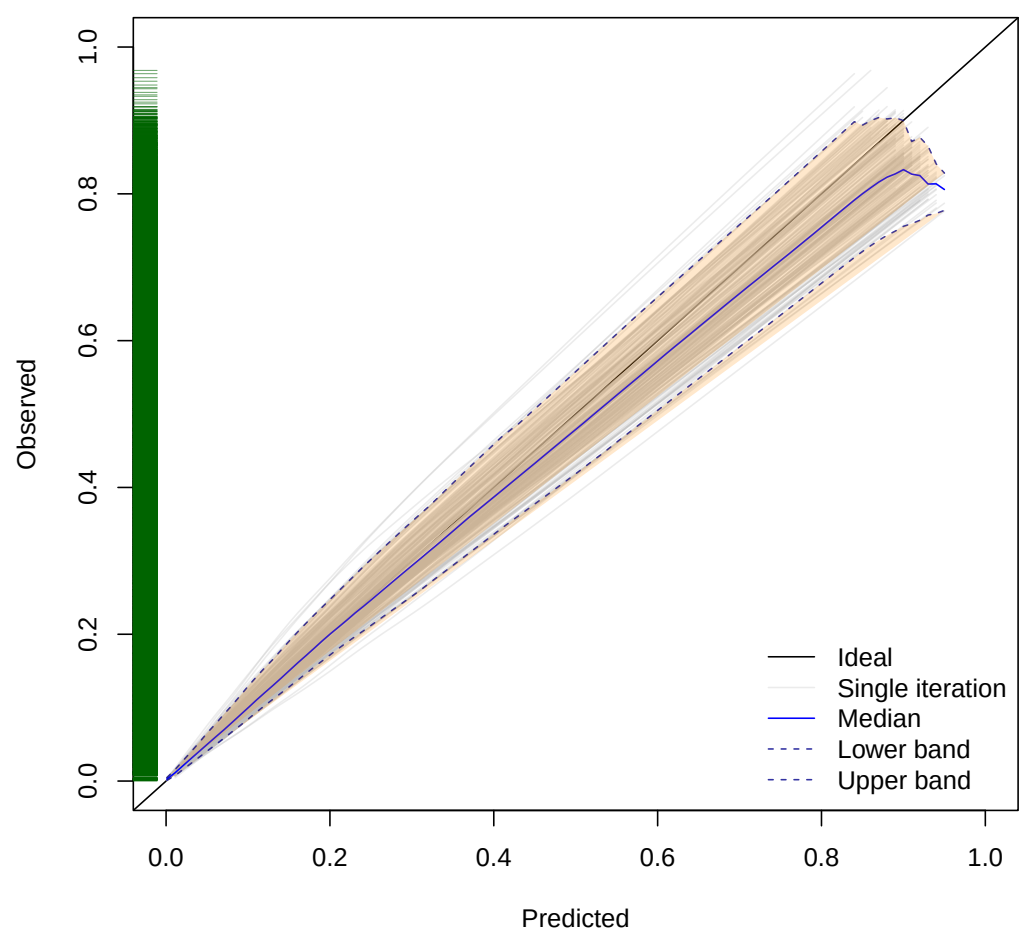

(b) Intercept Recalibration-all data

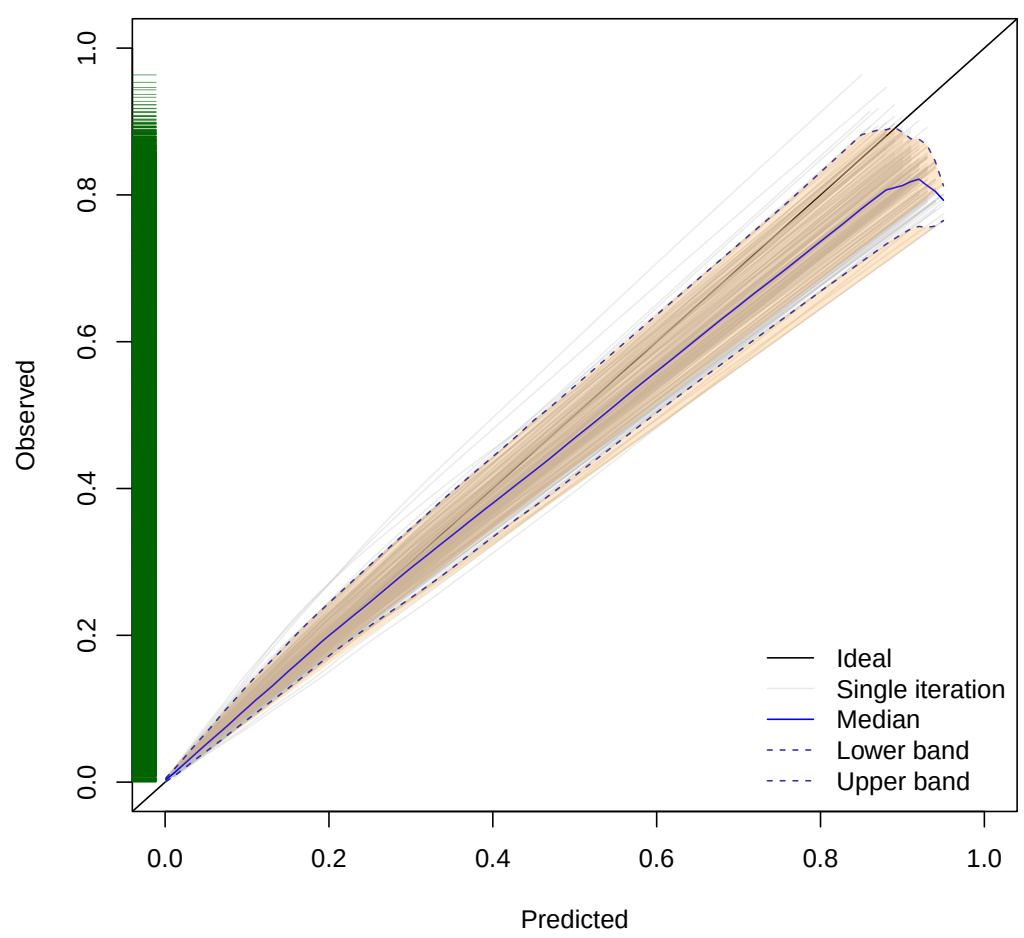

(c) Intercept Recalibration-ancillary only

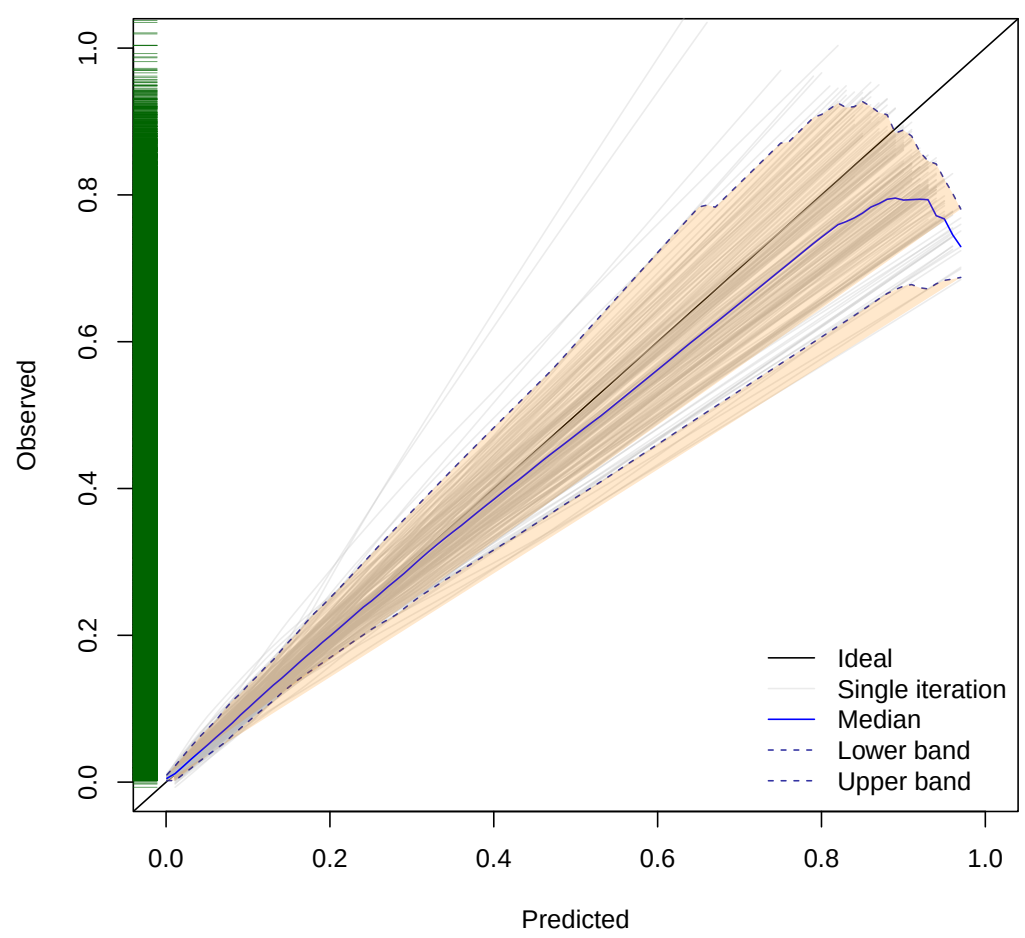

(d) Logistic Recalibration-all data

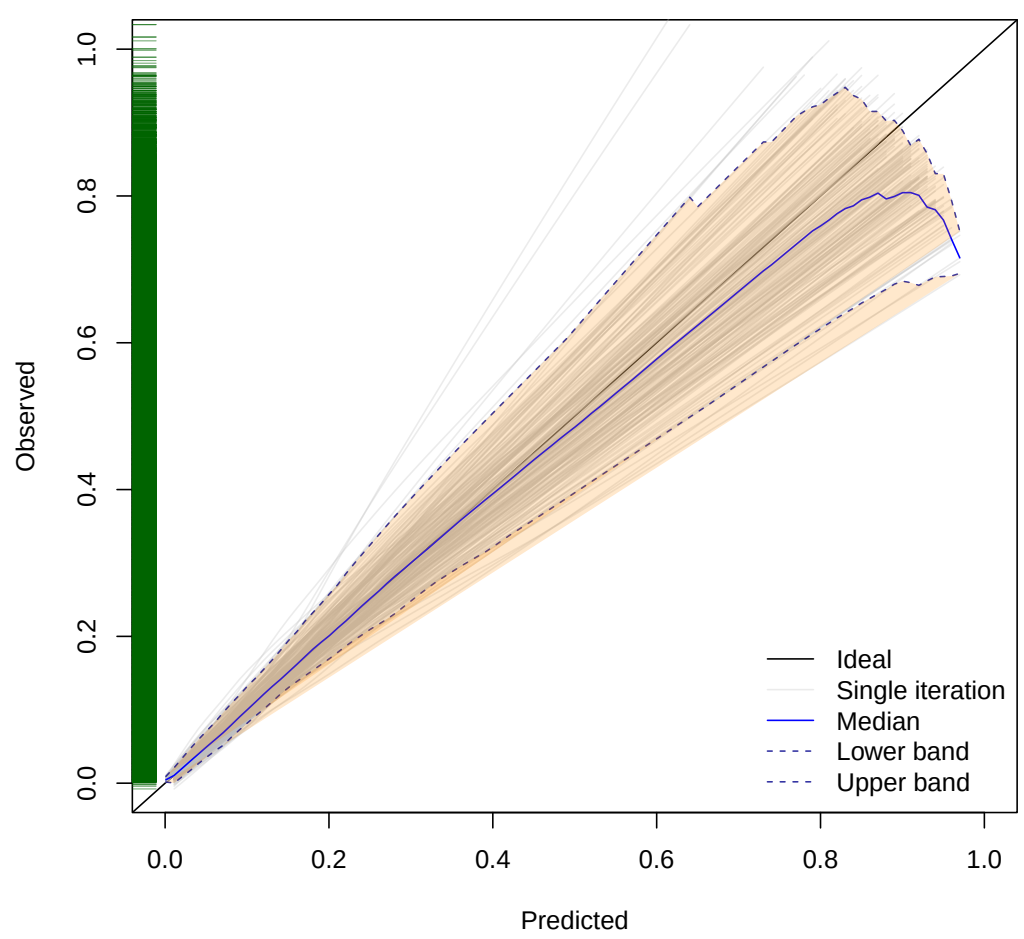

(e) Logistic Recalibration-ancillary only

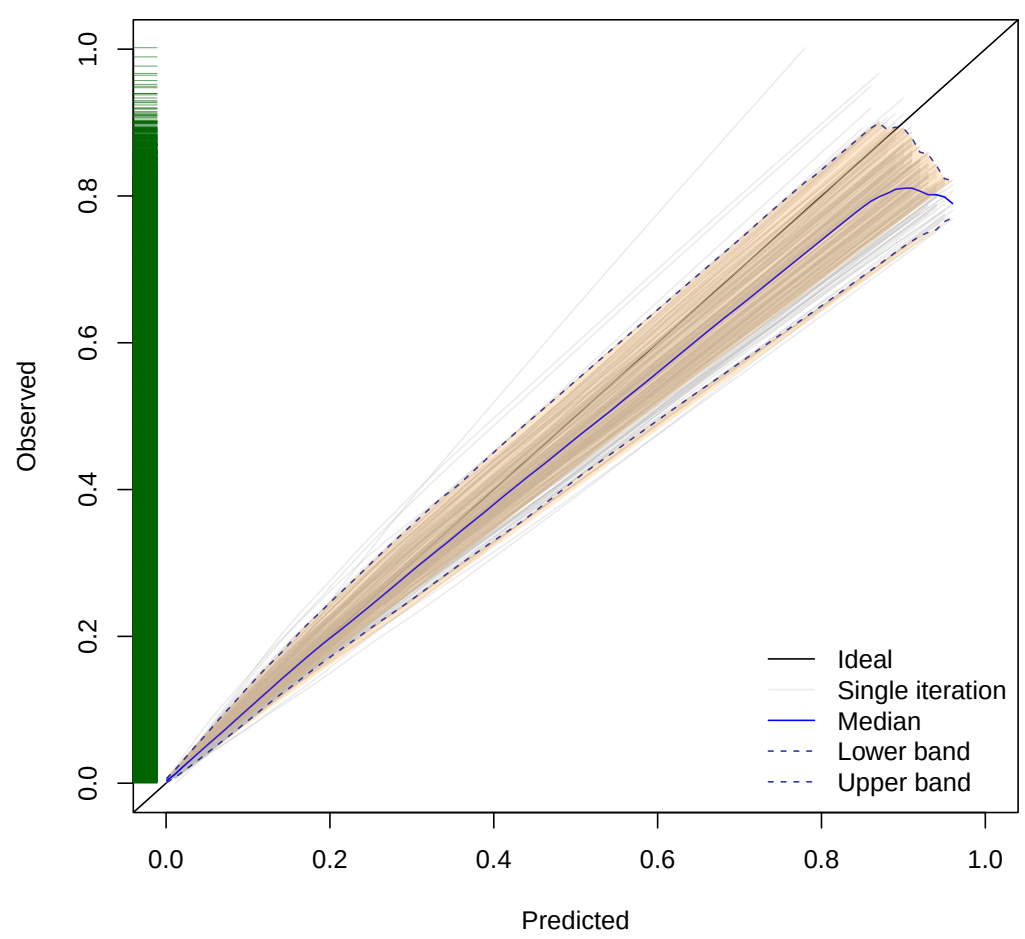

(f) Membership-based recalibration

Figure S12: Individual Calibration Plots for Event rate Shift

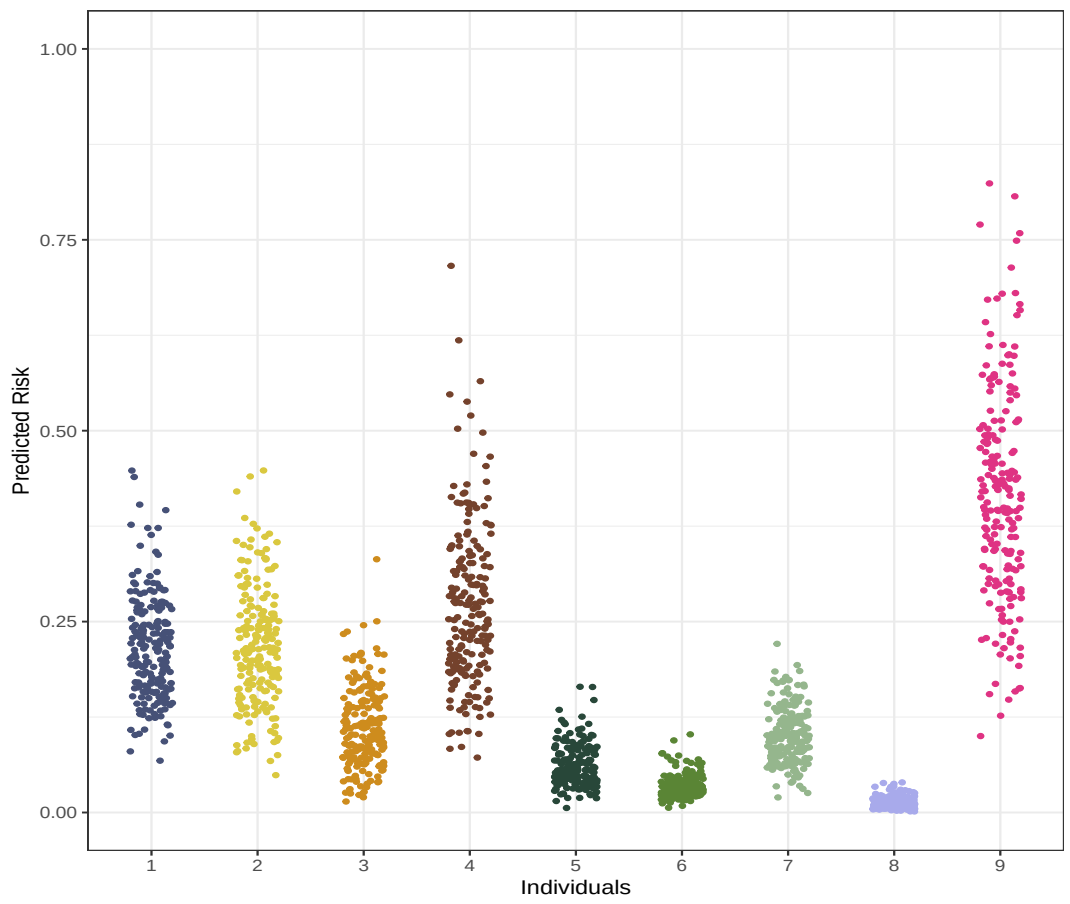

(a) Target-only

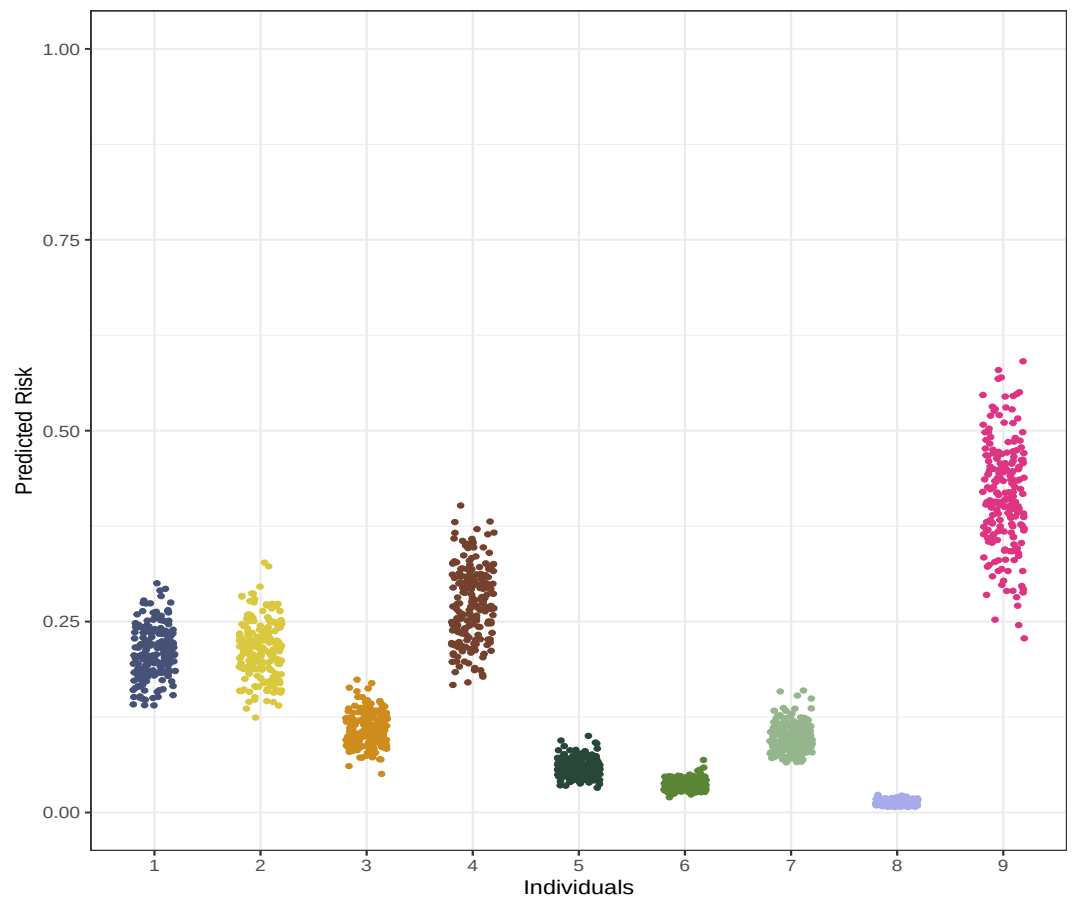

(b) Intercept Recalibration-all data

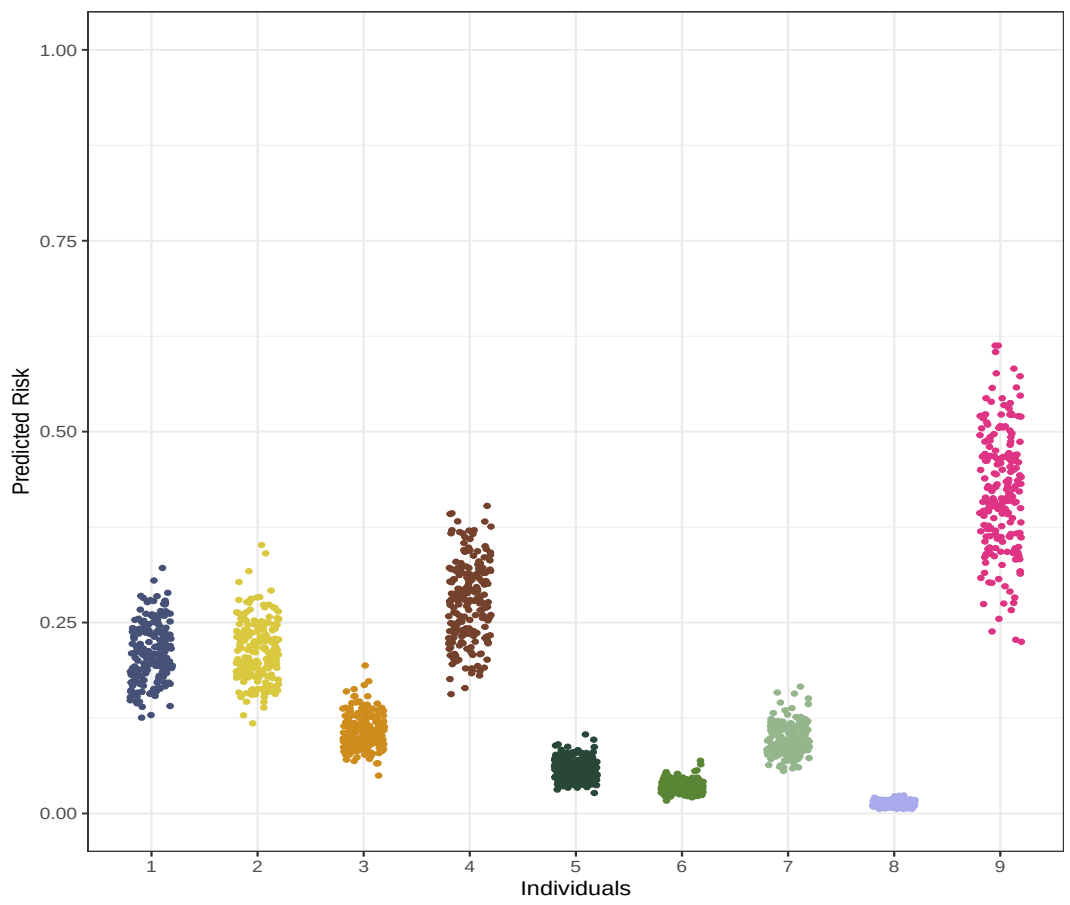

(c) Intercept Recalibration-ancillary only

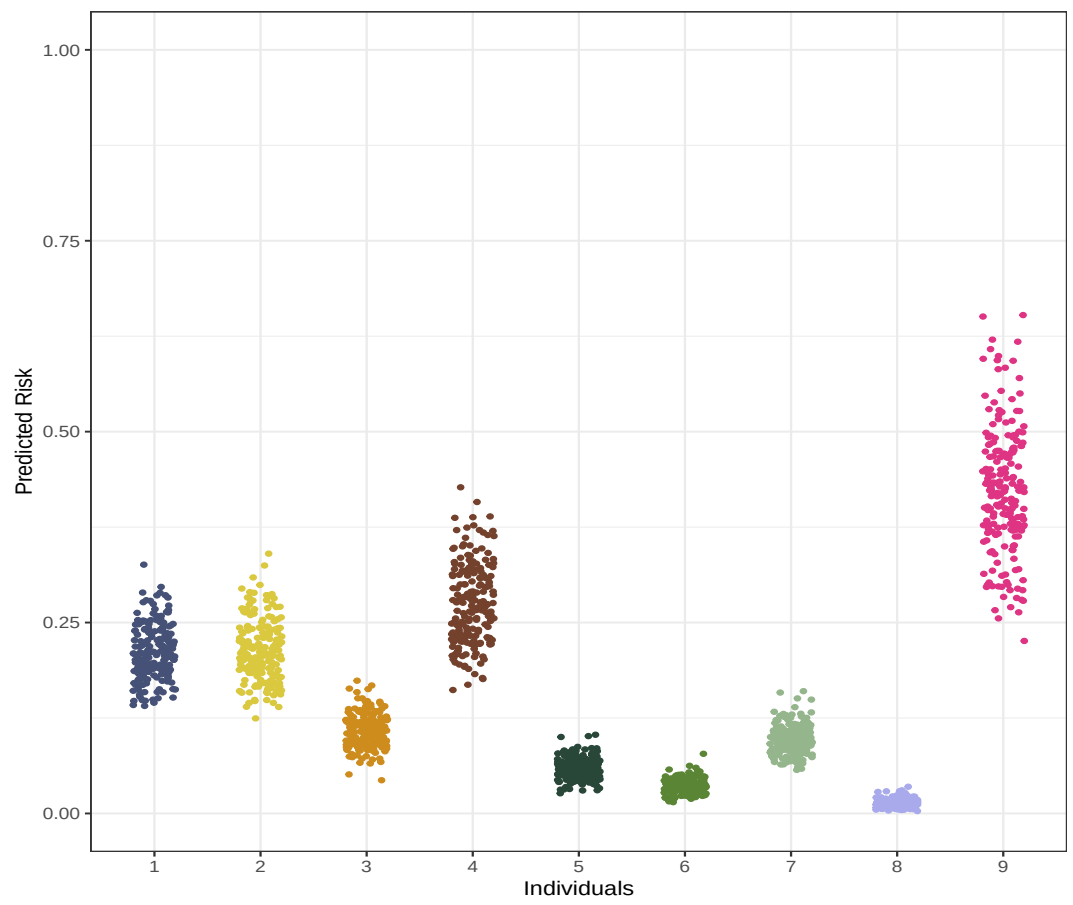

(d) Logistic Recalibration-all data

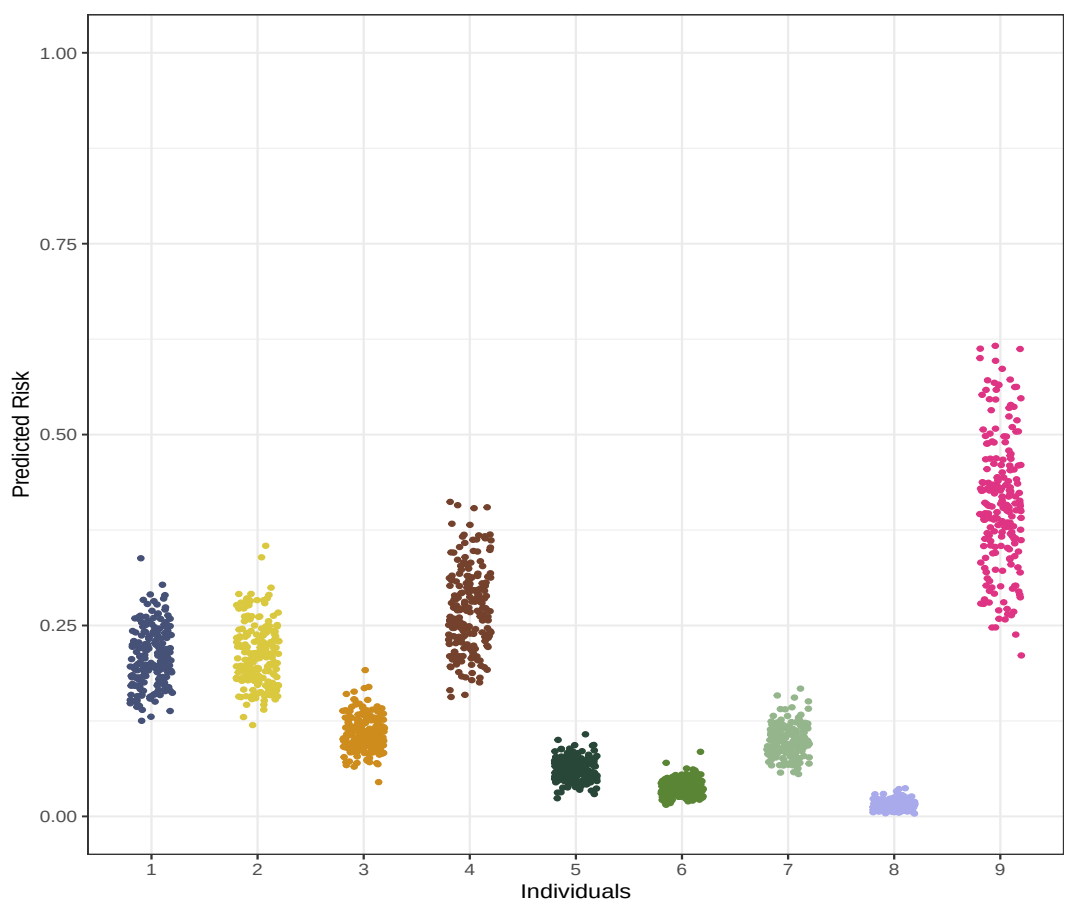

(e) Logistic Recalibration-ancillary only

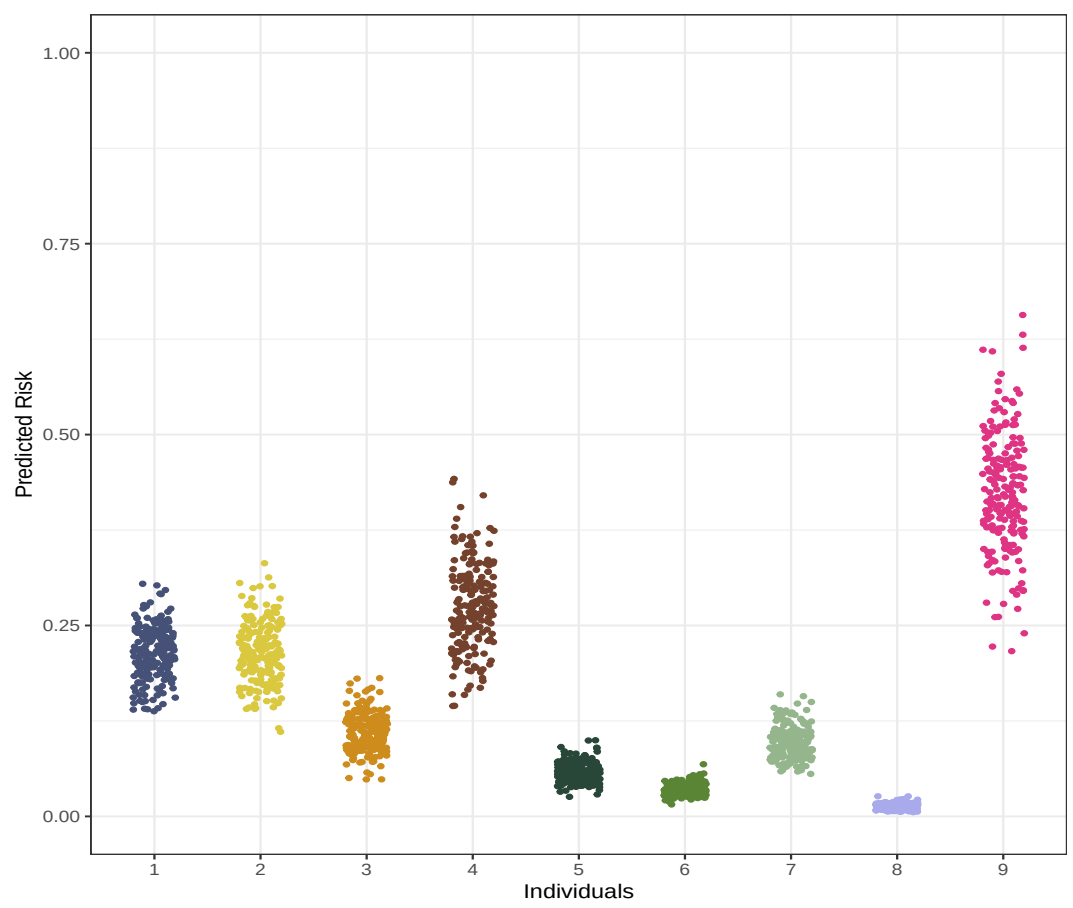

(f) Membership-based recalibration

Figure S13: Instability of estimated risks for nine individuals for Event rate Shift

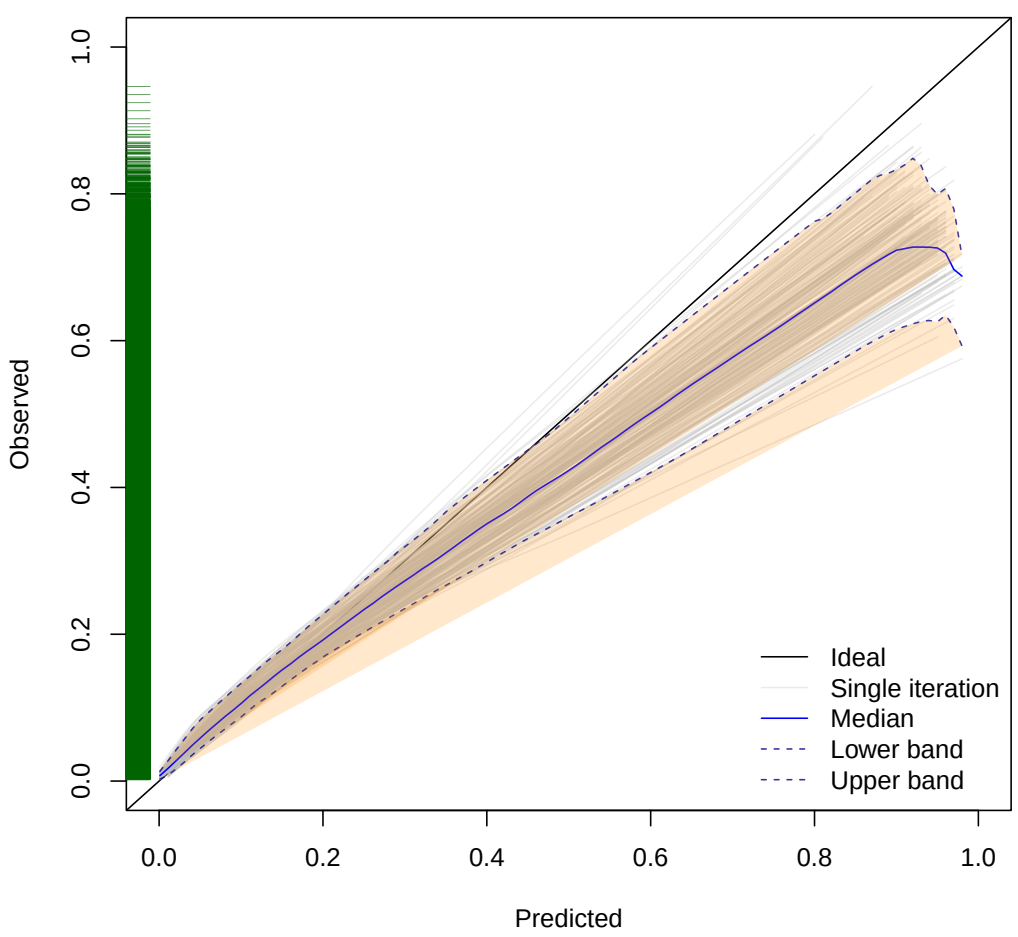

(a) Target-only model

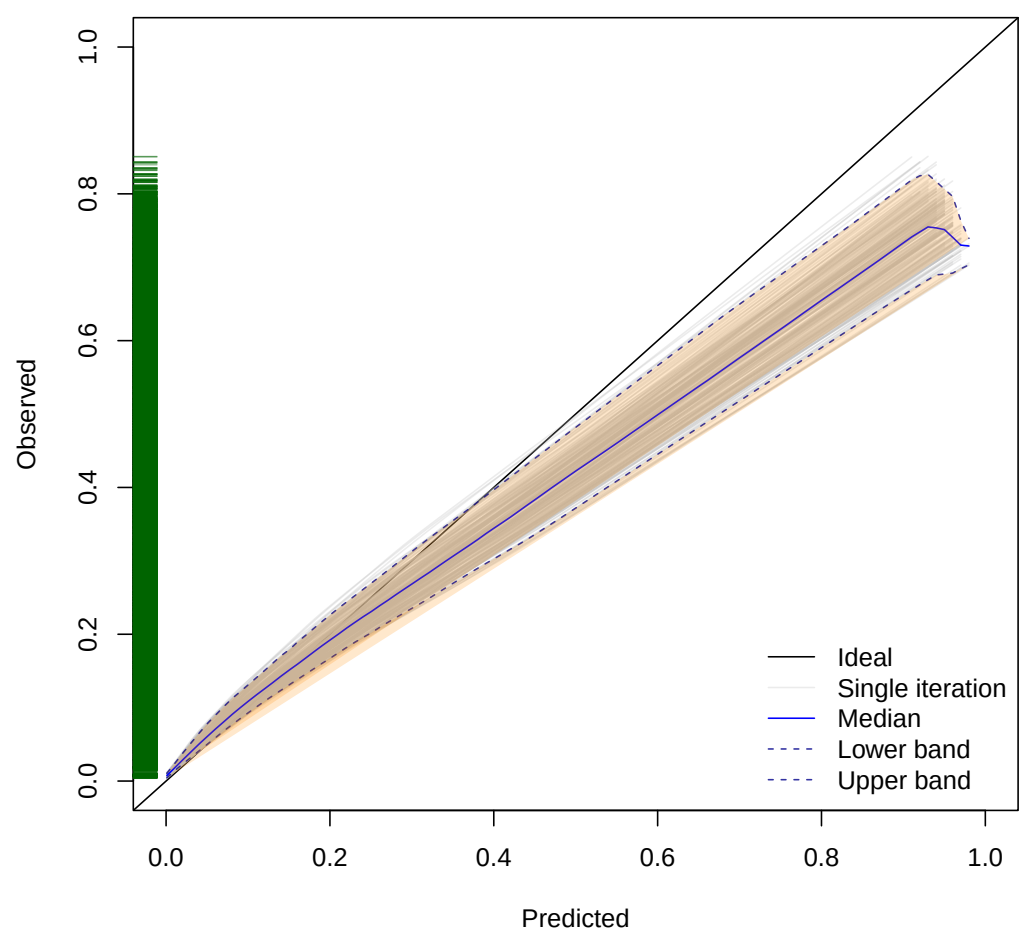

(b) Intercept Recalibration-all data

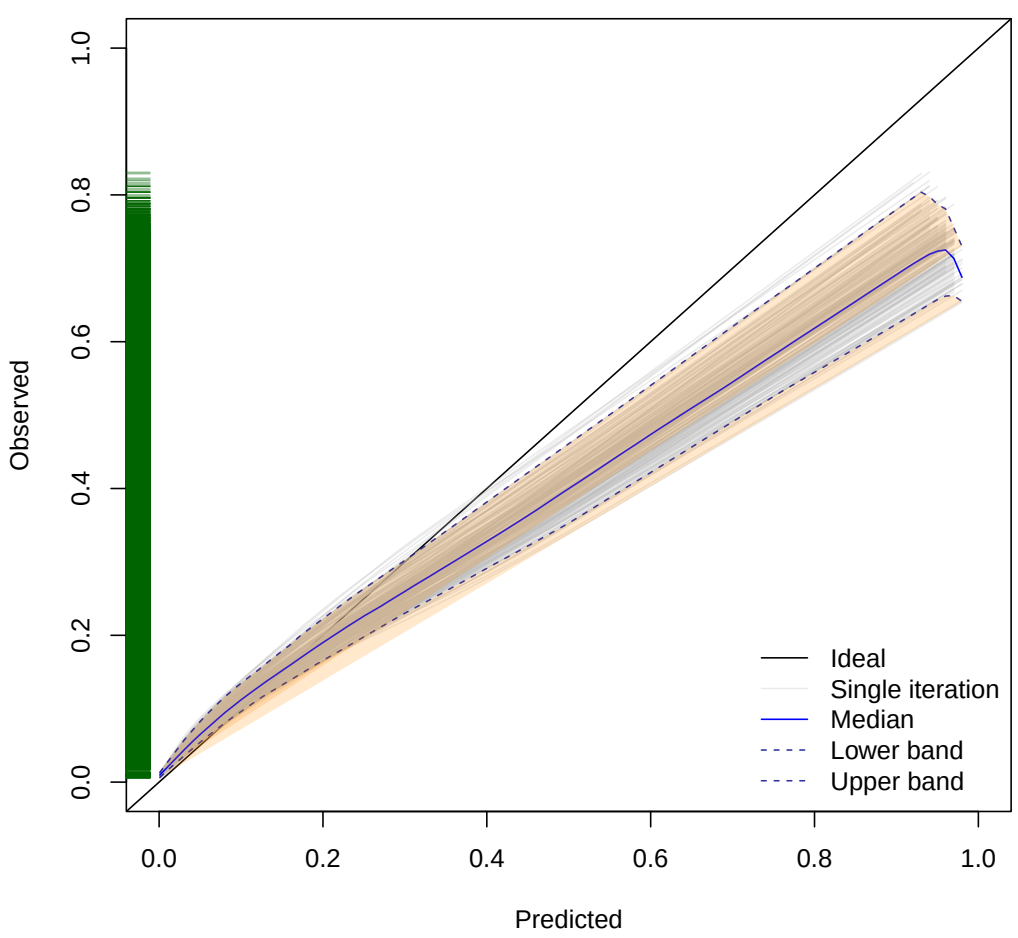

(c) Intercept Recalibration-ancillary only

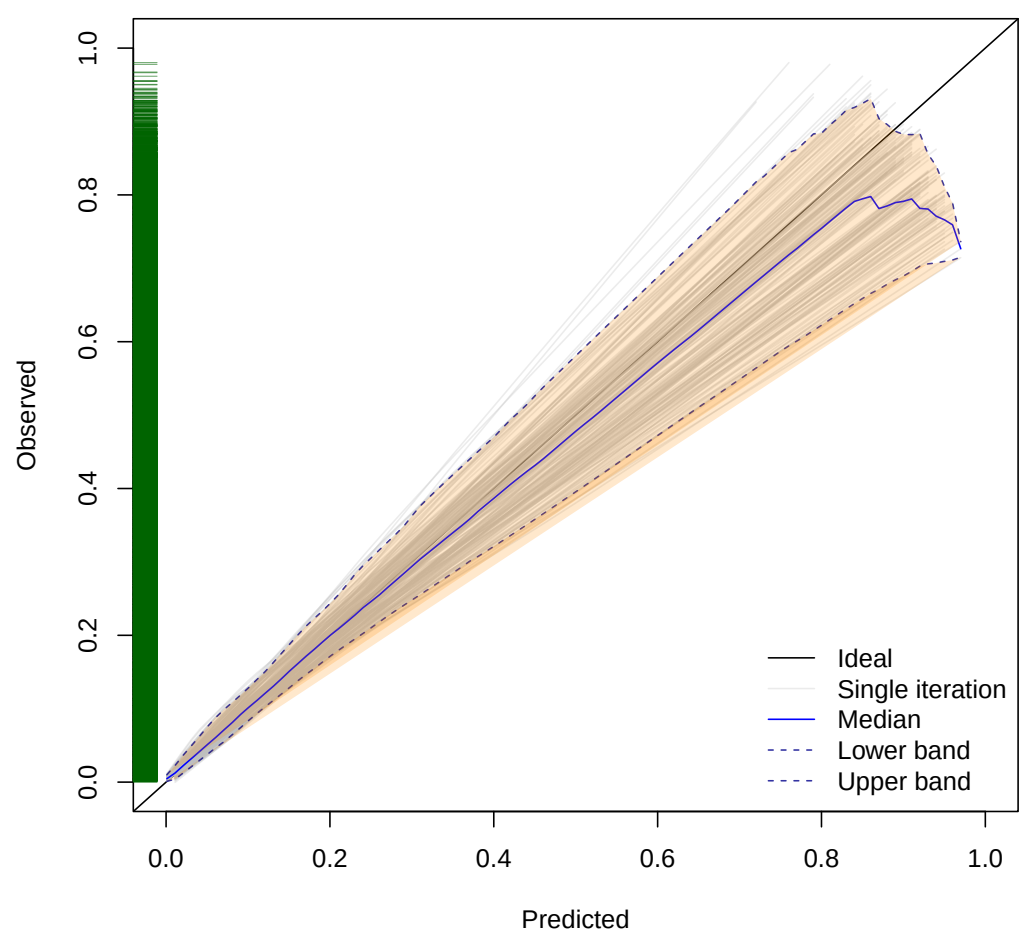

(d) Logistic Recalibration-all data

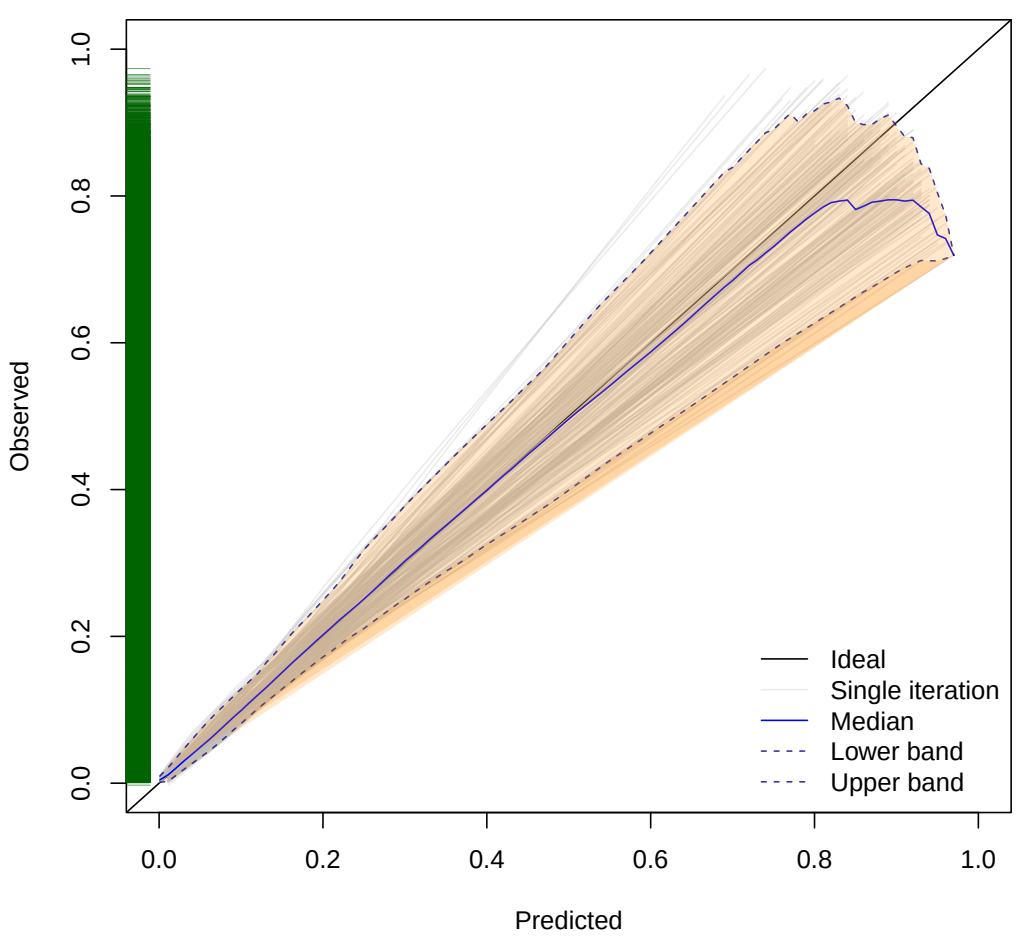

(e) Logistic Recalibration-ancillary only

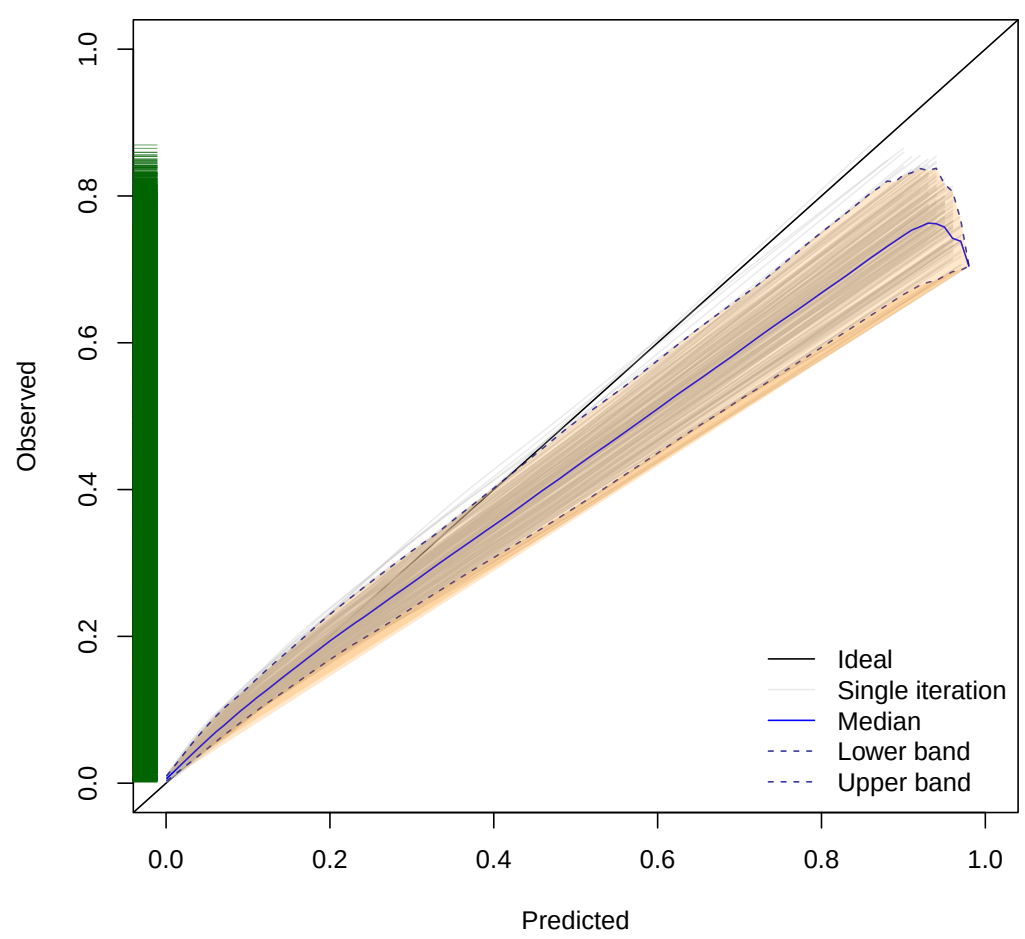

(f) Membership-based recalibration

Figure S14: Individual Calibration Plots for Predictor-outcome association Shift

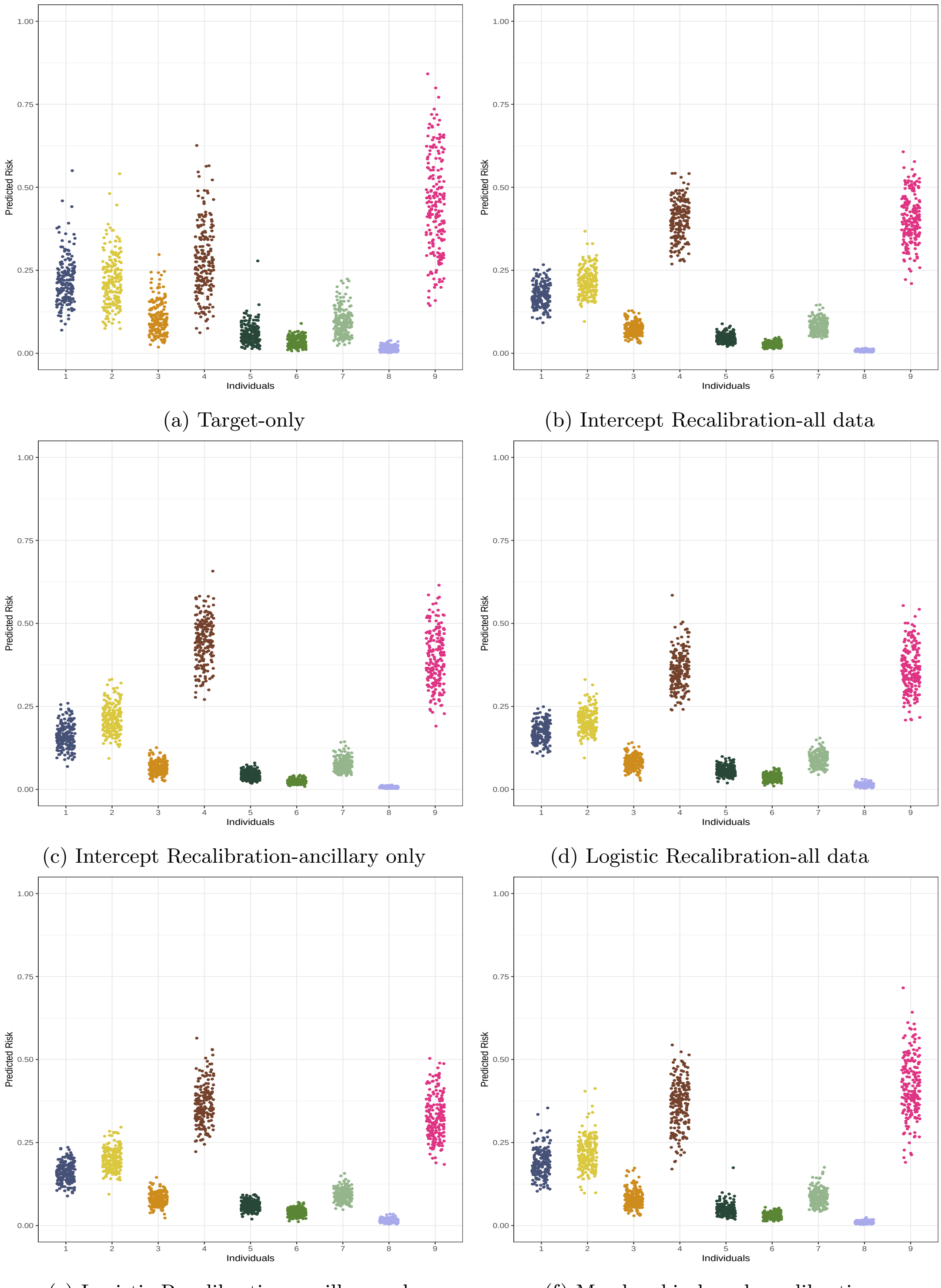

(a) Target-only

(b) Intercept Recalibration-all data

(c) Intercept Recalibration-ancillary only

(d) Logistic Recalibration-all data

(e) Logistic Recalibration-ancillary only

(f) Membership-based recalibration

Figure S15: Instability of estimated risks for nine individuals for Predictor-outcome association Shift

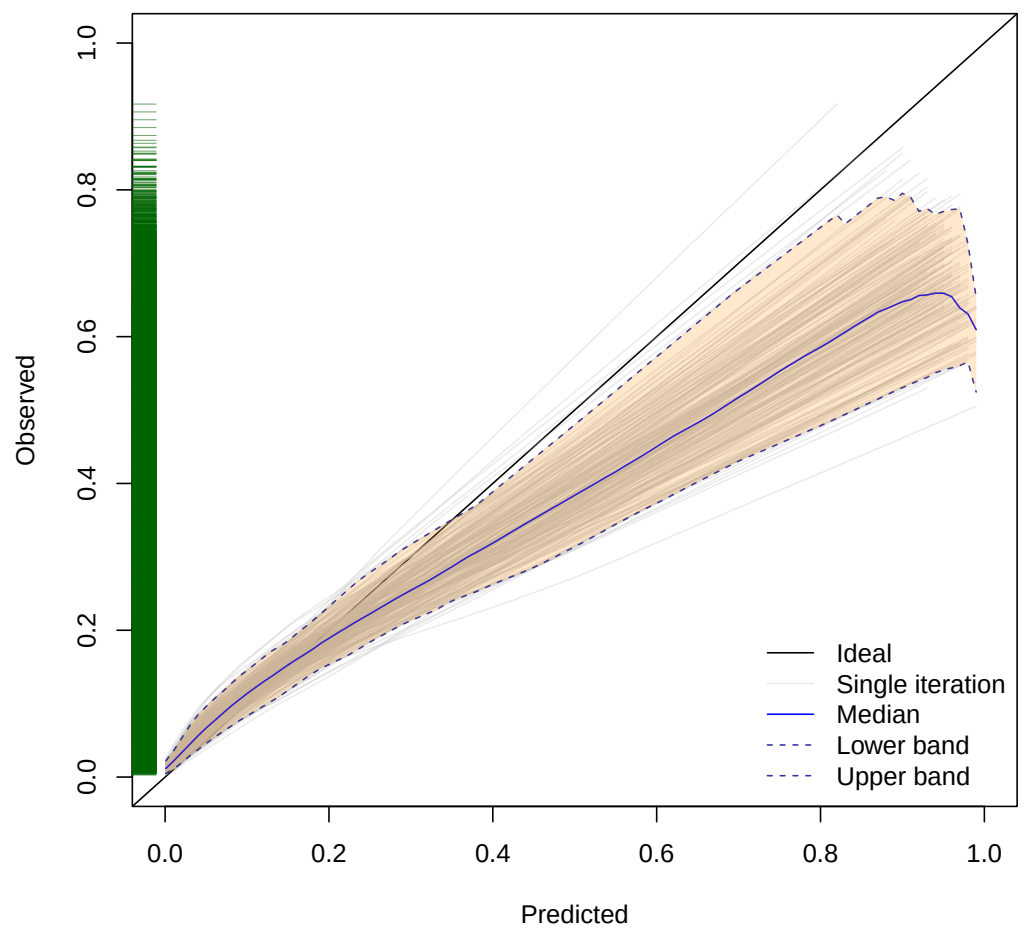

(a) Target-only model

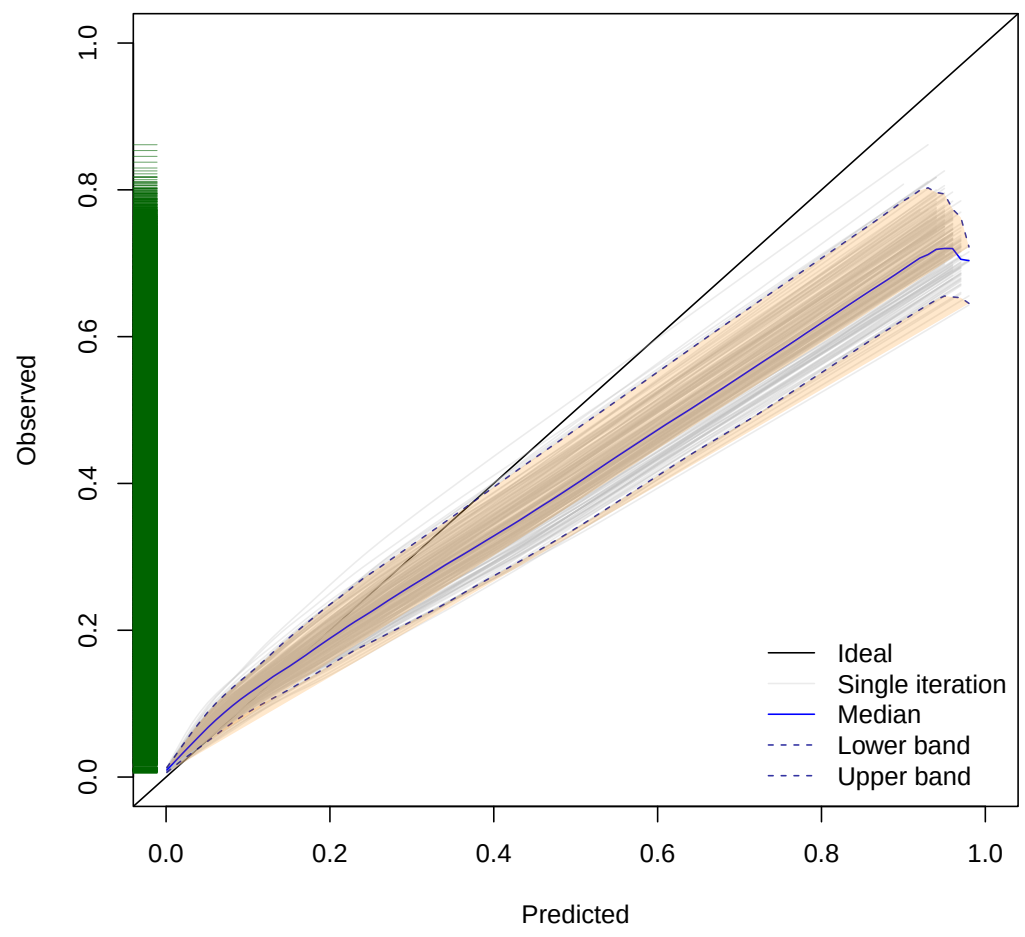

(b) Intercept Recalibration-all data

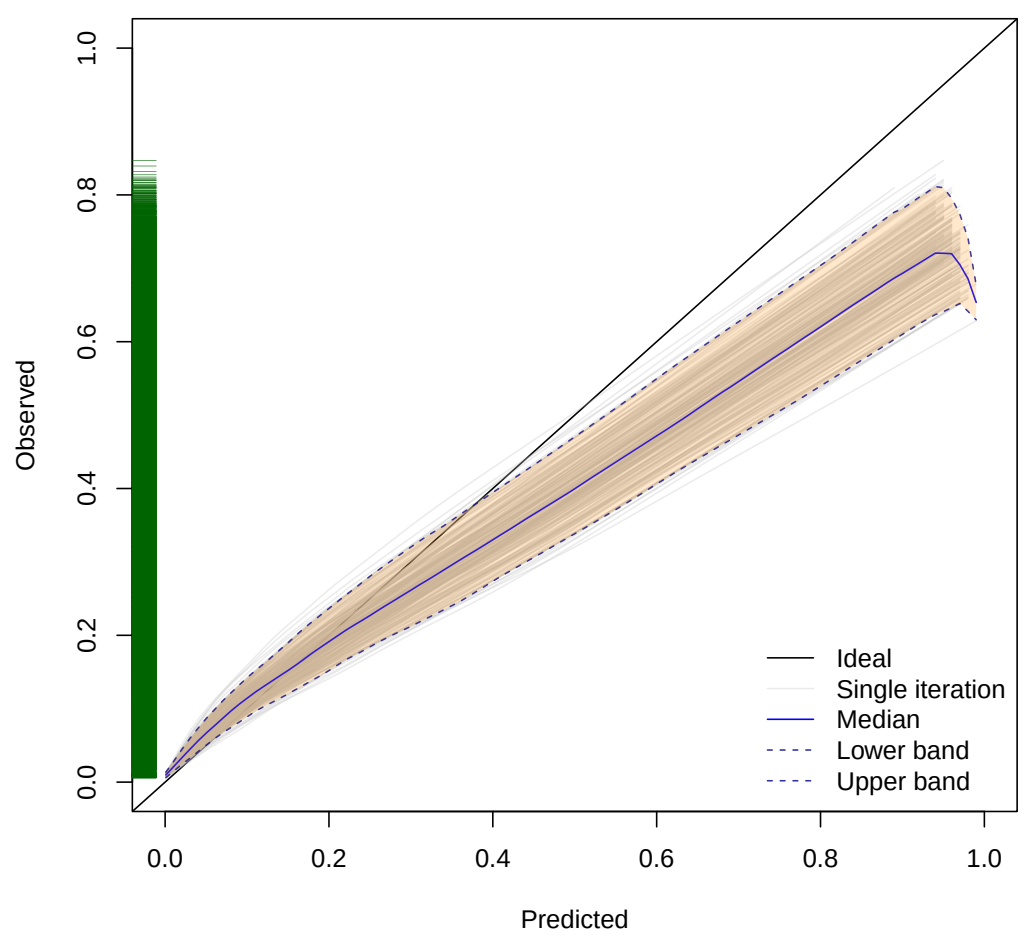

(c) Intercept Recalibration-ancillary only

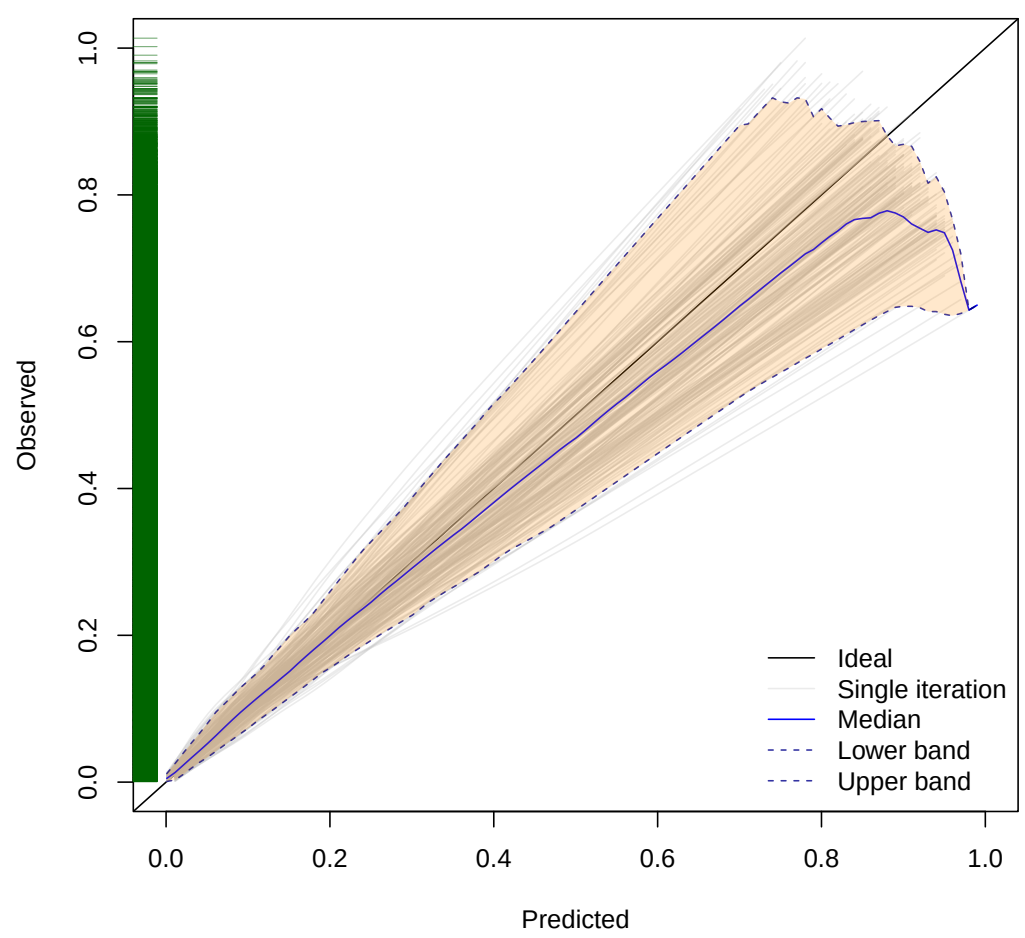

(d) Logistic Recalibration-all data

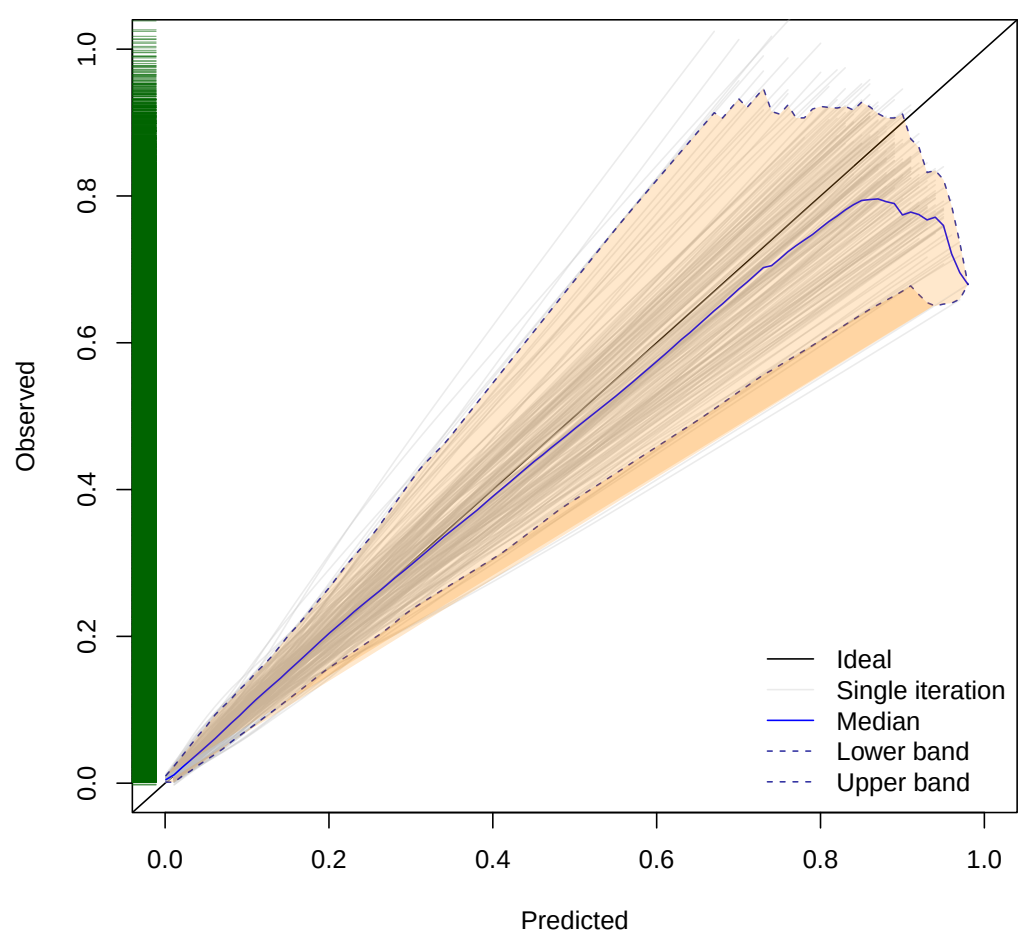

(e) Logistic Recalibration-ancillary only

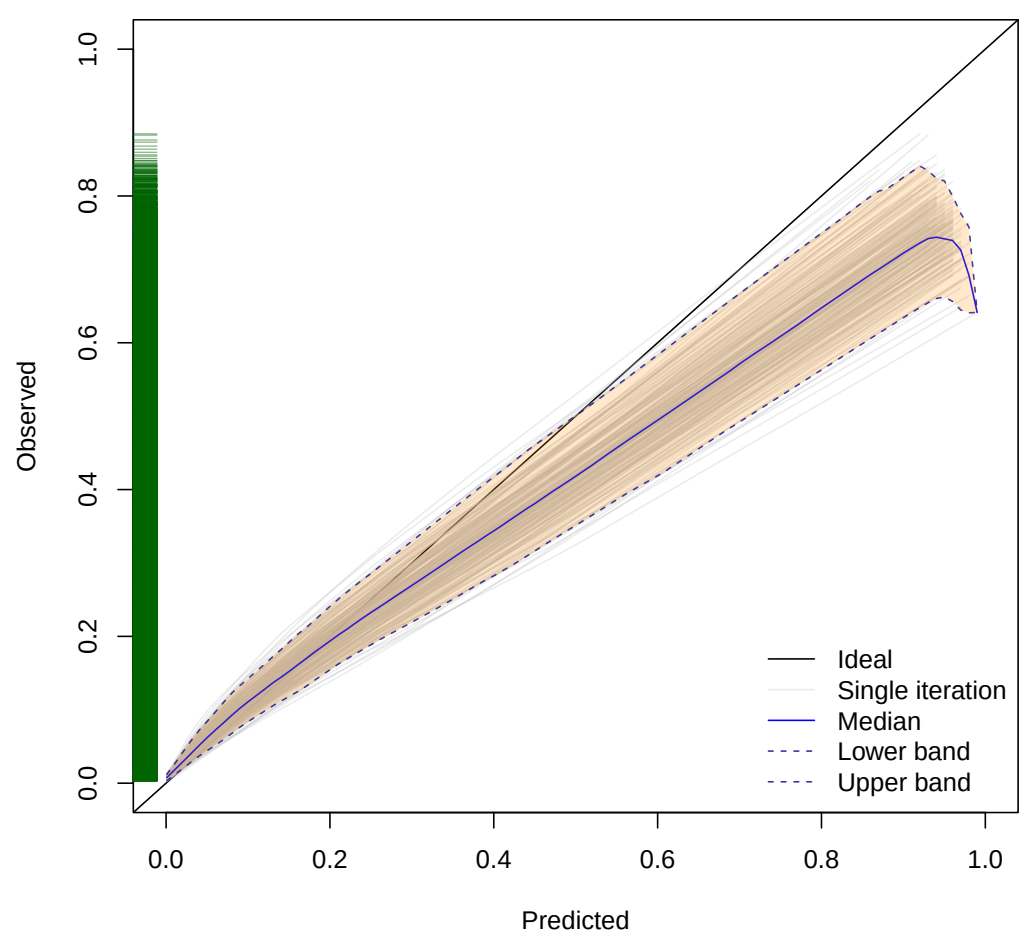

(f) Membership-based recalibration

Figure S16: Individual Calibration Plots for Combination of single Case-mix, Event rate and Predictor-outcome association shifts

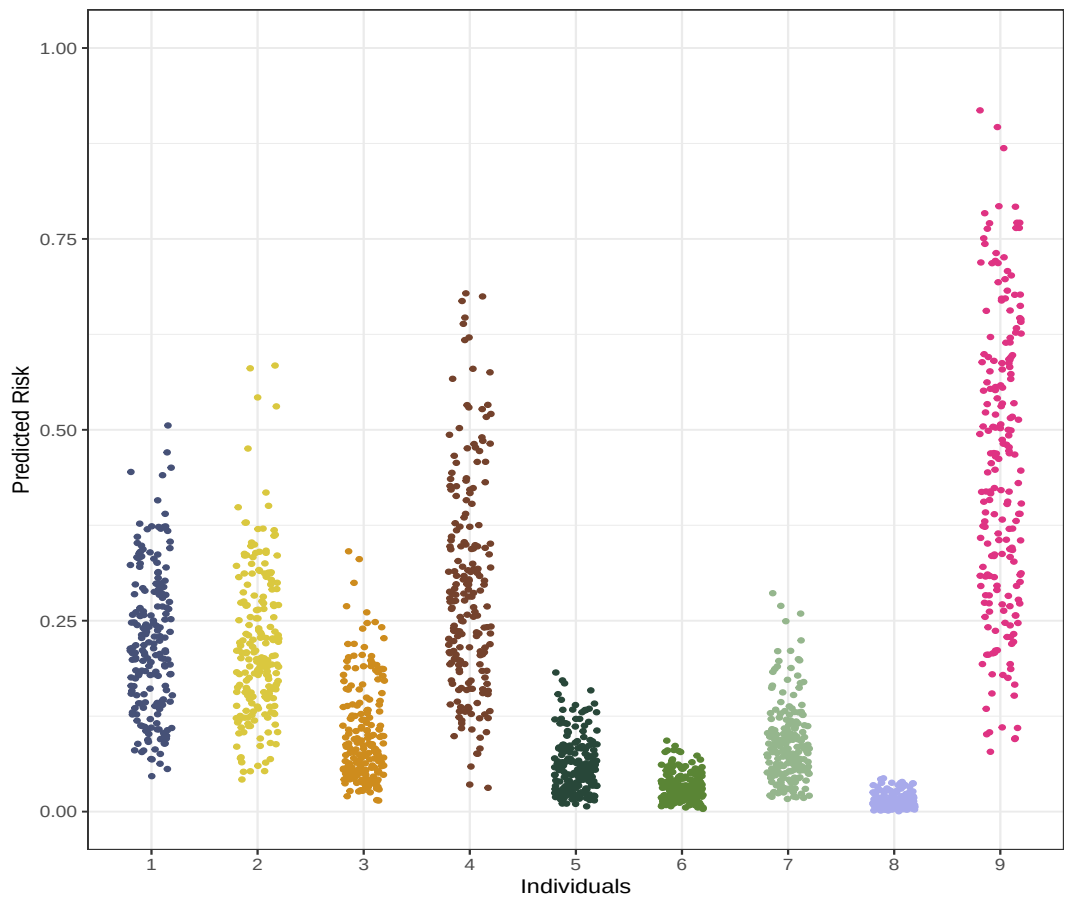

(a) Target-only

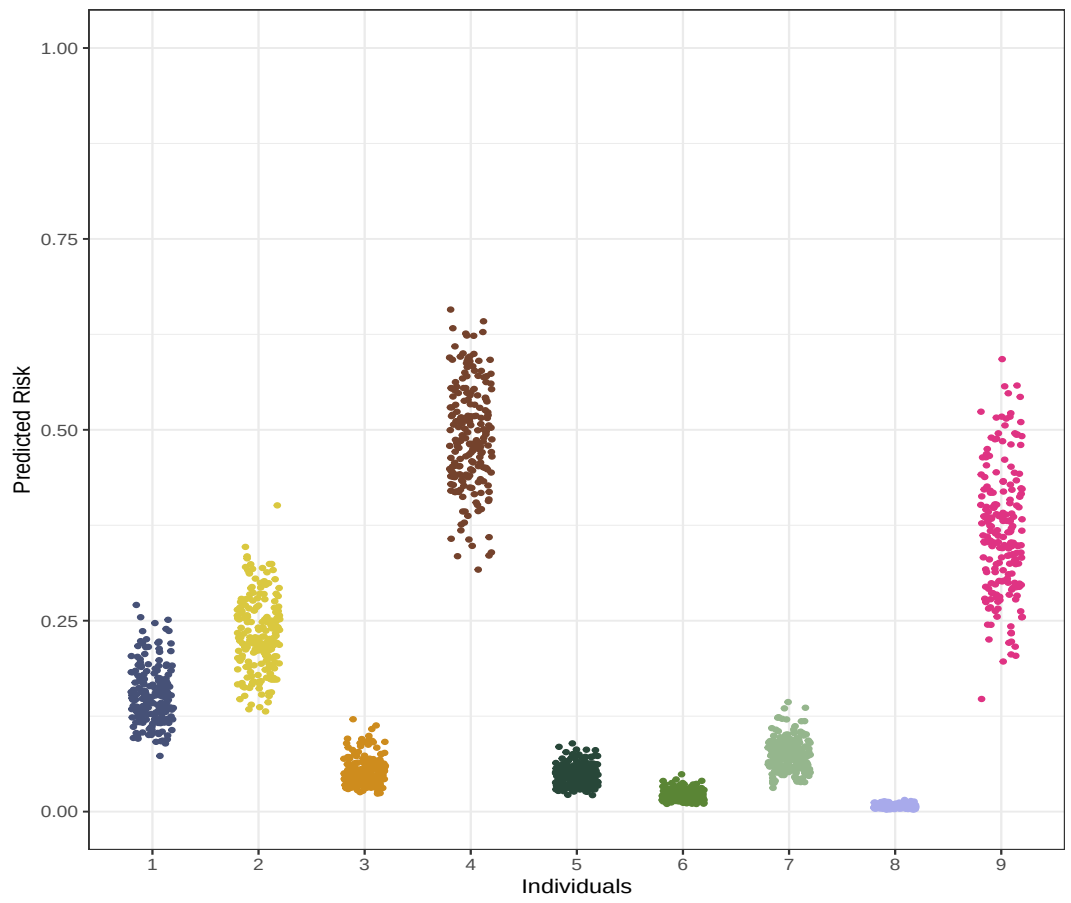

(b) Intercept Recalibration-all data

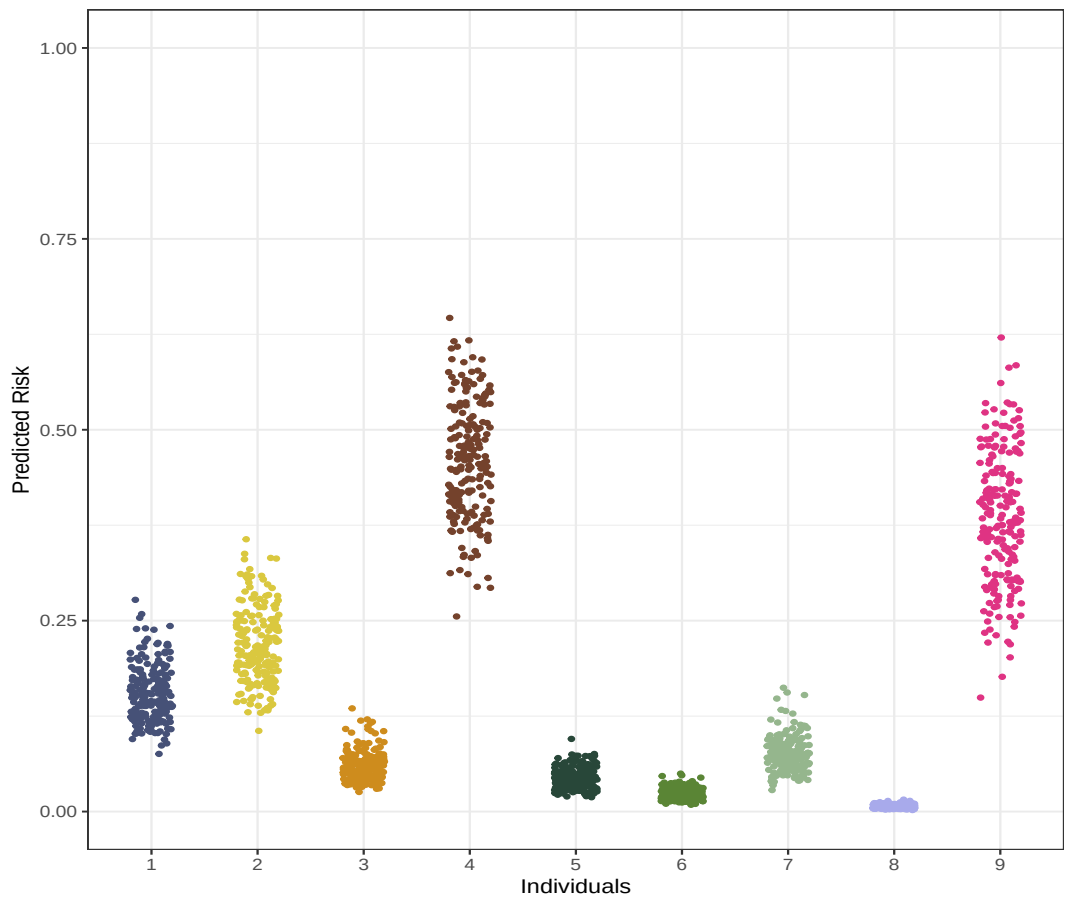

(c) Intercept Recalibration-ancillary only

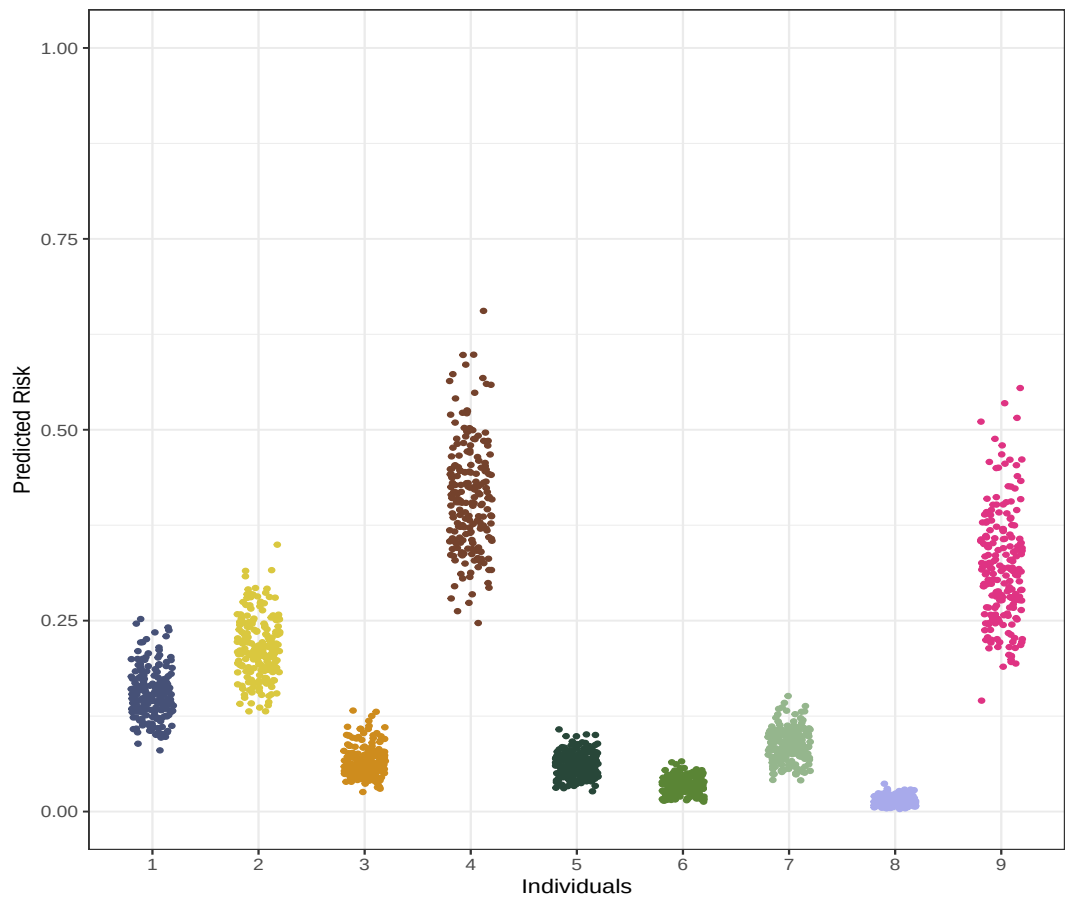

(d) Logistic Recalibration-all data

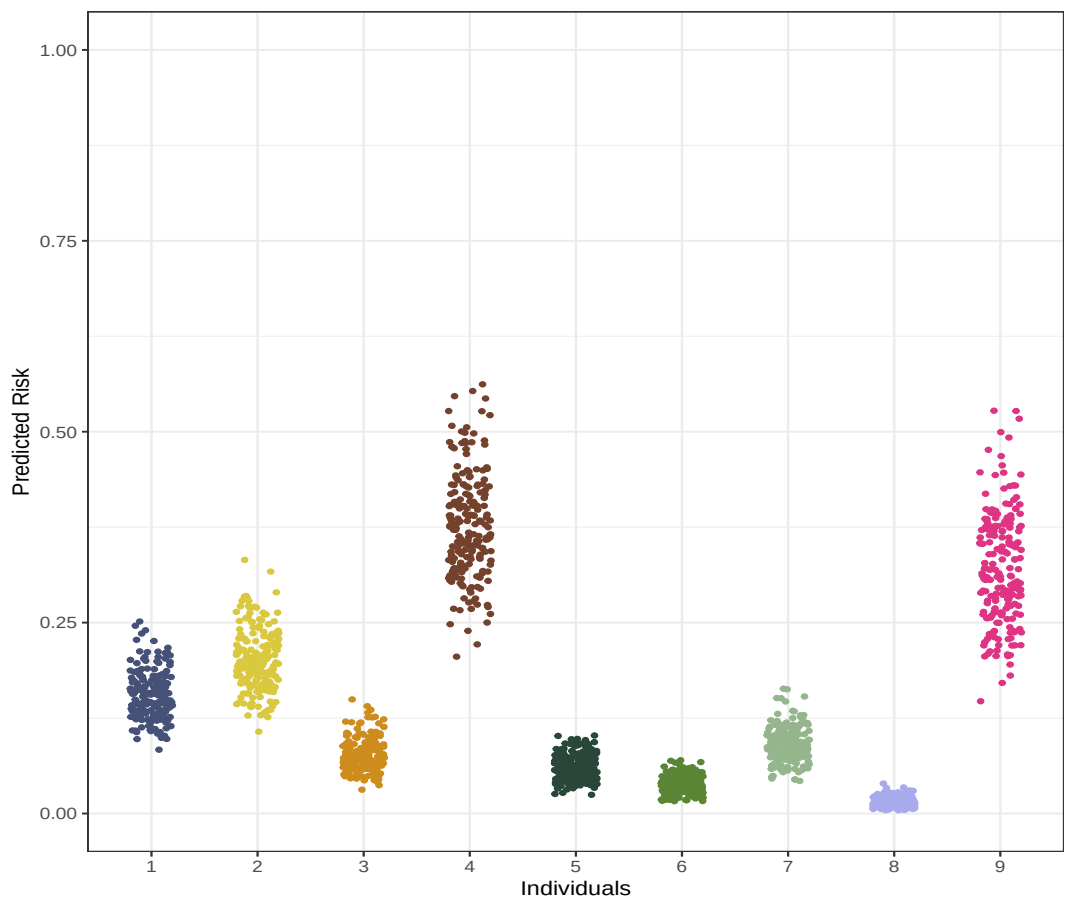

(e) Logistic Recalibration-ancillary only

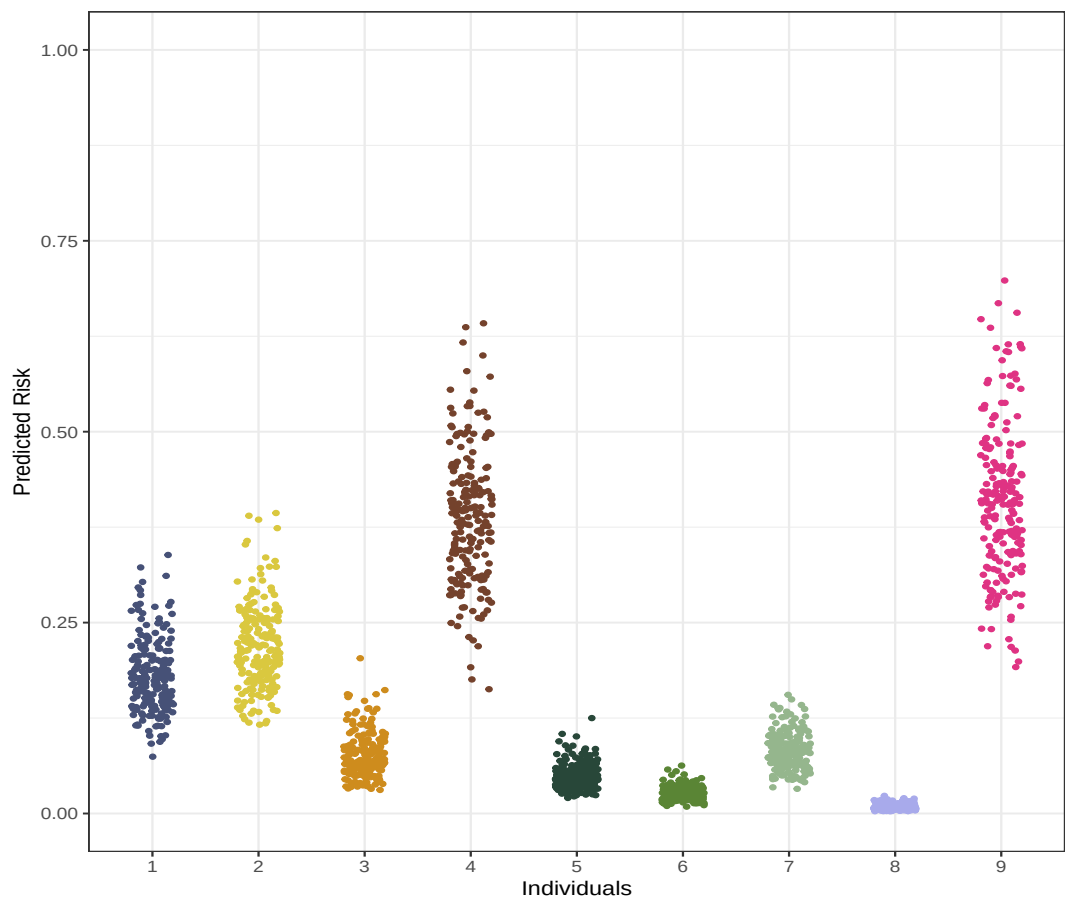

(f) Membership-based recalibration

Figure S17: Instability of estimated risks for nine individuals for Combination of single Case-mix, Event rate and Predictor-outcome association shifts

Table S4: Performance metrics for No shift

Model	AUC	CITL	CSLOPE	Brier Score
Membership-based recalibration	0.790(0.785,0.791)	-0.015(-0.188,0.186)	0.949(0.838,1.047)	0.083(0.082,0.084)
Target-only	0.781(0.770,0.788)	-0.013(-0.185,0.189)	0.870(0.700,1.044)	0.084(0.083,0.086)
Intercept Recalibration-all data	0.790(0.787,0.791)	-0.016(-0.188,0.186)	0.952(0.870,1.044)	0.083(0.082,0.083)
Intercept Recalibration-ancillary only	0.789(0.785,0.791)	-0.014(-0.182,0.182)	0.942(0.847,1.052)	0.083(0.082,0.083)
Logistic Recalibration-all data	0.790(0.787,0.791)	-0.016(-0.188,0.185)	0.948(0.748,1.184)	0.083(0.082,0.084)
Logistic Recalibration-ancillary only	0.789(0.785,0.791)	-0.015(-0.184,0.188)	0.981(0.773,1.229)	0.083(0.082,0.084)

Table S5: Performance metrics for low degree of single Case-mix shift

Model	AUC	CITL	CSLOPE	Brier Score
Membership-based recalibration	0.789(0.783,0.791)	0.000(-0.236,0.204)	0.943(0.817,1.042)	0.083(0.082,0.084)
Target-only	0.780(0.765,0.788)	0.001(-0.234,0.213)	0.854(0.670,1.021)	0.084(0.083,0.087)
Intercept Recalibration-all data	0.790(0.787,0.792)	0.001(-0.237,0.201)	0.958(0.893,1.050)	0.083(0.082,0.083)
Intercept Recalibration-ancillary only	0.789(0.785,0.792)	-0.001(-0.237,0.199)	0.947(0.858,1.062)	0.083(0.082,0.083)
Logistic Recalibration-all data	0.790(0.787,0.792)	-0.001(-0.239,0.203)	0.948(0.772,1.139)	0.083(0.082,0.084)
Logistic Recalibration-ancillary only	0.789(0.785,0.792)	-0.002(-0.239,0.204)	0.973(0.799,1.198)	0.083(0.082,0.084)

Table S6: Performance metrics for high degree of single Case-mix shift

Model	AUC	CITL	CSLOPE	Brier Score
Membership-based recalibration	0.767(0.737,0.782)	-0.007(-0.343,0.400)	0.748(0.561,0.970)	0.087(0.084,0.091)
Target-only	0.767(0.737,0.782)	-0.007(-0.343,0.400)	0.748(0.561,0.970)	0.087(0.084,0.091)
Intercept Recalibration-all data	0.789(0.784,0.791)	0.005(-0.326,0.374)	0.969(0.860,1.091)	0.083(0.082,0.084)
Intercept Recalibration-ancillary only	0.788(0.784,0.791)	0.006(-0.328,0.371)	0.942(0.837,1.081)	0.083(0.083,0.084)
Logistic Recalibration-all data	0.789(0.784,0.791)	0.005(-0.343,0.379)	0.959(0.728,1.252)	0.083(0.082,0.085)
Logistic Recalibration-ancillary only	0.788(0.784,0.791)	0.005(-0.333,0.371)	0.997(0.752,1.341)	0.083(0.082,0.085)

Table S7: Performance metrics for Event rate shift

Model	AUC	CITL	CSLOPE	Brier Score
Membership-based recalibration	0.790(0.782,0.792)	-0.017(-0.223,0.271)	0.949(0.830,1.058)	0.083(0.082,0.084)
Target-only	0.778(0.761,0.787)	-0.019(-0.238,0.288)	0.840(0.654,1.060)	0.085(0.083,0.087)
Intercept Recalibration-all data	0.790(0.787,0.792)	-0.019(-0.223,0.258)	0.976(0.892,1.073)	0.083(0.082,0.083)
Intercept Recalibration-ancillary only	0.790(0.785,0.792)	-0.014(-0.216,0.271)	0.949(0.859,1.051)	0.083(0.082,0.084)
Logistic Recalibration-all data	0.790(0.787,0.792)	-0.019(-0.227,0.273)	0.968(0.752,1.221)	0.083(0.082,0.084)
Logistic Recalibration-ancillary only	0.790(0.785,0.792)	-0.014(-0.224,0.275)	0.993(0.773,1.264)	0.083(0.082,0.085)

Table S8: Performance metrics for Predictor-outcome association shift ($\Delta\beta_1 = 0.4$)

Model	AUC	CITL	CSLOPE	Brier Score
Membership-based recalibration	0.787(0.779,0.790)	-0.015(-0.220,0.250)	0.829(0.731,0.939)	0.084(0.083,0.085)
Target-only	0.777(0.756,0.787)	-0.016(-0.219,0.258)	0.826(0.664,1.020)	0.085(0.083,0.088)
Intercept Recalibration-all data	0.787(0.781,0.790)	-0.005(-0.222,0.244)	0.807(0.722,0.896)	0.084(0.083,0.085)
Intercept Recalibration-ancillary only	0.784(0.777,0.789)	-0.003(-0.235,0.253)	0.749(0.662,0.837)	0.084(0.083,0.086)
Logistic Recalibration-all data	0.787(0.781,0.790)	-0.014(-0.202,0.237)	0.961(0.751,1.189)	0.083(0.083,0.084)
Logistic Recalibration-ancillary only	0.784(0.777,0.789)	-0.009(-0.190,0.239)	0.996(0.774,1.246)	0.083(0.083,0.085)

Table S9: Performance metrics for Combination of single Case-mix, Event rate and Predictor-outcome association shifts

Model	AUC	CITL	CSLOPE	Brier Score
Membership-based recalibration	0.784(0.774,0.789)	0.009(-0.344,0.361)	0.807(0.694,0.932)	0.084(0.083,0.087)
Target-only	0.766(0.727,0.779)	-0.005(-0.384,0.380)	0.730(0.539,0.952)	0.087(0.084,0.092)
Intercept Recalibration-all data	0.782(0.775,0.786)	-0.003(-0.358,0.348)	0.754(0.673,0.829)	0.085(0.084,0.088)
Intercept Recalibration-ancillary only	0.784(0.777,0.789)	0.009(-0.364,0.354)	0.745(0.655,0.834)	0.084(0.083,0.088)
Logistic Recalibration-all data	0.782(0.775,0.786)	-0.003(-0.338,0.331)	0.954(0.719,1.239)	0.084(0.083,0.086)
Logistic Recalibration-ancillary only	0.784(0.777,0.789)	0.007(-0.326,0.334)	0.995(0.740,1.289)	0.084(0.083,0.085)

Table S10: Performance Metrics of the Case-Study

Model	Statistic	Optimism-Unadjusted (apparent)	Optimism-Adjusted
Membership-based recalibration	AUC	0.837	0.811
	CITL	0.000	0.059
	CSLOPE	1.356	1.249
	Brier Score	0.002	0.002
Target-only	AUC	0.859	0.792
	CITL	0.000	0.048
	CSLOPE	1.000	0.796
	Brier Score	0.002	0.002
Intercept Recalibration-all data	AUC	0.822	0.815
	CITL	0.000	0.062
	CSLOPE	1.399	1.357
	Brier Score	0.002	0.002
Intercept Recalibration-ancillary only	AUC	0.816	0.813
	CITL	0.000	0.062
	CSLOPE	1.404	1.378
	Brier Score	0.002	0.002
Logistic Recalibration-all data	AUC	0.822	0.815
	CITL	0.000	0.063
	CSLOPE	1.000	1.014
	Brier Score	0.002	0.002
Logistic Recalibration-ancillary only	AUC	0.816	0.813
	CITL	0.000	0.063
	CSLOPE	1.000	1.033
	Brier Score	0.002	0.002

Table S11: Bootstrap Performance Metrics' Confidence Intervals of the Case-Study

Model	Statistic	AUC	CITL	CSLOPE	Brier Score
Membership-based recalibration	Bootstrap Apparent	0.856(0.746,0.946)	0(0,0)	1.325(0.978,1.683)	0.002(0.001,0.003)
	Bootstrap Test	0.83(0.787,0.856)	0.059(-0.439,0.709)	1.219(0.649,1.504)	0.002(0.002,0.002)
Intercept Recalibration-all data	Bootstrap Apparent	0.823(0.7,0.929)	0(0,0)	1.42(0.849,2.009)	0.002(0.001,0.003)
	Bootstrap Test	0.816(0.789,0.837)	0.062(-0.435,0.695)	1.378(1.202,1.55)	0.002(0.002,0.002)
Intercept Recalibration-ancillary only	Bootstrap Apparent	0.813(0.691,0.926)	0(0,0)	1.408(0.744,2.069)	0.002(0.001,0.003)
	Bootstrap Test	0.81(0.78,0.837)	0.062(-0.435,0.695)	1.382(1.209,1.557)	0.002(0.002,0.002)
Logistic Recalibration-all data	Bootstrap Apparent	0.823(0.7,0.929)	0(0,0)	1(1,1)	0.002(0.001,0.003)
	Bootstrap Test	0.816(0.789,0.837)	0.063(-0.452,0.692)	1.014(0.654,1.548)	0.002(0.002,0.002)
Logistic Recalibration-ancillary only	Bootstrap Apparent	0.813(0.691,0.926)	0(0,0)	1(1,1)	0.002(0.001,0.003)
	Bootstrap Test	0.81(0.78,0.837)	0.063(-0.452,0.692)	1.033(0.668,1.655)	0.002(0.002,0.002)
Target-only	Bootstrap Apparent	0.881(0.805,0.95)	0(0,0)	1(1,1)	0.002(0.001,0.003)
	Bootstrap Test	0.814(0.705,0.855)	0.048(-0.448,0.738)	0.796(0.054,1.116)	0.002(0.002,0.002)

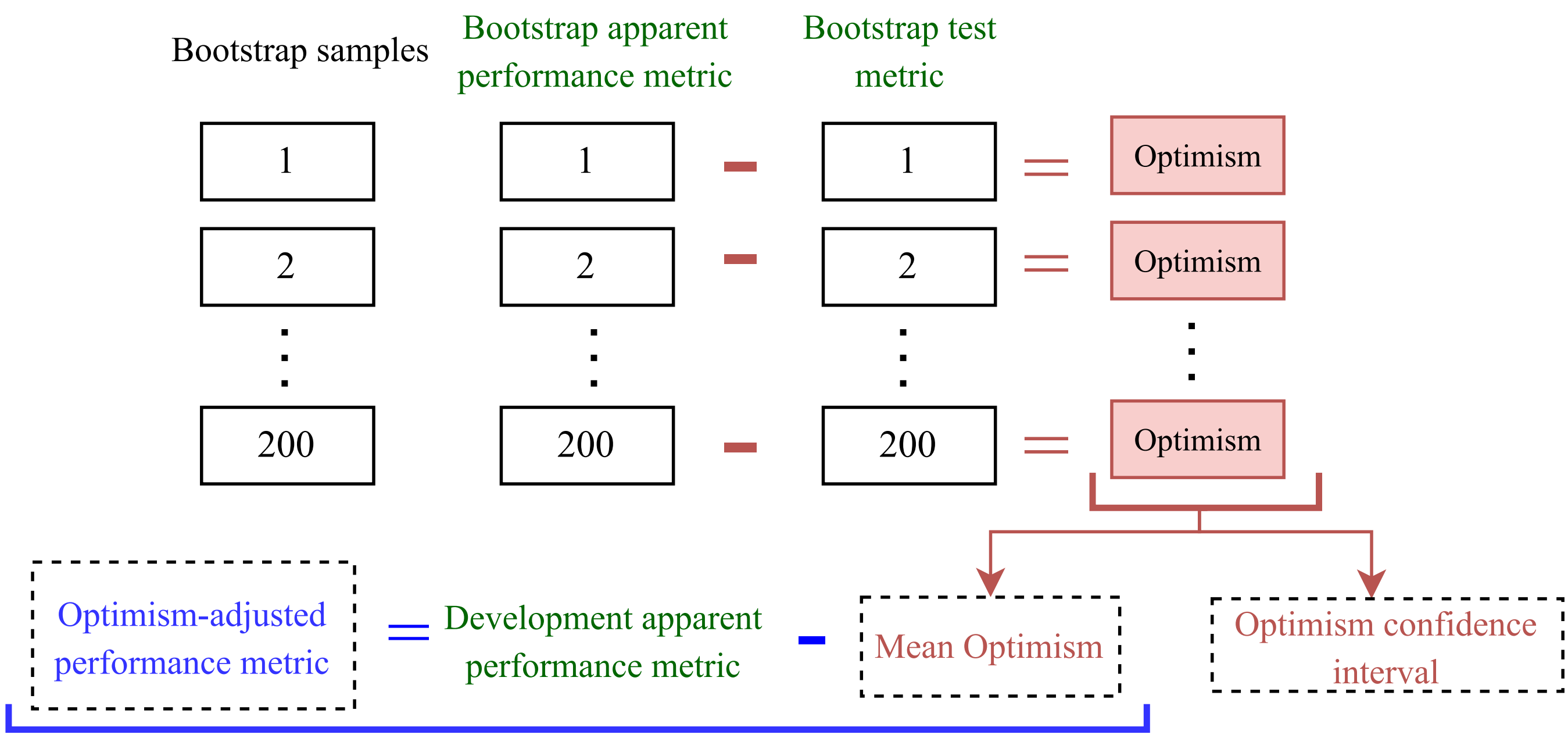

Figure S18: Illustration for Estimating Adjusted-optimism Performance Metric

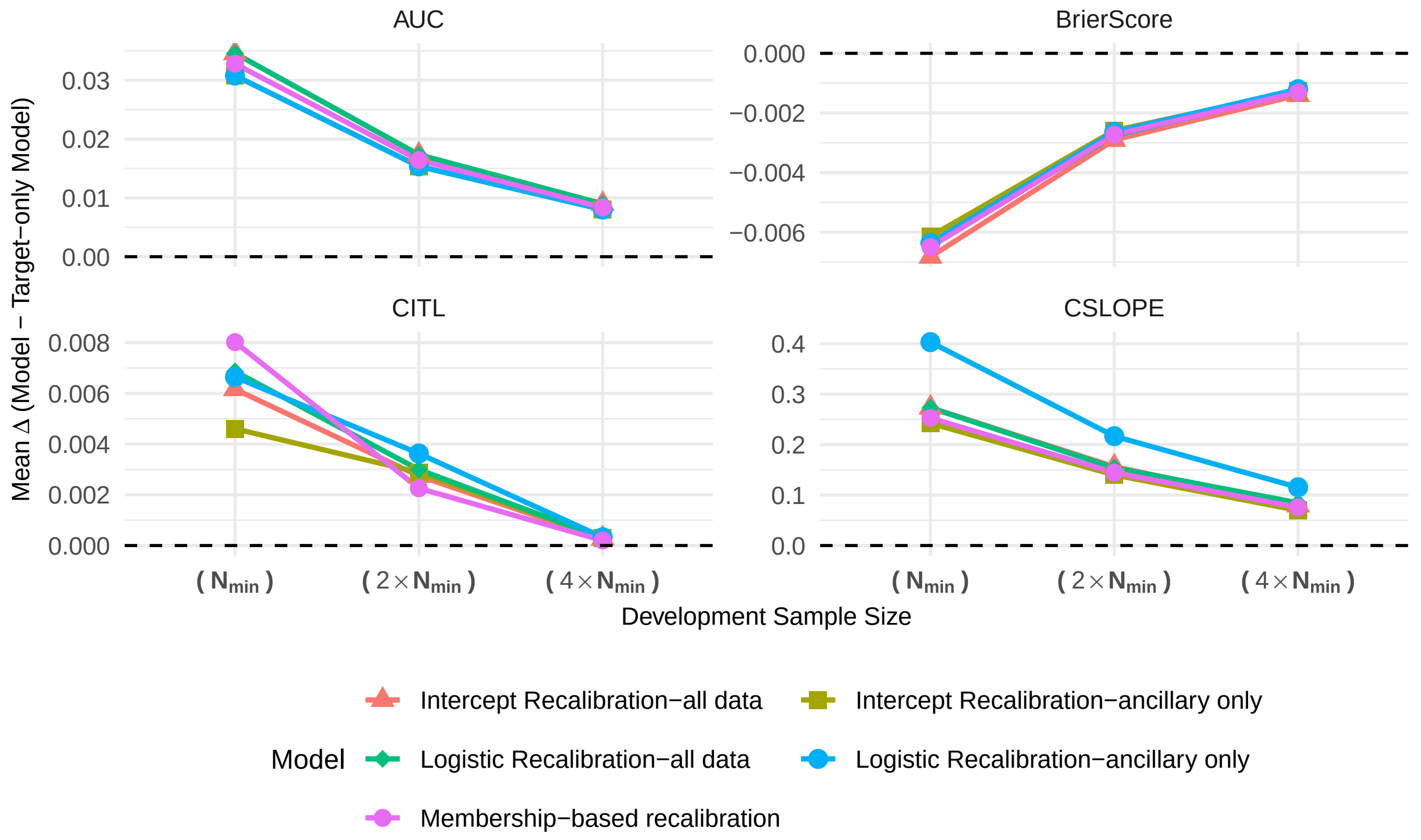

Figure S19: Tipping-point Analysis of Ancillary data benefit across Development Sample Sizes for No Shift. Outcome-generation model with 15 predictors and proportion split is "**75% ancillary**:**25% target**".
The Figure shows the mean Δ across 200 simulation iterations, where Δ represents the difference in performance (AUC, Brier score, CITL, or CSLOPE) between each CPM method and the Target-only model within the same iteration and sample size.

Table S12: Comparison of Discrimination Performance Across Recalibration Methods for Event rate shift

**Model**	**p-value**	**Median AUC**	**ΔAUC**
Target-only	0.000	0.788	-0.0119
Intercept Recalibration-all data	0.000	0.790	0.0004
Intercept Recalibration-ancillary only	0.291	0.790	-0.0001
Logistic Recalibration-all data	0.000	0.790	0.0004
Logistic Recalibration-ancillary only	0.291	0.790	-0.0001

p-value: Two-sided p-value from paired Wilcoxon signed-rank test comparing the model's AUCs across 200 iterations to the Membership-based recalibration; Median AUC: Median AUC across 200 iterations; ΔAUC: difference in median AUC between the model and Membership-based recalibration (ΔAUC = Model – Membership-based).

Outcome-generation model with 15 predictors, total sample size is $4 \times N_{min}$, and proportion split is "**75% ancillary** : **25% target**"

References

[1] H. Elayan, M. Sperrin, G. P. Martin, N. Peek, F. Braunschweig, J. Faxén, J. Alfredsson, and D. A. Jenkins, "Correcting for case-mix shift when developing clinical prediction models," *BMC Medical Research Methodology*, vol. 25, no. 1, p. 186, 2025.

[2] T. P. Debray, Y. Vergouwe, H. Koffijberg, D. Nieboer, E. W. Steyerberg, and K. G. Moons, "A new framework to enhance the interpretation of external validation studies of clinical prediction models," *Journal of clinical epidemiology*, vol. 68, no. 3, pp. 279–289, 2015.
